# Electric Fields in Polymeric Systems

**DOI:** 10.1021/acs.chemrev.4c00490

**Published:** 2024-11-25

**Authors:** Mark A. Rothermund, Stephen J. Koehler, Valerie Vaissier Welborn

**Affiliations:** † Department of Chemistry, Virginia Tech, Blacksburg, Virginia 24060, United States; ‡ Macromolecules Innovation Institute (MII), Virginia Tech, Blacksburg, Virginia 24061, United States

## Abstract

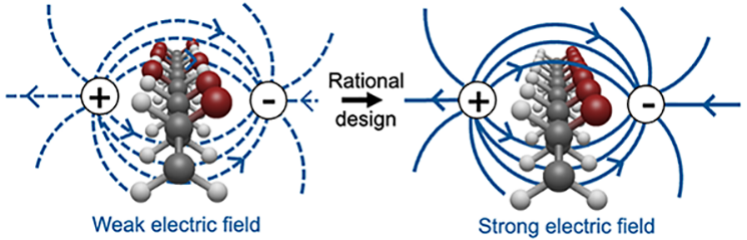

Polymer-based electronic devices are limited by slow transport and recombination of newly separated charges. Built-in electric fields, which arise from compositional gradients, are known to improve charge separation, directional charge transport, and to reduce recombination. Yet, the optimization of these fields through the rational design of polymeric materials is not prevalent. Indeed, polymers are disordered and generate nonuniform electric fields that are hard to measure, and therefore, hard to optimize. Here, we review work focusing on the intentional optimization of electric fields in polymeric systems with applications to catalysis, energy conversion, and storage. This includes chemical tuning of constituent monomers, linkers, morphology, etc. that result in stronger molecular dipoles, polarizability or crystallinity. We also review techniques to characterize electric fields in polymers and emerging processing strategies based on electric fields. These studies demonstrate the benefits of optimizing electric fields in polymers. However, rational design is often restricted to the molecular scale, deriving new pendants on, or linkers between, monomers. This does not always translate in strong electric fields at the polymer level, because they strongly depend on the monomer orientation. A better control of the morphology and monomer-to-polymer scaling relationship is therefore crucial to enhance electric fields in polymeric materials.

## 1 Introduction

Electric fields have been widely adopted in the protein community to rationalize enzyme behavior within the context of electrostatic preorganization theory.^1−6^ Electric fields are also used at an increasing rate to design efficient synthetic enzymes or catalytic constructs.^7−9^ In this case, and in the context of this review, electric fields refer to internal electric fields, also sometimes called local, interfacial, intrinsic or built-in electric fields.^1,10−14^ These electric fields arise from the anisotropy in charge distribution within molecules. Therefore, internal electric fields are nonuniform, following the heterogeneity of the macromolecular environment, which contrasts with the uniform, external, electric fields that can be applied to a system between electrodes.^15−17^

There are two main arguments that make electric fields a powerful tool for molecular design. First, the electric field at a given point in space is proportional to the gradient of the electrostatic potential and, as such, informs on the force exerted on a charge at that location. Electric fields are then directly relevant to reactivity and active processes, improving upon static energy landscapes. Second, electric fields are additive and there exists a natural decomposition into contributions from molecular fragments in the system. Although the language of electric fields has most recently been reserved for the enzyme community, it is not specific to proteins.^7,8,15,18,19^ For example, built-in electric fields have long been part of the characterization of the performance of electronic devices.^20−23^ Indeed, electric fields are responsible for the open circuit voltage, *V*_oc_, indicating how much energy can be stored in a battery, how much voltage can be used in a solar cell, how strong of a reaction can be catalyzed by a photocatalyst, etc.^24−27^ In these devices, electric fields promote charge separation and directional charge transport, pushing electrons and holes toward opposite ends of the device to be collected by the appropriate electrodes.^28−31^ In photoactive devices, electric fields also promote exciton splitting and increase the charge diffusion length, effectively preventing recombination of the newly separated charges.^13,32−34^ In p–n junctions for example, the photovoltaic effect is achieved by creating an electric field across the interface of two materials with opposing affinities for holes and electrons: a p-doped and n-doped semiconductor with different Fermi levels.^20,31,35−37^

**Figure 1:**
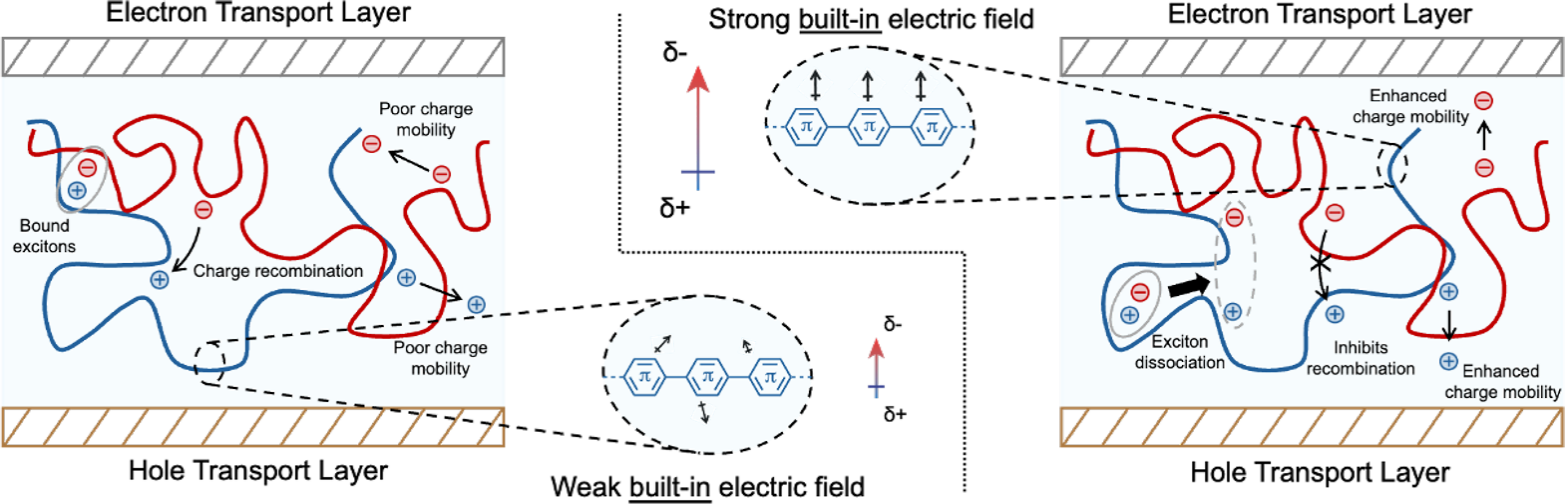
Schematic depiction of a polymer-based electronic device with weak (left) and strong (right) built-in electric fields. Built-in electric fields reduce recombination, a major source of current loss in polymers, and improve exciton splitting as well as directional charge transport. Significant device performance improvement can be achieved by optimizing built-in electric fields in these materials.

In molecular electronics and specifically blended polymer devices, built-in electric fields are weaker over the scale of the device but strong locally, across the maximized, distributed interface where excitons split.^20,38−40^ These local, nanoscale, effects are harder to characterize, which partly explains the secondary role electric fields have taken in designing modern electronics. On the other hand, built-in electric fields offer the opportunity to directly address the fundamental limitations of polymeric materials, namely relatively poor transport properties and high recombination rates. As illustrated in Figure 1, stronger electric fields will reduce recombination and assist directional charge migration across polymer layers, which is indispensable to device performance. Finally, since electric fields directly act on charge migration properties, they have the added advantage of being relevant to all electronic devices, regardless of the application. This makes electric fields a unifying metric for the improvement of electronic device operation.

Ideally, the optimization of electric fields will occur through the rational design of the chemical structure at the molecular scale. This strategy works well in proteins, for example, where single mutations can cause significant increases in local electric fields. For polymeric materials, however, we need better control of the morphology and microstructure, as well as a robust knowledge of the oligomer-to-polymer property scaling. Despite these challenges, many have demonstrated over the years the benefits of using electric fields as an objective function when designing novel materials for catalysis, energy conversion, and energy storage.^41−44^ Indeed, polymers exist with a multitude of lengths, structural, and conformational properties, allowing many strategies for the intentional design of electric fields. These efforts make the basis of this review, which is centered around electric field effects in polymeric systems. In Section 2, we review the distributed multipole expansion often used to rationalize the role of electric fields in molecular systems, as they pertain to molecular dipoles and polarizability. In Section 3, we review work that describes the optimization of electric fields in polymeric materials for electronic devices. This includes chemical tuning of constituent monomers and linkers, as well as the tuning of supramolecular polymer architecture and film morphology. In Section 4, we review innovative polymer types, such as polyelectrolytes, ferroelectric polymer additives, piezoelectric polymers and crystalline polymers, that could further enable the development of polymeric systems that generate strong electric fields. In Section 5, we review key experimental and theoretical approaches to the characterization of nonuniform electric fields in macromolecular systems. Finally, since polymers generating strong electric fields are also more sensitive to applied, external, electric fields, we review in Section 6 emerging strategies for the synthesis and postprocessing of polymeric materials that rely on external fields.

Although impressive device performance improvements are reported in the literature reviewed here, it is evident that the incorporation of electric fields as a metric for the design of novel polymers needs to be accompanied by better prediction, and control, of the morphology of polymeric materials from chemical structure. Indeed, strong dipoles on monomers do not result in strong electric fields when the overarching microstructure of the material randomizes their orientation. Similarly, emerging polyelectrolyte or piezoelectric polymers seem naturally suited to the generation of electric fields, but more work is needed to provide robust design rules. Finally, the field would benefit from characterization techniques to measure the local strength and orientation of electric fields on polymers, complementing those measuring electric fields across a device.

## 2 Theory

An electric field is a vector field that arises from the anisotropy in the charge distribution of the system. The electric field vector is proportional to the gradient of the electrostatic potential (eq 1), which quantifies the amount of work needed to move a unit charge from a reference point to a specific location.^45−47^
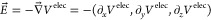
1where *E⃗* is the electric field, *V*^elec^ the electrostatic potential, and we use the shorthand notation  for partial derivatives. Eq 1 shows that the electric field vector is oriented toward regions of lower potentials. At a given location, the electric field exerts a force on charged particles, proportional to the magnitude of the charge:
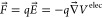
2where *F⃗* is the force experienced by the charge *q*. Therefore, a positive (negative) charge experiences a force in the (opposite) direction of the electric field.

In polymers and other macromolecular systems, the electrostatic potential, and therefore the electric field is nonuniform.^15−17,48^ Anthony Stone developed the distributed multipole approach to characterize the inherent anisotropy of charge distribution in molecular systems.^49−51^ We briefly outline below the key steps in the distributed multipoles expansion of the electrostatic potential.

The electrostatic potential arising at *R⃗* = (*R*_*x*_, *R*_*y*_, *R*_*z*_) from a point charge, *q*, located at *r⃗*, is given by
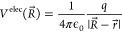
3If we now assume that this charge *q* is the added effect of a collection of other charges, say partial charges at atomic positions, the electrostatic potential becomes
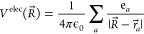
4where the summation is carried over the *a* partial charges *e*_*a*_ located at *r⃗*_*a*_ = (*r*_*ax*_, *r*_*ay*_, *r*_*az*_) and
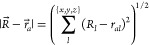
5

A Taylor expansion of the electrostatic potential about the origin (i.e., where *r⃗*_*a*_ = 0), truncated at the second order, gives
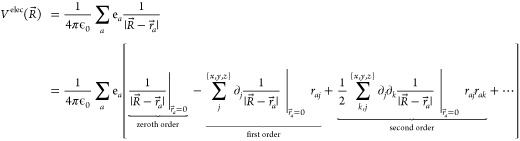
6The zeroth order term is simply:
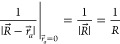
7The first order derivative is
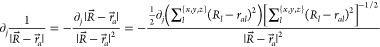
8which is nonzero only when *j* = *l*, and we get:
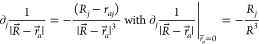
9

For *k* ≠ *j*, the second order derivative is (the order does not matter):
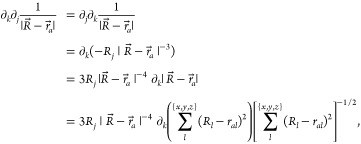
10which is nonzero only when *l* = *k*, and we obtain
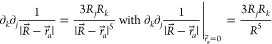
11For *k* = *j*, the second order derivative is
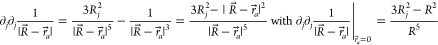
12Eqs 11 and 12 can be combined into one equation defining the elements of a (3 × 3) traceless matrix:
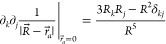
13Overall, it yields for the electrostatic potential:
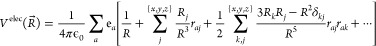
14Considering that the charge, dipole and quadrupole moments can be defined as^50,52−56^
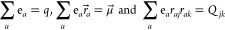
15we can write
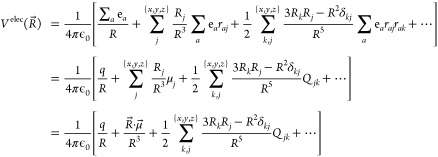
16Note that since the second derivative is traceless (eq 13), we can use traceless quadrupole moments without a change to the equations.^50,53^

From eq 16, we see that, for neutral molecules where *q* = 0, the dipole term is the leading term. However, that term is a dot product with *R⃗*, meaning that the magnitude of the dipole will increase the electrostatic potential only if its orientation matches that of *R⃗*. This applies to the quadrupole term as well: at constant electric moments, changes in geometry (i.e., changes in *R⃗*) will yield significant changes in the electrostatic potential and corresponding electric field. This implies that even if the observable, electric field, is electrostatic in nature, it accounts for a multitude of other effects through the geometry of the system. For example, if the hydrogen bonding network is disrupted, it will affect the relative orientation of the bond donor and acceptor, which in turn, will affect the electrostatic potential. A similar statement can be made about solvation, polarization, and entropic effects, to name a few. In practice, changes in geometry are exacerbated by, but not solely based on, changes in charge distribution within the molecules (through partial charges, dipoles and quadrupoles). This is what makes electric fields a powerful and general metric for material design: every intermolecular and intramolecular interaction will have a signature on the electrostatic potential and underlying electric field.

Eq 16 defined the electrostatic potential at an arbitrary point. If we now consider that another molecule, say M2, is located at *R⃗*, its own set of charge (*q*^*M*2^), dipole (*μ⃗*^*M*2^) and quadrupole (*Q*^*M*2^) will interact with the electrostatic potential generated by the other molecules in the system. In particular, the interaction energy between a molecule M1 at *r⃗* and M2 at *R⃗* can be written as
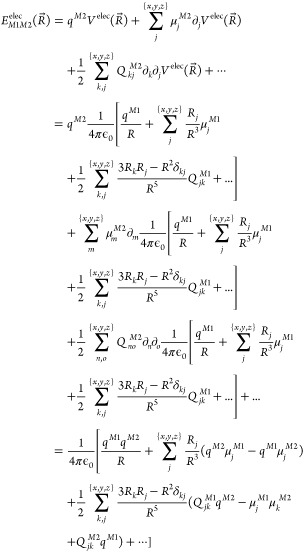
17Similarly as in eq 16, eq 17 shows that in neutral species where the charge adds up to zero, the interaction energy is dominated by the dipole terms, including the dipole–dipole interaction term:
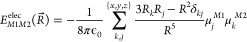
18In addition, if M2 is polarizable, the electric field arising from the other molecules also creates an induced dipole at *R⃗*:
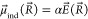
19where α is the polarizability matrix. This means that even apolar (but polarizable) molecules are sensitive to electric fields. The new induced dipole at *R⃗* in turn interacts with the original dipole at *r⃗* through eq 17, for a mutual polarization of all molecular fragments in the environment.

In summary, the magnitude of the electric fields generated by molecular fragments, polymer constituents in our case, can be increased through greater crystallinity (dependence on *R⃗*), greater charge imbalance (dependence on electric moments) or greater polarizability (dependence on induced dipoles).^57,58^ In the following sections, we will review published work that addresses one or several of these factors.

## 3 Enhancing Electric Fields in Polymeric Materials for Electronic Devices

Open-circuit voltage (*V*_oc_)—the voltage difference across a device when it is not connected to any circuit—is a key performance characteristic of electronic devices. A high *V*_oc_ indicates that positive and negatives charges are separated and efficiently transported to opposite electrodes, where they are collected. *V*_oc_ scales with application-specific performance metrics, such as power conversion efficiency (PCE) in solar cells,^30,59^ energy storage in batteries,^60^ intensity or efficiency of light emission in light-emitting diodes (LEDs) and organic LEDs (OLEDs),^61^ photocatalyst activity,^24,29,33,62−66^ and water splitting efficiency in electrochemical cells.^33,63^

Built-in electric fields are an effective way to achieve high *V*_oc_ because it directs positive and negative charges in opposite directions and increases the charge diffusion length within a material (see Introduction).^21,25,32,67^ In photoactive materials, built-in electric fields also assist exciton dissociation, converting bound electron–hole pairs into separated free charge carriers.^57,68−72^ Strong built-in electric fields are especially important to enhance the *V*_oc_ of polymer-based materials where geminate recombination of photogenerated charge carriers occurs frequently, limiting device efficiency.^69,71^ In this section, we will review work that has been done to improve built-in electric fields in polymeric materials. This includes modifications of photoactive layers as well as interface engineering in organic photovoltaics, LEDs, and other electronic devices. Since built-in electric fields are relevant to all electronic devices, the section is organized by strategy employed to increase the fields, rather than by application. To facilitate navigation to those interested in the applications, we summarized the key papers in Table 1.

**Table 1:** List of Systems and Improvements Due to Electric Fields Reported in the Papers Reviewed in Section 3, by Application and by Strategy^a^

Strategy	Application	System	Reported improvement
Monomer design (Section 3.1)	Photocatalyst for H_2_ production	PCPs^65^	μ ∼ 1.5 D, photocatalytic activity ×10
		CMPs^29,73^	μ + 3.8 D, Yield ×1.5
		TPPS/PDI^74^	Efficiency ×10
	Active layers in solar cells	PTB7^75^	*V*_oc_ + 0.1 V, Efficiency ×3.6
		PTBF1,^76^ DTFFBT^77^	*V*_oc_ + 0.1 V, Efficiency +2%
		P1, P2^78,79^	Highest efficiency to date (16%)
	Electrode interlayers	p-PFP/PFN^30^	Efficiency +1.3%
		PBTA-FN^80^	Elec. field +0.1 V
		PDIN-N-FN^59^	Efficiency +2.7%
Linker design (Section 3.2)	Photocatalyst for H_2_ production	MNBN1, PNBN^81^	μ + 7.3 *D*, surface potential ×8
		PDI linkers^33,62^	μ + 4.8 *D*, photocurrent ×30
		H-bond PDI linkers^64^	Photocatalytic activity ×8
	Active layers in solar cells	DPP, DPP-DTP^82−84^	Exciton binding energy –0.4 eV
		Y6 derivatives^85,86^	Highest efficiency to date (17.2%)
Supramolecular architecture tuning (Section 3.3)	Lithium batteries	NT-U/NDI^39^	Elec. field ×7.3, discharge capacity ×3
		CP-PDAB,^87^ polyimide^88^	High specific capacity (>140 mAh g^–1^)
		PPTS^89^	5000 charge–discharge cycles
	Photocatalysis	PcOp-Fe^38^	Surface potential +180 mV
		Porphyrin complexes^74,90^	Electric field ×10
		PDI/BiOCl^91^	Efficiency ×2

a The stronger electric fields were linked to enhanced charge separation, improved conductivity, reduced recombination rates and lowered exciton binding energy (see also Figure 1), which, in turn, improved overall device performance.

### 3.1 Enhancing Electric Fields by Monomer Design

**Figure 2:**
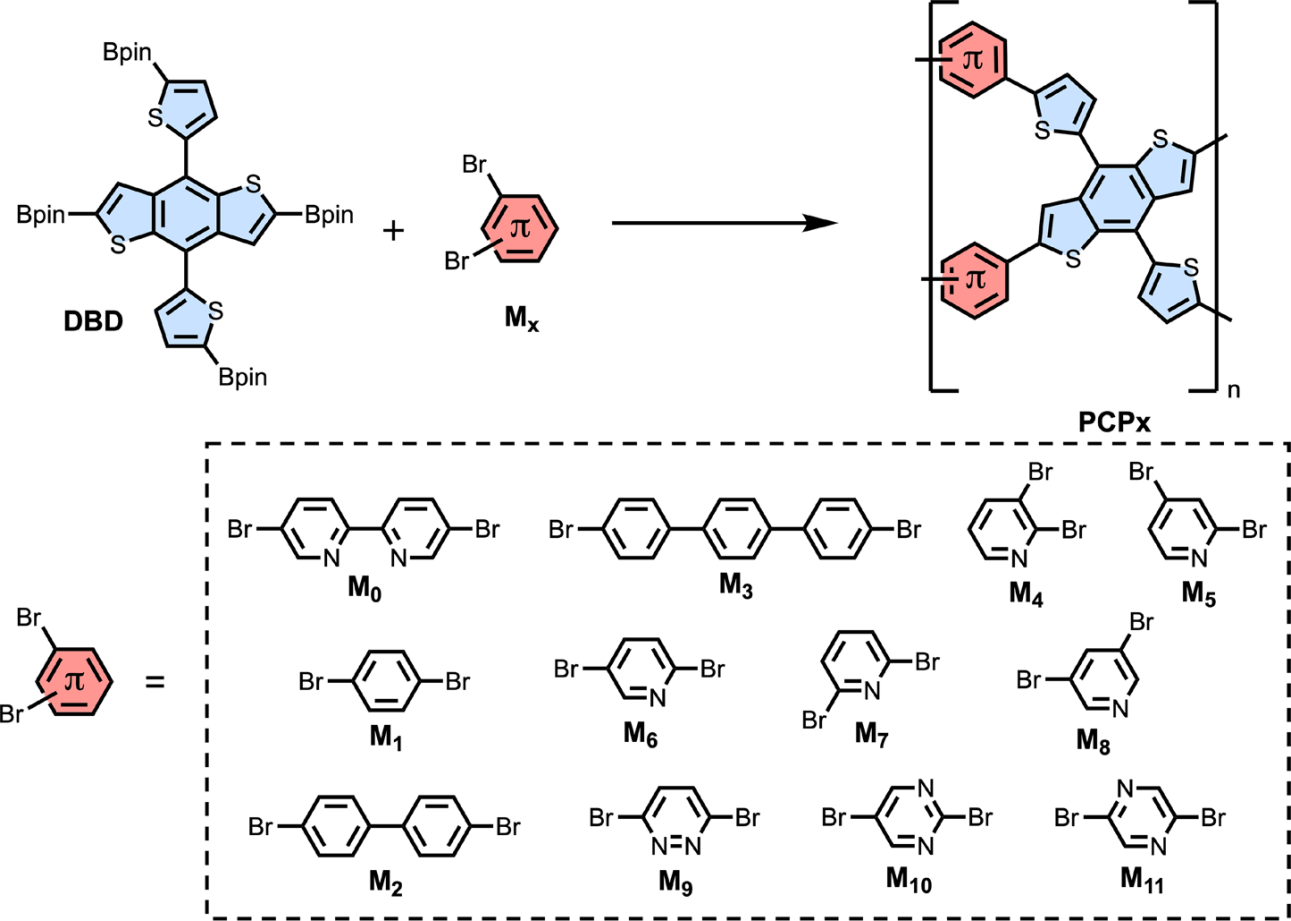
Scheme showing the structures of comonomers (M_0_–M_11_) and DBD which were used to prepare polymers PCP0–PCP11. Adapted with permission from Li, L.; Lo, W.-y.; Cai, Z.; Zhang, N.; Yu, L. Donor–acceptor porous conjugated polymers for photocatalytic hydrogen production: the importance of acceptor comonomer. *Macromolecules***2016**, *49*, 6903–6909. Copyright 2016 American Chemical Society.

A rational strategy to enhance a polymer’s internal electric field is through selective tuning of its monomers. As detailed in Section 2, this can be achieved by designing functional groups that will increase the electric moments of the monomers (e.g., charge, dipole, quadrupoles, etc.) or the polarizability. For example, Li et al. emphasized the importance of tuning the internal polarization of acceptor comonomers when constructing donor–acceptor copolymers as photocatalysts for hydrogen production.^65^ They designed sets of porous conjugated polymer (PCP) networks using 12 different acceptors with a 4,8-di(thiophen-2-yl)benzo[1,2-b:4,5-b’]dithiophene (DBD) donor, as shown in Figure 2. They found that the best performing PCPs were those containing ligands with varied nitrogen atoms such as pyridine and diazine. They attributed this effect to a stronger internal polarization, therefore a stronger dipole (see eq 19), enabling effective charge separation.^65^ PCP10 and PCP11 exhibited the highest activities with 103.6 and 106.9 μmol h^–1^, respectively, compared to 1.9–10.1 μmol h^–1^ for PCP0–PCP3. When comparing the activity of PCP10 and PCP11 to the dipoles of the comonomers, they found that an appropriate range for the dipole was 1.10–1.65 D. However, they also noted that the orientation of the dipole was key when PCP9, despite having a 3.53 D dipole, did not show higher catalytic activity (30.4 μmol h^–1^) than PCP10 and PCP11.^65^

**Figure 3:**
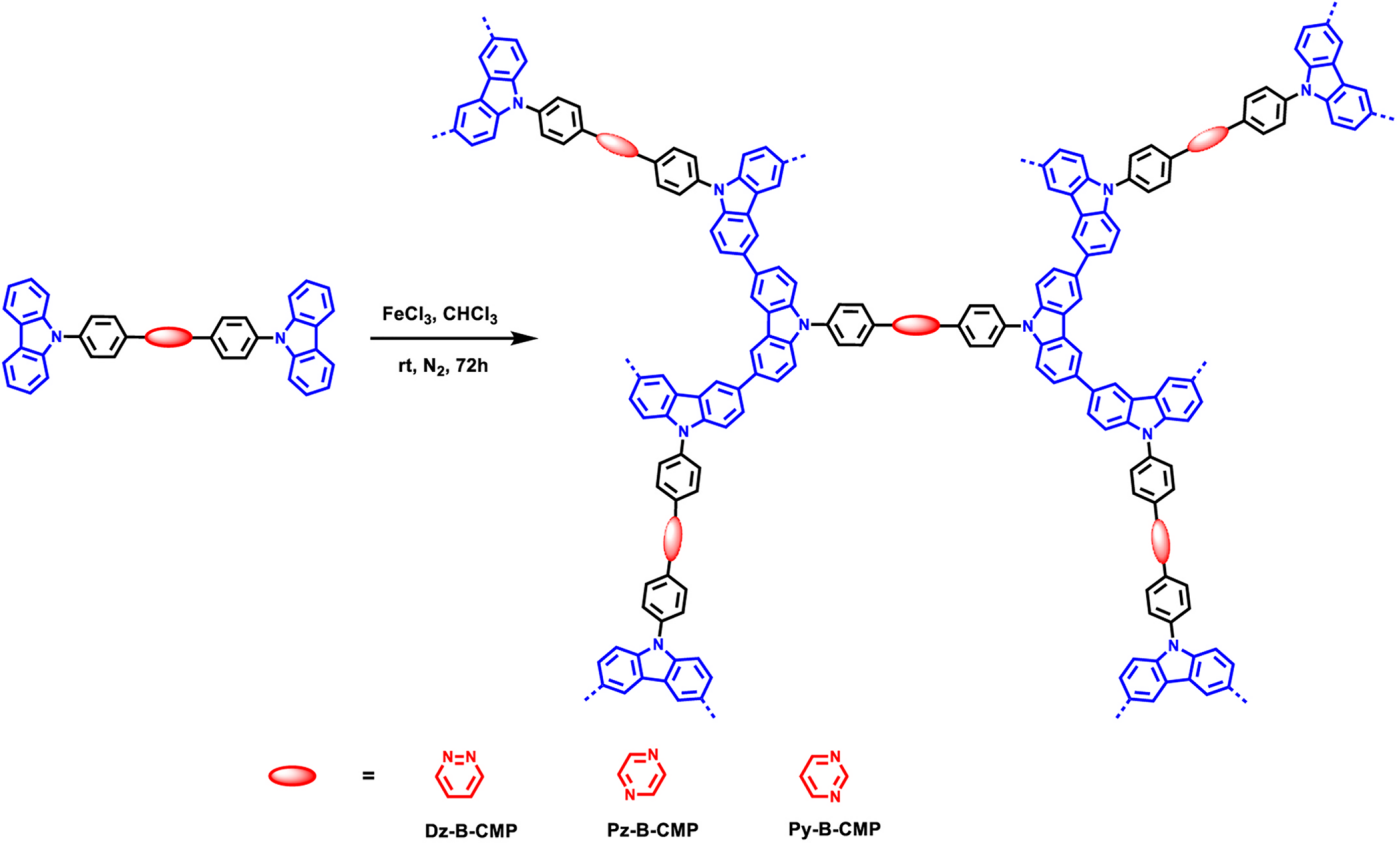
Scheme showing the synthesis of CMPs with the varied acceptors shown in red, π-bridges (benzene) in black, and carbazole in blue. Reproduced with permission from ref (93). Copyright 2024 Elsevier.

**Figure 4:**
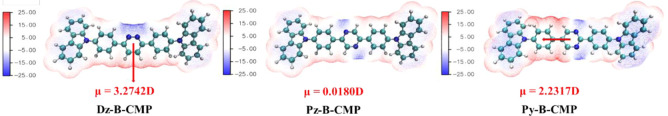
Dipole moments and molecular electrostatic potential maps of the CMP monomers. Adapted with permission from ref (93). Copyright 2024 Elsevier.

Xu et al. were also successful in improving hydrogen evolution rates by modulating the ratio of pyrene to benzothiadiazole units in a series of donor-π-donor (D-π-D), donor-π-acceptor (D-π-*A*), and π-acceptor (π-*A*) conjugated microporous polymers (CMPs). They find the highest rates among the D-π-*A* CMPs (106.2 μmol h^–1^), and the lowest rates among the π–*A* CMPs (0.5 μmol h^–1^).^73^ They did not directly attribute their findings to dipole or electric field effects, instead focusing on increased rates brought on by scattering due to the porous nature of the materials.^73^ Still, structures like the D-π-*A* system have been implicated in other studies for their ability to increase electric fields.^92,93^ For example, Wang et al. expanded on the study by Xu et al., investigating the modulation of D-π-*A* CMPs through the acceptor building block as photocatalysts for the synthesis of benzimidazole.^93^ Varying the acceptor block between pyridazine (Dz), pyrazine (Pz) and pyrimidine (Py), using benzene (B) as the π-bridge, and carbazole as the donor group (Figure 3), they found that Py-B-CMP produced the highest yield (95.5%) compared to Pz-B-CMP (30%) and Dz-B-CMP (21%). They attributed the superior performance of Py-B-CMP to the built-in electric fields resulting in increased photogenerated carrier separation.^93^ Although the magnitude of the dipole of the Dz-B-CMP monomer (3.27 D) was much larger than those of Py-B-CMP (2.23 D) and Pz-B-CMP (0.018 D), the electric field of Dz-B-CMP was perpendicular to the molecule, hindering separation.^93^ In contrast, Py-B-CMP had a dipole direction along the polymer chain, which assisted in separation and migration of electron hole pairs along the chain (Figure 4).^93^

**Figure 5:**
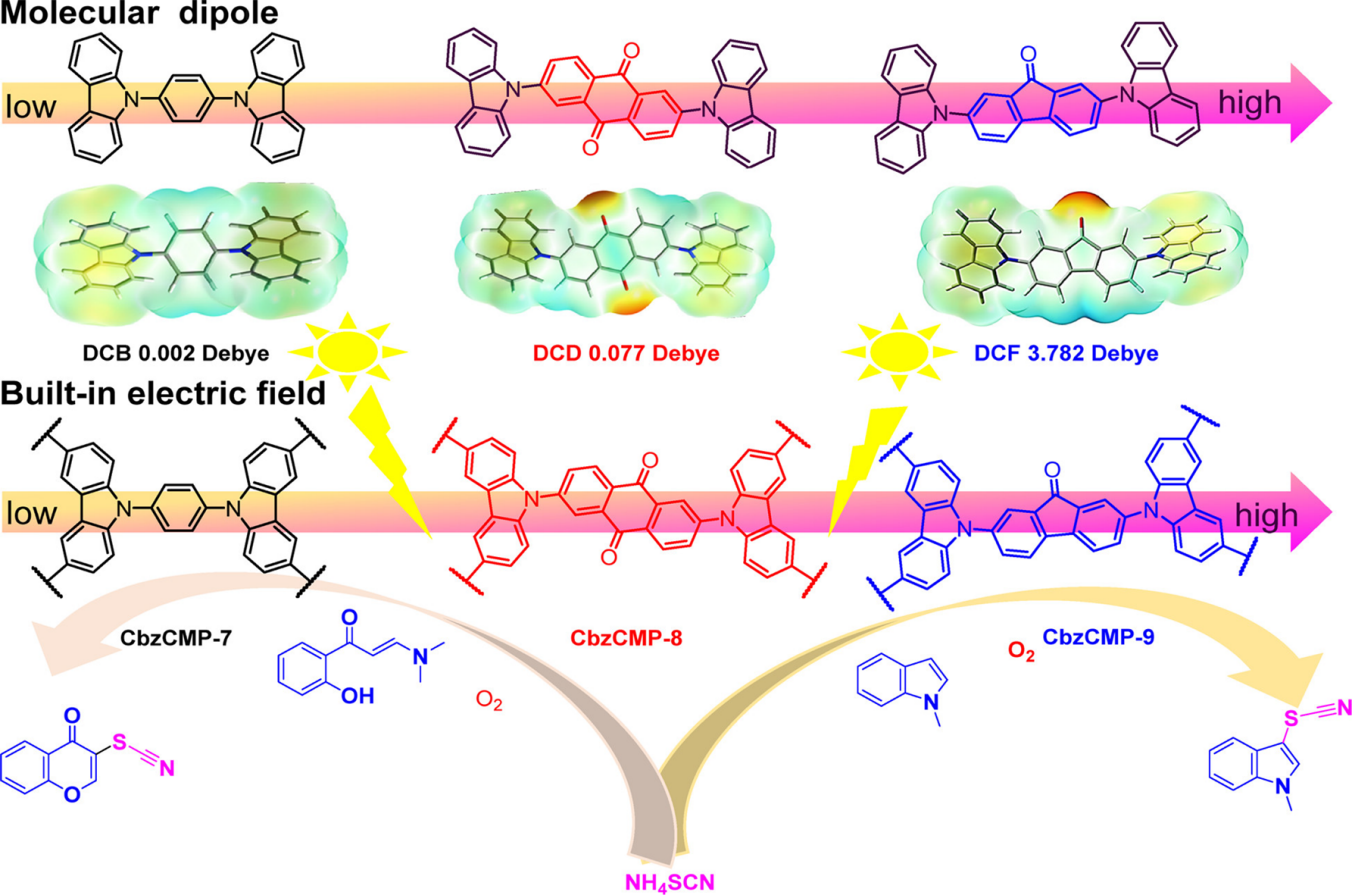
Scheme showing the tested polymers along with calculated dipoles and general catalytic mechanism. Reprinted (adapted) with permission from Deng, Z.; Zhao, H.; Cao, X.; Xiong, S.; Li, G.; Deng, J.; Yang, H.; Zhang, W.; Liu, Q. Enhancing built-in electric field via molecular dipole control in conjugated microporous polymers for boosting charge separation. *ACS Appl. Mater. Interfaces***2022**, *14*, 35745–35754. Copyright 2022 American Chemical Society.

In another proof-of-concept study, the monomer core structure of CMPs that had poor photocatalytic activity due to high exciton binding energies was modified to increase molecular dipoles and built-in electric fields.^29^ The carbazole-based monomers 1,4-di(9H-carbazol-9-yl) benzene (DCB), (2,6-di(9H-carbazol-9-yl) anthracene-9,10-dione (DCD), and (2,7-di(9H-carbazol-9-yl)-9H-fluoren-9-one (DCF) were polymerized to form the photocatalytic polymers denoted as CbzCMP-7, CbzCMP-8, and CbzCMP-9 respectively, shown in Figure 5. Using density functional theory (DFT) they calculated the dipoles of the monomers to be 0.0021, 0.0776, and 3.7819 D, respectively, and found that they correlate with the relative built-in electric field intensity observed experimentally.^29^ Overall, the yield for the photocatalytic construction of the thiocyano chromones was improved to 95% for CbzCMP-9 (highest dipole), 64% for CbzCMP-7 (lowest dipole), and 82% for CbzCMP-8.^29^

**Figure 6:**
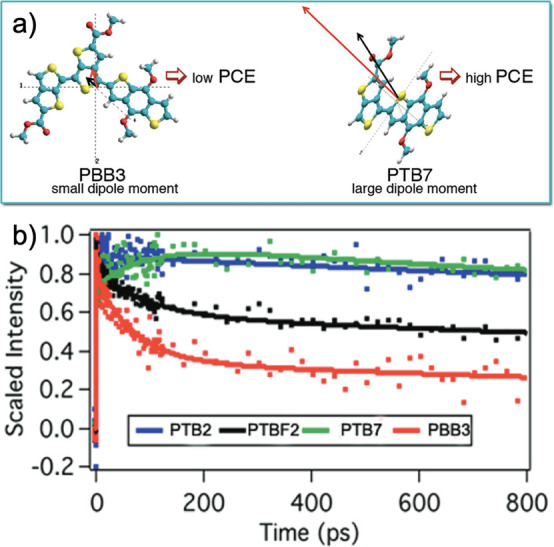
A scheme representing the influence of monomer/polymer dipole on power conversion efficiency. Reprinted (adapted) with permission from Carsten, B.; Szarko, J.M.; Son, H.J.; Wang, M.; Lu, L.; He, H.; Rolczynski, B.S.; Lou, S.J.; Chen, L.X.; Yu, L. Examining the effect of the dipole moment on charge separation in donor–acceptor polymers for organic photovoltaic applications. *J. Am. Chem. Soc.***2011**, *133*, 20468–20475. Copyright 2011 American Chemical Society Copyright 2011 American Chemical Society.

In a similar vein, Carsten et al. investigated how the dipole of a polymer influences its performance as the active material in a solar cell.^75^ They predicted that poly(bi[thieno[3,4-*b*]thiophenyl]-2,2′-dicarboxylic acid bis(2-butyl-octyl) ester-*co*-di(2-butyl-octyl)benzo[1,2-*b*:4,5-*b*’]dithiophene) (PBB3) and poly(3-fluoro-thieno[3,4-*b*]thiophene-2-carboxylic acid 2-butyl-octyl ester-*co*-di(2-butyl-octyl)benzo[1,2-*b*:4,5-*b*’]dithiophene) PTB7 had the smallest and greatest change in ground state to excited state dipole moment (0.47 and 3.92 D, respectively), defined as follows:
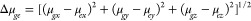
20where Δμ_*ge*_ is the change in dipole moment from the ground state to excited state, μ_*gx*,*y*,*z*_ and μ_*ex*,*y*,*z*_ are each of the directional vector components of the ground state and excited state dipole moments, respectively. They reported the *V*_oc_ of PTB7 at 0.74 V and its PCE at 7.40%, whereas the *V*_oc_ of PBB3 was 0.63 V and its PCE 2.04%.^75^ PBB3 also exhibited the fastest charge recombination rate when analyzed using transient absorption spectroscopy (Figure 6). Conversely, PTB7 showed a long-lived state that could not be measured on the time scale of the experiments.^75^ Overall, the work by Carsten et al. supports the fact that the increased dipole of PTB7 generates a stronger built-in electric field in the polymeric active layer, which reduces charge recombination and increases the performance of the solar cell.

**Figure 7:**
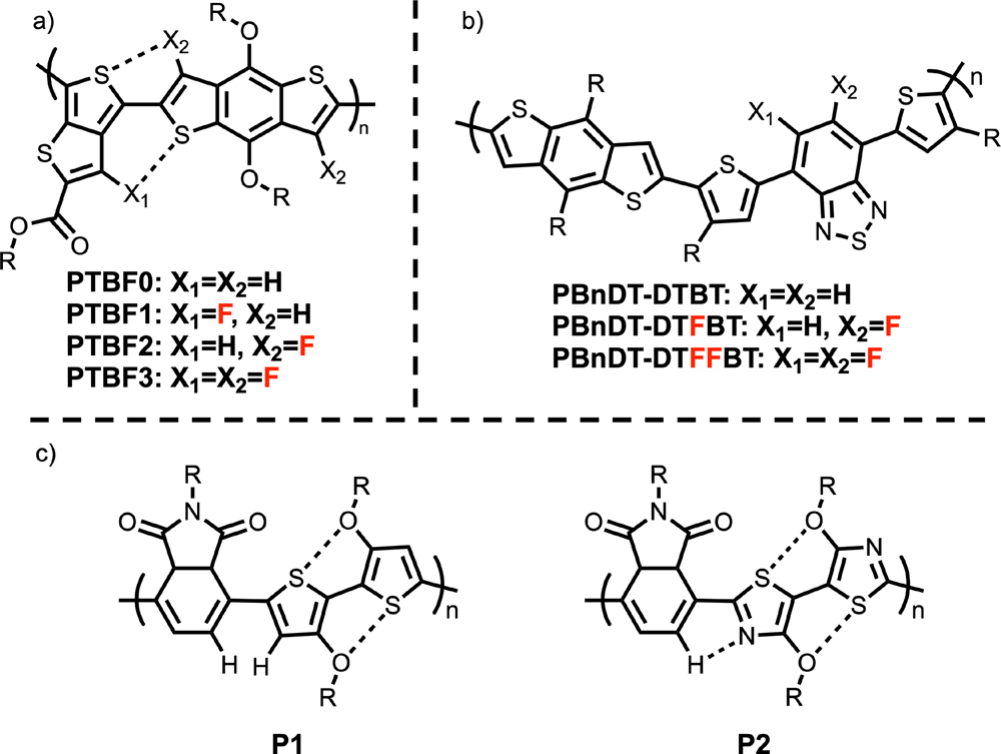
Polymers based on (a) thienothiophene,^76^ (b) benzthiadiazole acceptors,^77^ and their fluorinated analogues. (c) Examples of alokxy substitued bithiophenes and bithiazoles.^78^

The same research group expanded upon the above work, investigating poly(thienothiophene-*co*-benzodithiophene) with varied fluorination, as shown in Figure 7a.^76^ PTBF2 and PTBF3 were predicted by DFT to have the smallest inter-ring dihedral angle (179.5° and 179.8°, respectively), owing to sulfur–fluorine interactions. When cast as films from dichlorobenzne/1,8-diiodooctane, PTBF1 demonstrated the highest PCE of 7.2% and a *V*_oc_ of 0.74 V, both of which exceeded PTBF0 (5.1% and 0.58 V, respectively).^76^ However, despite their increased planarization, PTBF2 and PTBF3 demonstrated a lower PCE (3.2% and 2.3%, respectively) than PTBF1 and PTBF0. Transmission electron microscopy revealed phase separation between PTBF2/PTBF3 and PC_71_BM with domains ranging from 50 to 200 nm, which far exceeds the expected exciton diffusion length of 10 nm. This phase separation was attribtuted to the increased fluorine content making it less compatible with PC_71_BM than PTBF0 or PTBF1. In parallel, Stuart et al. designed variably fluorinated benzothiadiazole polymers, as shown in Figure 7b.^77^ They found that Δμ_*ge*_ increased with fluorination, yielding 1.20 D for polymers without fluorines, 15.18 D for the monofluorinated polymer, and 16.02 D for the difluorinated polymer. They also reported the difluorinated polymer to have the greatest *V*_oc_ (0.90 V), PCE (6.64%), and the least geminate recombination. By comparison, the unfluorinated polymer yielded *V*_oc_ = 0.78 V, PCE = 4.33%, and the most geminate recombination.^77^

Guo et al. investigated alkoxybithiazole monomers with a similar motivation.^78^ The inter-ring dihedral angle of the alkoxybithiophene monomer (P1) was predicted to be 23°, whereas the alkoxybithiazole (P2) was predicted to be 0°, facilitated by a weak C–H–N hydrogen bond in addition to the S–O interaction that is present in both (Figure 7c).^78^ Recently, Wu et al. utilized these alkoxybithiazole comonomers toward all-polymer solar cells.^79^ They observed similar results, where the alkoxybithiazoles (0.3°–0.7°) increased the planarity compared to bithiophene (8.8°–11.6°) and exhibited even less geminate recombination. This enabled the highest performing printed all polymer solar cell for its time (in 2024) with a PCE of 16.0%.^79^

The above studies reported on innovations to increase the dipole moment of polymer constituents to enhance electric fields in the photoactive layers of electronic devices. Others have instead engineered better interlayers between the photoactive material and the electrodes. Interlayers are routinely used in polymer solar cells, tuning the Fermi level of the metal electrodes and improving the collection efficiency of the photogenerated charges. Interlayers are charge transporting layers (CTLs), traditionally composed of conducting polymers like poly(3,4-ethylenedioxythiophene) doped with poly(styrene-sulfonate) (PEDOT:PSS)^94−97^ or metal oxides.^20,98−100^ Lee et al. conceived a new type of paired electric dipole layers (EDLs), to serve as cathode and anode interlayers, with conjugated polyelectrolyes (CPEs) and non-CPE polymers.^30^ For the anode interlayer, they tested CPEs based on p-doped poly[9,9-bis(4′-sulfonatobutyl)fluorene-*alt*-1,4-phenylene] (p-PFPs). The designed polymers varied in self-doping levels, reported as p-PFP-WD, p-PFP-MD, p-PFP-HD, for weakly (WD), moderately (MD), and highly (HD) doped, and p-PFP-O containing phenyl-OMe as a tethering functional group (O). For the cathode interlayer, they compared amine-based CPE poly[(9,9-bis(3′-(*N,N*-dimethylamino)propyl)-2,7-fluorene)-*alt*-2,7-(9,9-dioctylfluorene)] (PFN) and non-CPEs polyallylamine (PAA) and polyethyleneimine (PEI). They found that paired EDLs maximized the electric field across the photoactive layer, and therefore the *V*_oc_ of the corresponding solar cell. Ultimately, they were able to construct a polymer solar cell with 9.8% PCE, which improves significantly upon the 8.5% PCE of the solar cells made with traditional CTLs.^30^

Similarly, Liu et al. aimed to optimize the efficiency of organic solar cells with an alcohol-soluble conjugated polymer named PBTA-FN.^101^ PBTA-FN is derived from poly[(9,9-bis(3′-(N,N-dimethylamino)propyl)-2,7-fluorene)-alt-2,7-(9,9-dioctylfluorene)] (PFN), a polymer previously reported as successful cathode interlayer in diodes,^80^ with fluorene and benzotriazole (BTA) groups. They found that the electron-deficient and amino groups in PBTA-FN yield larger interface dipoles compared to PFN. Consequently, they measured an increase in built-in electric field across the device, going from 0.72 V for PFN to 0.81 V for PBTA-FN, which inhibits bimolecular recombination and enhances device performance.^101^

The same research group later designed other cathode interlayers using self-doping CPEs based on naphthalenediimide (NDI).^59^ They systematically modified the NDI-based monomer pendant groups to synthesize three CPEs: poly[(2,7-bis(2'-butyloctyl)naphthalenediimide-4,9-diyl)-*alt*-(9,9-bis(3-*N,N*-dimethylaminopropyl)-9H-fluorene-2,7-diyl)] (PNDI-FN), poly[(2,7-bis(3-(dimethylamino)propyl)naphthalenediimide-4,9-diyl)-*alt*-(9,9-dioctyl-9H-fluorene-2,7-diyl)] (PNDI-N-F) and poly[(2,7-bis(3-(dimethylamino)propyl) naphth-alenediimide-4,9-diyl)-*alt*-(9,9-bis (3-*N,N*-dimethylaminopropyl)-9H-fluorene-2,7-diyl)] (PNDI-N-FN). They report that modulating the polymer dipoles through the number and position of polar groups boosted built-in electric fields, improving conductivity, work function tunability and interfacial interactions. The solar cells made from these CPEs exhibited PCEs of 8.27% (PNDI-FN), 8.48% (PNDI-N-F), and 9.01% (PDIN-N-FN), a significant increased compared to the PCE of solar cells in absence of CPEs (6.31%).^59^

Yang et al. boosted photocatalytic H_2_ evolution by constructing an electron donor–acceptor (D-A) interface between tetra(4-sulfonatophenyl)porphyrin (TPPS) and PDI, which resulted in an evolution rate of 546.54 μmol h^–1^, 9.95 times higher than pure TPPS and 9.41 times higher than PDI.^74^ The interfacial electric field from PDI to TPPS facilitates charge transfer, increasing exciton separation efficiency and hydrogen production.^74^ The internal electric field was found to be 3.76 times higher in TPPS/PDI than in pure PDI and 3.01 times higher than pure TPPS.^74^

Hwang et al. used ultrafast electronic spectroscopy to monitor the formation of charge-transfer excitons in two conjugated polymers.^102^ The absorption for poly(*N*-11″-henicosanyl-2,7-carbazole-*alt*-5,5-(4′,7′-dithienyl-2’,1’,3′-benxothiadiazole) (PCDTBT) red-shifted in a more polar solvent, which suggests an increase in the dipole moment through eq 21:
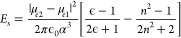
21where μ_*e*2_ and μ_*e*1_ are the equilibrium and nonequilibrium dipole moments respectively, ϵ_0_ is the permittivity, *a* is the interaction cavity radius, ϵ is the dielectric constant, and *n* is the refractive index. From this they found that the change in polymer dipole changes by 3.3 D upon excited state equilibration, which was corroborated by changes in the excited state structure using DFT.^102^ While not explicitly measured in the above study, they allude to the change in dipole explaining why PCDTBT-based solar cells demonstrate efficiency without an exogenous acceptor.^103^ Cheng et al. also leveraged ultrafast spectroscopy to study the electron dynamics in a friedel-crafts polymerized dibenzothiophene.^104^ Upon photoexcitation, the sulfur atoms can spontaneously oxidize in the presence of air to form sulfones, changing from a homopolymer to a donor–acceptor copolymer *in situ*. As such, the dipole moment was predicted to increase from 0.62 to 6.17 D by DFT, and this helped facilitate charge separation as determined by TAS.^104^ Upon this change they also observe an increase in the photocatalytic production of H_2_O_2_ from 841 μM h^–1^ (homopolymer) to 1321 μM h^–1^ (donor–acceptor copolymer), which they attribute to the change in structure and its impact on properties.

### 3.2 Enhancing Electric Fields by Linker Design

**Figure 8:**
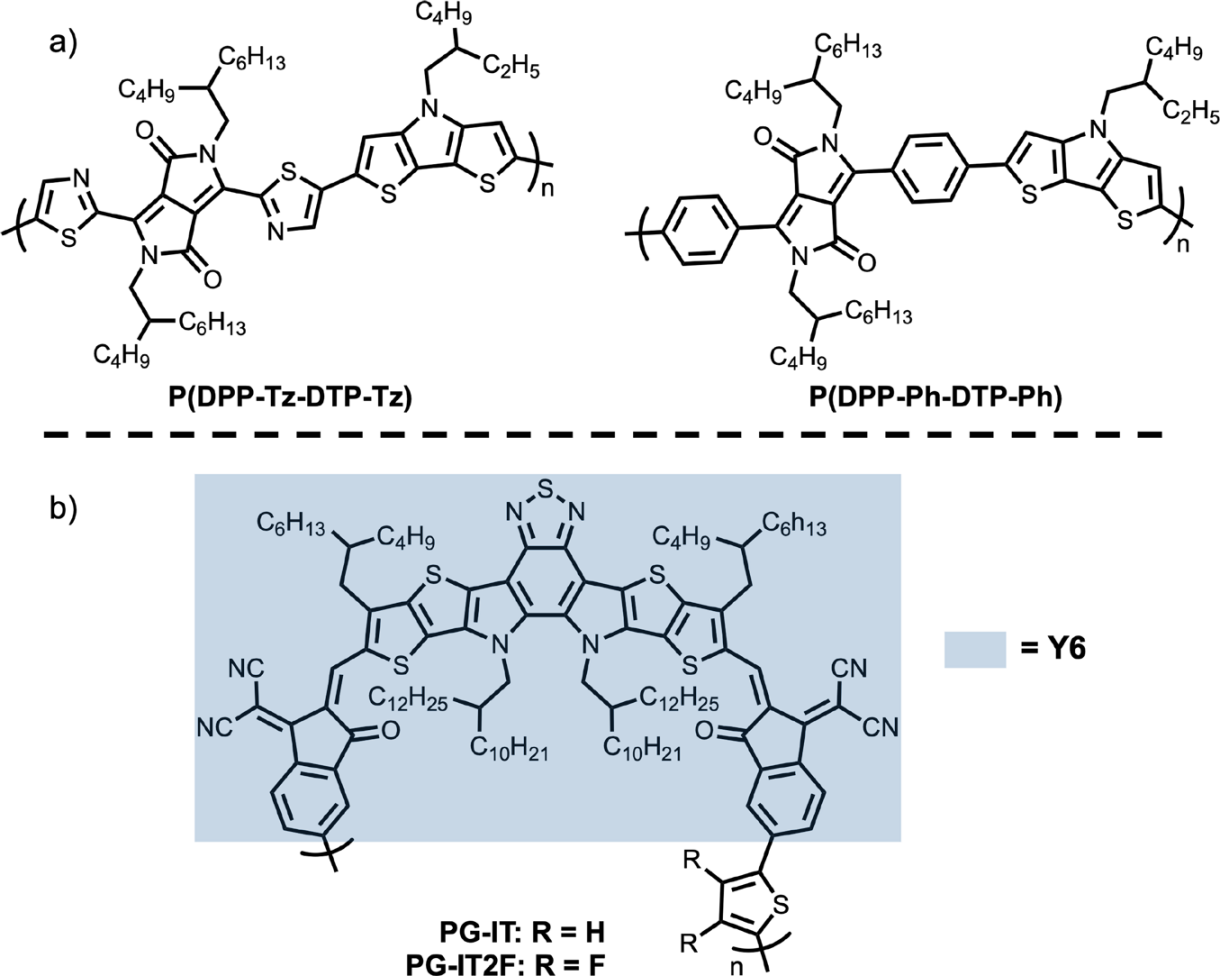
(a) Structures for two of the DPP copolymers with different π linkers.^83^ (b) Structures for two Y6 copolymers.^85^

The molecular dipole of a polymer, and its corresponding ability to generate electric fields, can also be optimized through the linkers between monomers. Willems et al. tested a library of 19 diketopyrrolopyrrole (DPP)-based copolymers with varying linkers between DPP units in bulk heterojunction solar cells.^82^ Using their novel DPP-based polymers as donor and phenyl-C_61_-butyric acid methyl ester (PCBM) as acceptor, they found a correlation between the electronic properties of the polymers, namely the oxidation potential, and the *V*_oc_ of the corresponding solar cells. The same research group later expanded this work to π-conjugated linkers in DPP-dithieno[3,2-b:2′,3′-d]pyrrole (DTP) copolymers. Interestingly, they reported that the nature of the linker influences the exciton binding energy, which ranged from 0.09 eV with a phenyl linker to 0.44 eV with a thiazole linker (Figure 8a).^83^ Although the authors did not explicitly refer to enhanced molecular dipoles, the reduction in exciton binding energy they observed when changing the linker is consistent with an increased compositional gradient across the distributed interface within the bulk heterojunction solar cell and therefore, an increased electric field supporting the separation of photogenerated charges.

**Figure 9:**
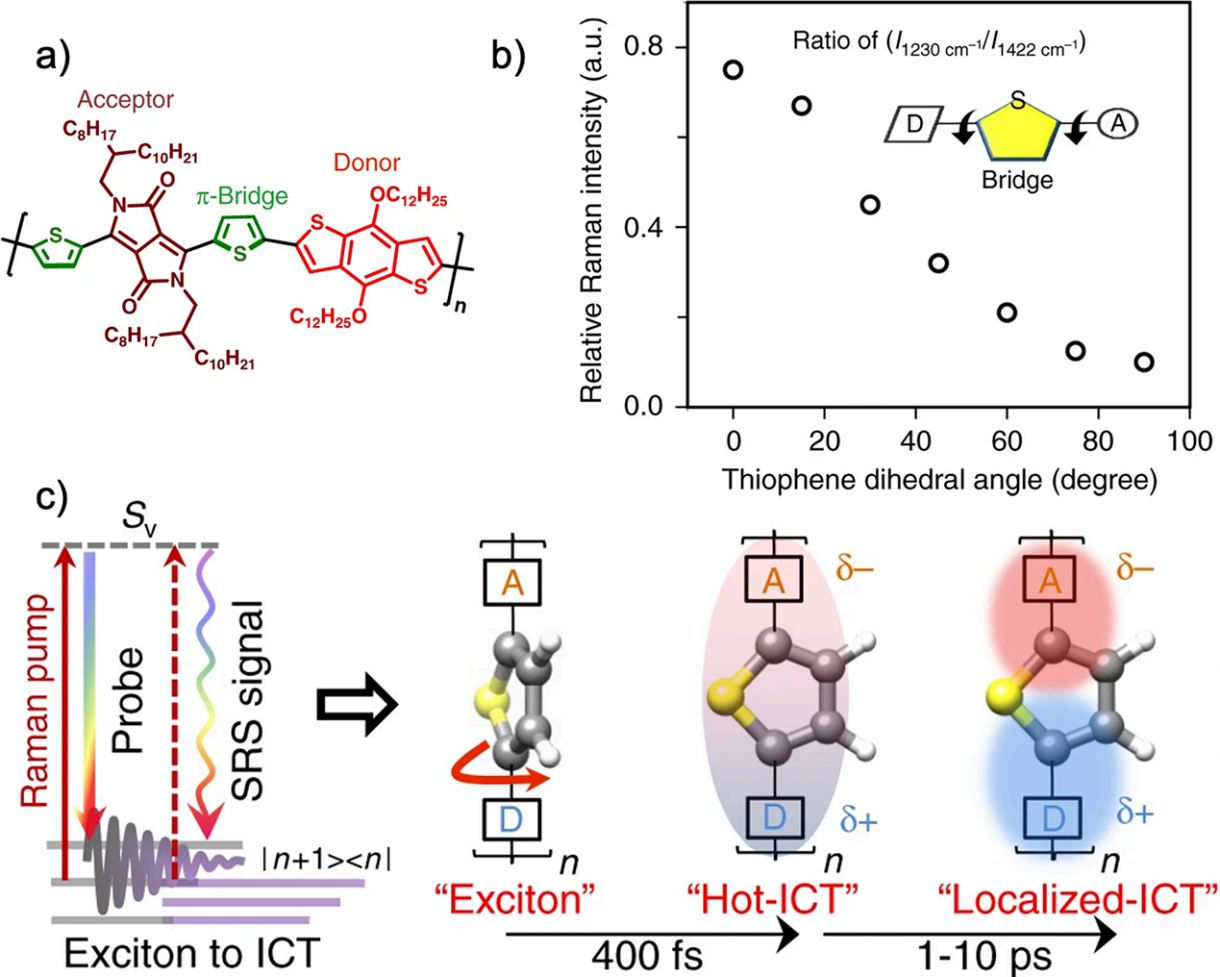
(a) The polymer structure investigated. (b) The ratio of the two tihophene Raman absorptions as a function of dihedral angle as predicted by DFT (B3LYP/6-31+G). (c) Scheme for the proposed intramolecular charge transfer mechanism. Reprinted (adapted) with permission from ref (84). Copyright 2017 Springer Nature.

Although it is recognized that a large change in dipole moment in donor-π-acceptor copolymers promotes charge carrier generation, the rational design of polymers with such electronic properties is not trivial.^84,105,106^ Roy et al. identified thiophene coplanarity as a primary structural factor to increase the dipole moment of poly(DPP-*co*-benzodithiophene), as shown in Figure 9a.^84^ Using femtosecond stimulated Raman spectroscopy, they showed that the absorbance band at 1228 cm^–1^, corrersponding to the thiophene C–H bending mode, and the one at 1422 cm^–1^, corresponding to the thiopohene C = C stretching mode, red-shifted by 5 and 4 cm^–1^, respectively, upon excitation. Using DFT, they were able to demonstrate that the relative intensity of the signal at 1228 cm^–1^ vs 1422 cm^–1^ correlates with changes in the thiophene bridge angle, as shown in Figure 9b. Their ultrafast spectroscopic data further supported that the π-bridge torsion angle influence the exciton dissociation rate through fine-tuning of the polymer dipole moment (Figure 9c).^84^ From this data, they suggest that designing π-bridges in conjugated polymers that quickly planarize upon excitation (ie. assisted through noncovalent interactions) will improve exciton dissociation and device efficiencies.

The optimization of π-bridge linkers was also pursued in the development of novel nonfullerene acceptors for organic solar cells. Indeed, independent groups have reported that π-bridge linker design for nonfullerene acceptors enables better exciton dissociation, charge extraction, and charge transport.^85,107,108^ To this end, several small molecule acceptors copolymerized with a π-bridge unit have been proposed.^85,109−112^ In particular, Yuan et al, reported polymers based on the small molecule acceptor (2,20-((2Z,20Z)-((12,13-bis(2-ethylhexyl)-3,9-diundecyl-12,13-dihydro-[1,2,5]thiadiazolo[3,4-e]thieno[2,”3”:4′,50]thieno[20,30:4,5] pyrrolo[3,2-g]thieno[20,30:4,5]thieno[3,2-*b*]indole-2,10-diyl)bis(methanylylidene))bis(5,6-difluoro-3-oxo-2,3-dihydro-1H-indene-2,1-diylidene))dimalononitrile) (Y6) using thiophene and difluorthiophene as linkers.^113^ Y6 derivatives, including polymerized small molecule acceptors with difluorene substituents on the thiophene π-bridge linker (Figure 8b) resulted in solar cells with 17.24% PCE, which was among the highest efficiencies reported for all-polymer solar cells in 2022.^85^ Alternatively, Fan et al. demonstrated that selenophene π – linkers yields solar cells with 15.1% PCE, compared to selenophene-free polymer acceptors with 13.0%.^86^ This group of studies reveals a strong correlation between the introduction of polar groups in π-bridge linkers, thereby modulating the dipole of the corresponding copolymer, and enhanced transport properties across electronic devices.

**Figure 10:**
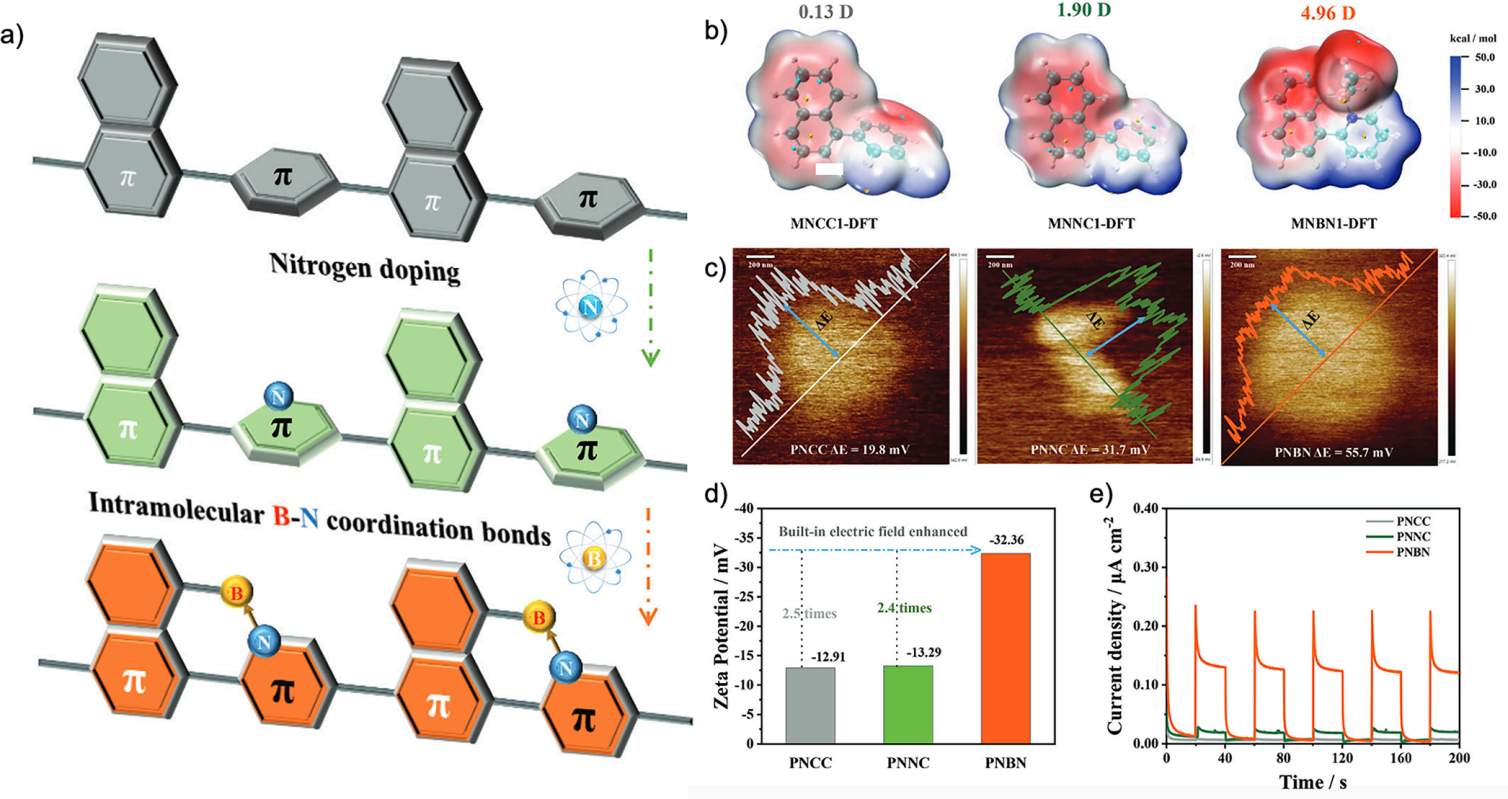
(a) Schematic of the three polymers investigated. (b) DFT (B3LYP/6-311G(d,p) calculated dipole moments and electrostatic potential maps for each of the corresponding monomers. Kelvin probe force microscopy (c), zeta potentials (d), and current density plots (e) of the three polymers.^81^ Reprinted (adapted) with permission from ref (81). Copyright 2022 John Wiley and Sons.

More recently, Ru et al. investigated how changing a simple phenyl-linker for a pyridyl-linker enhanced the built-in electric field in a conjugated polymer for photocatalytic hydrogen production.^81^ More specifically, they characterized the properties of phenyl-linked napthalene (MNCC1), pyridyl-linked napthalene (MNNC1), and pyridyl-linked aminonapthalene (MNBN1), as shown in Figure 10a. The molecular dipoles were predicted by DFT to be 0.13, 1.90, and 4.96 D, for the MNCC1, MNNC1, and MNBN1 monomers, respectively (Figure 10b).^81^ They rationalized the increased dipole moment by the presence of the relatively electron deficient pyridyl-linkers, an effect exacerbated by the presence of an amino-group through the planarization of the two rings. Kelvin probe force microscopy (KPFM) was used to measure the surface potential of the corresponding polymers: PNCC (19.8 mV), PNNC (31.7 mV), and PNBN (55.7 mV), as shown in Figure 10c. Meanwhile, their zeta potential was −12.91 mV, −13.29 mV, and −32.36 mV, respectively (Figure 10d). Both of these potentials are proportional to the magnitude of the built-in electric fields across the films, with PNBN having the greatest of the three polymers studied, consistent with its higher dipole moment. Correspondingly, they observed a photocurrent density for PNBN of 0.24 μA cm^–2^, which was 10 and 30 times greater than that of PNNC and PNCC, respectively (Figure 10e). This work builds upon existing literature that leverages B → N coordination to simultaneously planarize the backbone and modify the distribution of electron density,^114−117^ but specifically emphasizes the influence this has on the resulting electric field.^81^ Overall, their work highlights the importance of internal electric fields as a design principle for conjugated polymers.

**Figure 11:**
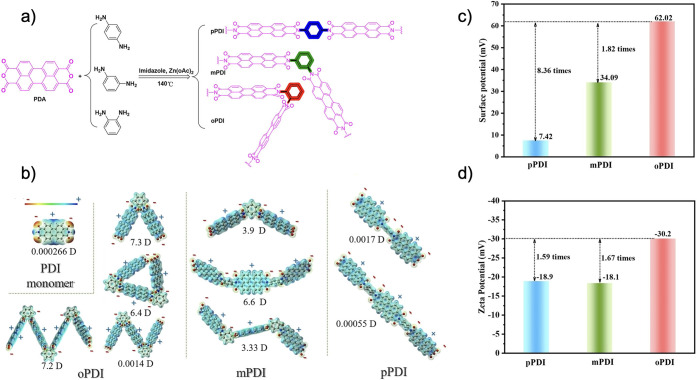
(a) Schematic representation of the o- m-, and pPDI polymer syntheses. (b) DFT (RB3LYP/6-31G(d,p)) predicted dipole moments of a PDI monomer, as well as dimers and trimers of the various PDI polymers. THe surface potential (c) and zeta potential (d) of the three PDI polymers. Reprinted (adapted) with permission from ref (33). Copyright 2023 John Wiley and Sons.

Linkers can also be used to intentionally decrease the planarity between symmetric monomers (i.e., with low dipole moment), which yields a more pronounced net dipole moment for the polymer. For example, Chen et al. recently investigated perylene diimide (PDI) polymers for hydrogen evolution.^33^ PDI polymers and other perylene-based polymers are commonly studied in photocatalysis,^24,33,62−64,66,91,118^ OLED,^61^ and other electronic device applications.^119^ PDI linkers such as urea^62^ and triazine^24^ have shown to increase the photo-oxidation rate of water and phenol, respectively, due to increased internal electric fields derived from improved crystallinity. In their work, Chen et al. used one of three simple phenyl-linker motifs to construct *ortho*- (oPDI), *meta*- (mPDI), or *para*- (pPDI) substituted poly(PDI), as shown in Figure 11a.^33^ They predicted the dipole moment of dimers with DFT. Starting from the PDI unit at 0.00026 D, they found increasing dipole moments of 0.0017, 3.9, and 7.3 D as the phenyl substitution goes from p-, to m-, to oPDI and the coplanarity decreases, as shown in Figure 11b.^33^ Similar trends were observed with trimers where the pPDI trimer exhibited a small dipole moment of 0.00055 D, owing to its planarity, and mPDI trimers were reported at 3.33 D (pseudotrans isomer) and 6.6 D (pseudocis isomer). Interestingly, the calculation of the dipole moment of oPDI trimer yielded 6.4 and 0.0014 D, depending on the configuration. However, the oPDI tetramer was predicted to have a dipole moment of 7.2 D, providing further insight on how to extrapolate these results from oligomers to polymer properties.^33^ They characterized the polymeric materials with KPFM, which showed a surface potential of 62.02 mV for oPDI. This was 1.82 and 8.36 times greater than that for mPDI and pPDI, respectively (Figure 11c).^33^ Zeta potential measurements followed a similar trend, with oPDI reported at −30.2 mV, which was 1.67 and 1.59 times greater than that of mPDI and pPDI, respectively (Figure 11d).^33^ Both the surface and zeta potentials generally agree with the predicted dimer dipole moments, highlighting the influence of the molecular level dipole on the polymer electric field.

**Figure 12:**
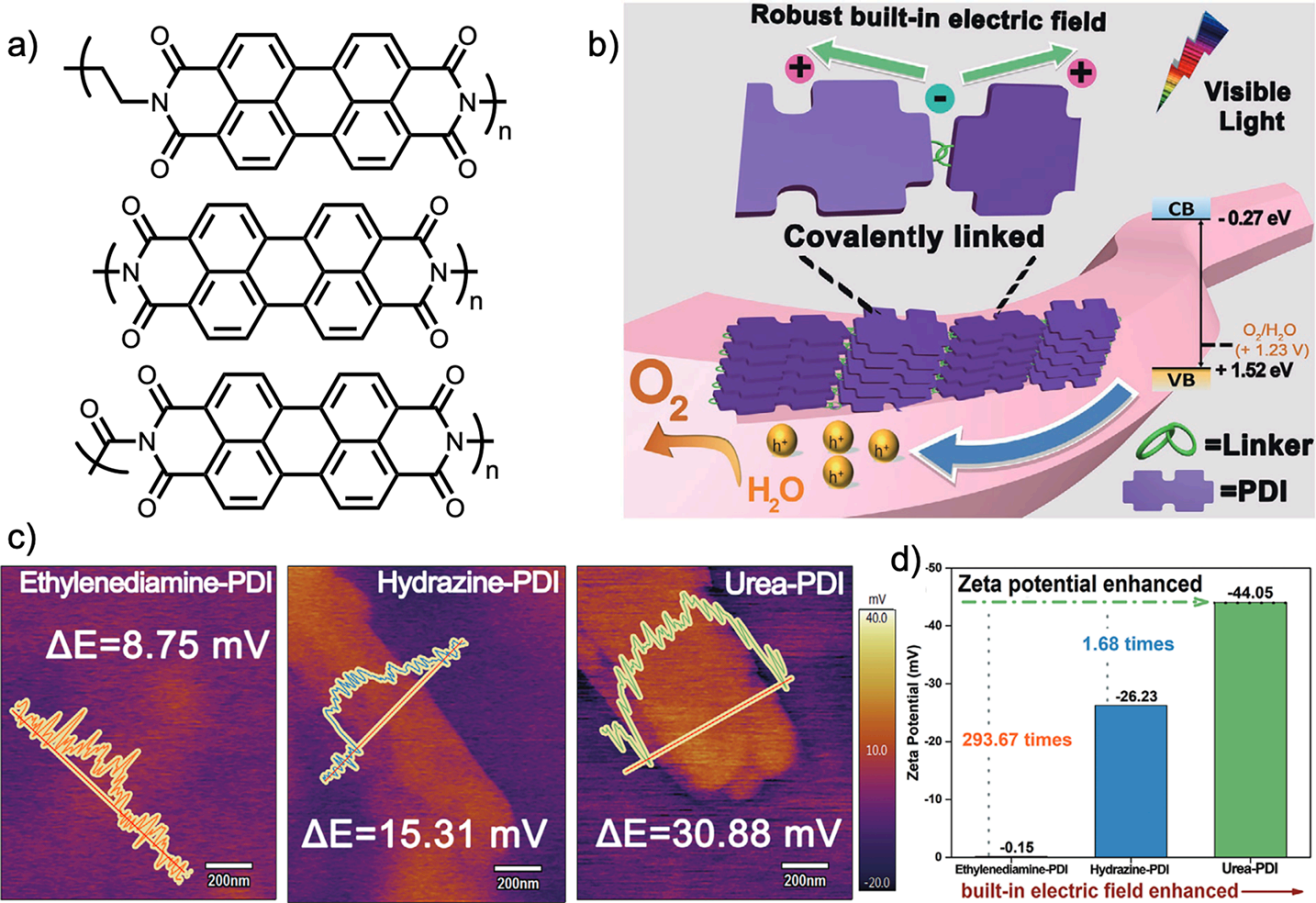
Structure of the three polymers (a) and a schematic of the impact of the BIEF effect (b). The surface potentials (c) and zeta potentials (d). Reprinted (adapted) with permission from ref (62). Copyright 2020 John Wiley and Sons.

Zhang et al. explored the influence of the nature of linkers between PDI units on their photocatalytic properties.^62^ As opposed to varied disubstituted phenyl-linkers, this synthesized an ethylene linker, a direct nitrogen-to-nitrogen PDI coupling, and a urea linker (Figures 12a and b), following previously reported protocols.^119,120^ This study built upon previous work using small molecule PDI systems, which assembled through noncovalent interactions,^121,122^ as well as a small molecule porphyrin system.^123^ They reported surface potential measurements of 8.75 mM, 15.31 mV, and 30.88 mV, respectively (Figure 12c). The same trend persisted for zeta potentials, which were reported at −0.15 mV, −26.23 mV, and −44.05 mV, respectively (Figure 12d). Therefore, each of these linkers contributed to an increased built-in electric field, which was purported to yield greater catalytic efficiency.^62^

Wang et al. also investigated how noncovalent tethers would impact similar PDI systems.^124^ They attached carboxylic acid pendents from the PDI units, which formed dimer-like hydrogen bonds between units. The PDI units were then able to π–π stack to form a supramolecular network. Zhu later used this design motif in conjunction with fullerene to enhance its photocatalytic activity 8.24 times compared to designs without the PDI species.^64^

### 3.3 Enhancing Electric Fields by Supramolecular Architecture Tuning

**Figure 13:**
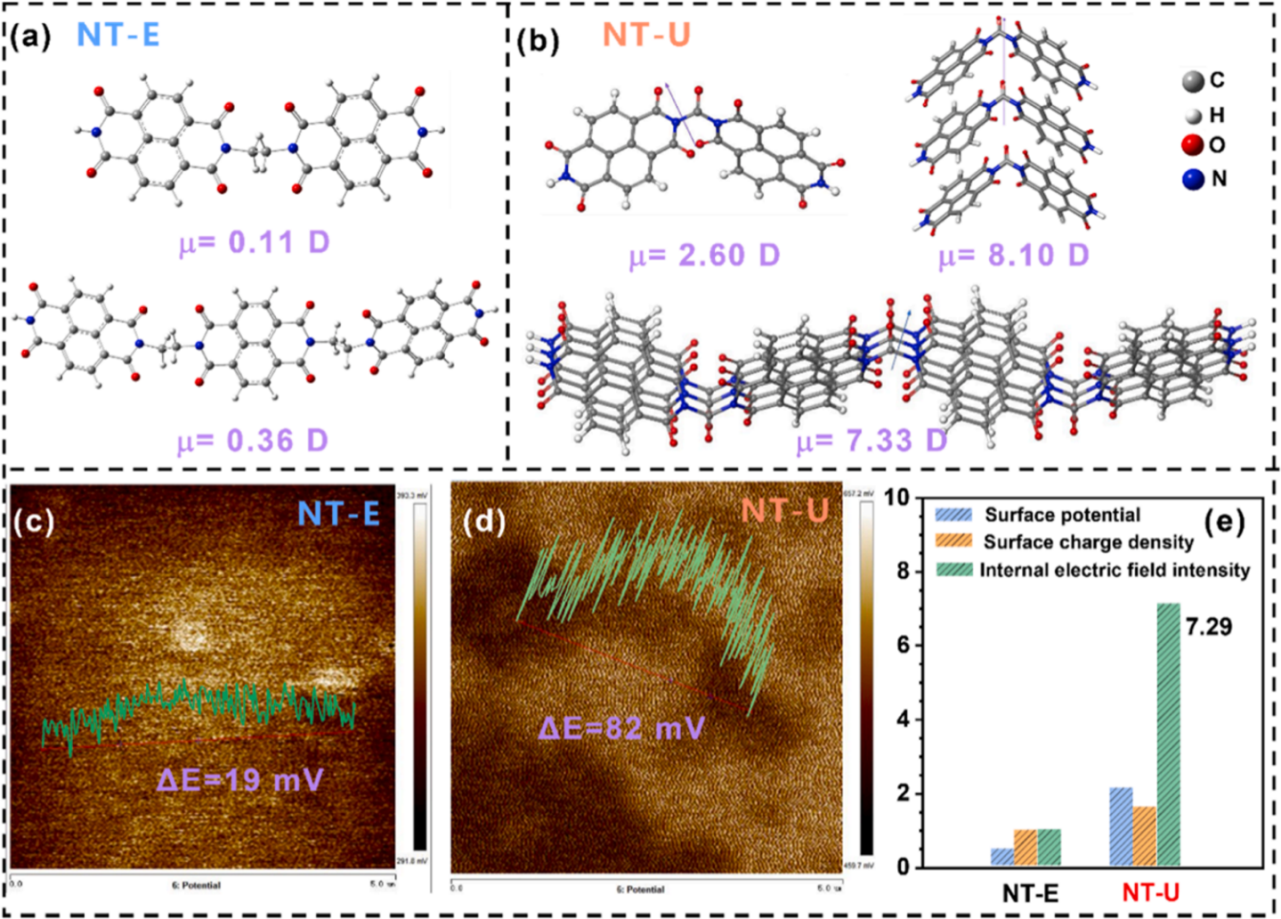
Molecular dipole of NT-E (a) and NT-U (b) with different layers and degrees of polymerization based on DFT calculations (B3LYP/6-311+G(d,p)). The surface potential of NT-E (c) and NT-U(d) measured using KPFM. (e) Analysis of the built-in electric field for each polymer. Reprinted (adapted) with permission from ref (39). Copyright 2024 Elsevier.

In the previous section, we discussed how linkers can influence polymer morphology and its impact on electric fields. This has largely been in the context of intramolecular interactions like backbone planarization that aligns the dipoles of the side chains. However, electric field effects in polymeric materials are also highly susceptible to intermolecular and interchain interactions such as π–π stacking. Indeed, Chen et al. demonstrated that layer-stacked polyimide had high crystallinity, which significantly enhanced built-in electric fields across the material in a lithium ion battery.^39^ They showed that these higher electric fields improved charge transport dynamics and electrochemical performance, highlighting the importance of morphology control. The linkers investigated in their work were an ethlyene- (NT-E) and a carbonyl-linker which generated a urea motif (NT-U) between each NDI unit.^39^ This motif is not unique,^62^ but their study brings built-in electric fields into focus. They predicted the molecular dipole of NT-E and NT-U dimers with DFT, yielding μ = 0.11 and 2.60 D, respectively (Figures 13a and b).^39^ The larger NT-U dipole moment compounds, where three dimers coordinated by a π–π stacking interaction exhibit a dipole of μ = 8.10 D, which is more than three times that of its components. Interestingly, the NT-U dimers are predicted to have a smaller dipole moment than oPDI discussed above,^33^ though the previously mentioned study did not investigate the influence of π–π stacking. The same effect for NT-E yields an increase from 0.11 to 0.64 D, which is more than five times greater than the components. However, the overall dipole magnitude of the NT-E trimer is still notably less than NT-U.^39^ X-ray diffraction data showed that NT-U was much more crystalline, which agrees well with the predicted dipolar alignment. They measured surface potentials with KPFM (Figures 13c and d) and found that NT-U exhibits a greater surface potential than NT-E. Coupled with zeta potential measurements, they found that NT-U generate an electric field 7.29 times greater than NT-E (Figure 13e).^39^ This increased electric field was then linked to the observed enhancement of charge transport in the same polymers when tested as a cathode in lithium ion batteries. NT-U showed greater initial theoretical discharge capacity of 60% (152/245 mAh*g*^–1^) compared to NT-E at 19% (31/163 mAh*g*^–1^). This example ties in the influence of linkages on the crystallinity of polymeric materials, achieved via π–π stacking, and their impact on built-in electric fields.

**Figure 14:**
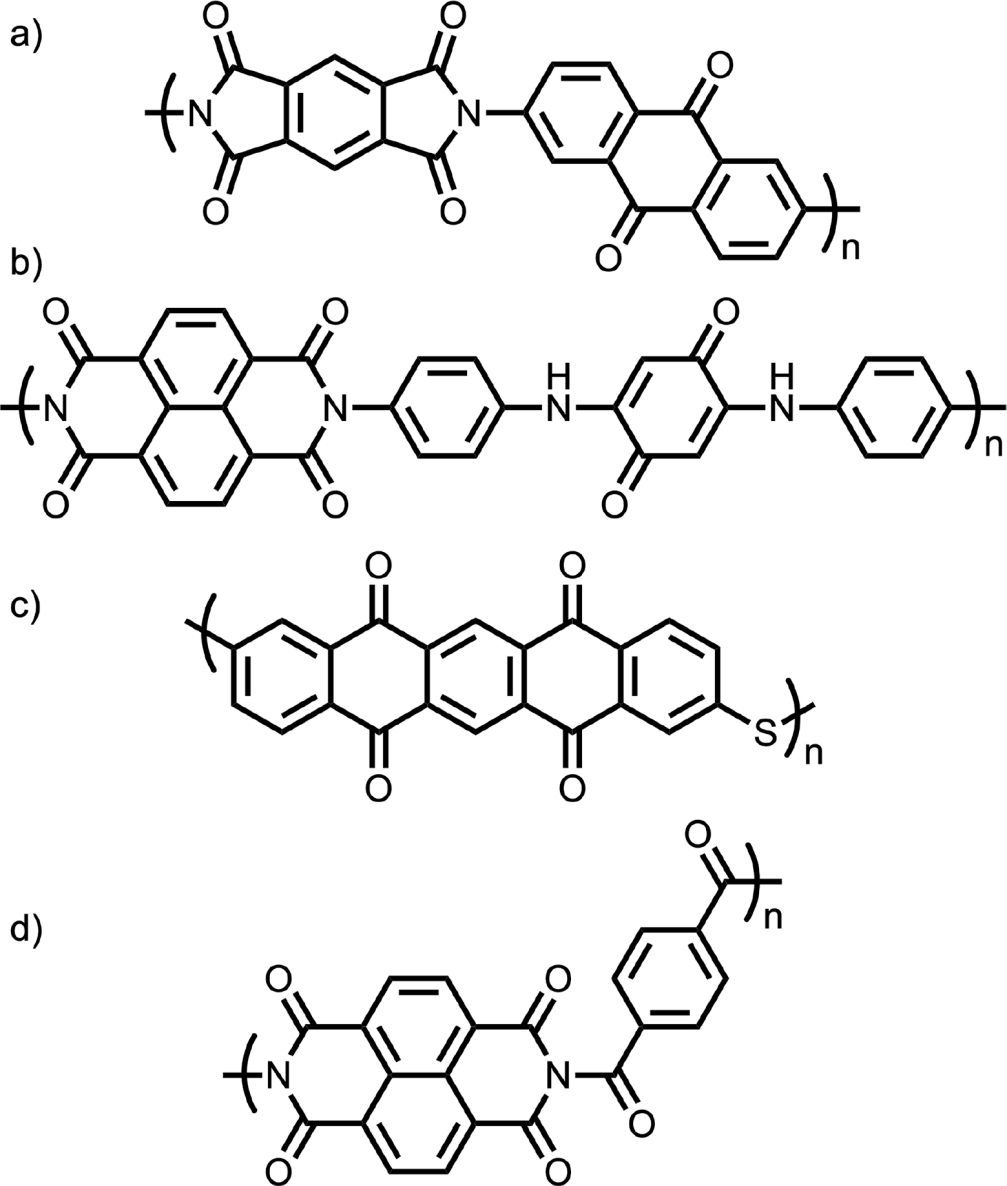
(a) Poly(pyromellitic diimide-co-anthraquinone),^87^ (b) benzoquinone-based copolymer,^88^ (c) poly(pentacenetetrone sulfide),^89^ and (d) poly(NDI-co-phenylene) with an amide linkage.^125^

Although they did not explicitly measure the corresponding built-in electric field, several other groups have investigated polymers with extended conjugation, yielding to higher crystallinity and enhanced electrochemical performance.^87−89,125^ Chen and Wang postulate that the higher crystallinity of polymeric materials is beneficial for cathodic materials in sodium or lithium ion batteries.^126^ Indeed, Pang et al. proposed conjugated porous polyimide poly(2,6-diaminoanthraquinone) benzamide (CP-PDAB), shown in Figure 14a, as an organic cathode material for sodium ion batteries.^87^ They demonstrated that the porous and loose structure of CP-PDAB enabled high cycling stability, achieving a specific capacity of 141 mAh g^–1^ at 500 mA g^–1^.^87^ Similarly, Ba et al. synthesized polyimide derivatives bearing anhydride with benzoquinone for lithium ion batteries (Figure 14b), realizing a reversible specific capacity of 145 mAh g^–1^ at 0.1 C.^88^ This performance enhancement was attributed to the micromorphology of the material after addition of the conjugated carbonyl groups from benzoquinone. Tang et al. reported the same result with poly(pentacenetetrone sulfide) (PPTS) (Figure 14c), which achieved a reversible specific capacity of 290 mAh g^–1^ at a current rate of 100 mA g^–1^.^89^ Importantly, these properties were largely retained after as many as 5000 charge–discharge cycles. Wang et al. further explored this idea with poly(NDI-co-phenylene) and an amide linkage between the two aromatic motifs (Figure 14d). This polymer also showed exceptional reversible capacity of 213.4 mAh g^–1^ at a current rate of 50 mA g^–1^. Interestingly, most of the polymers discussed above are predicted to have a small monomeric dipole moment.^87−89,126^

**Figure 15:**
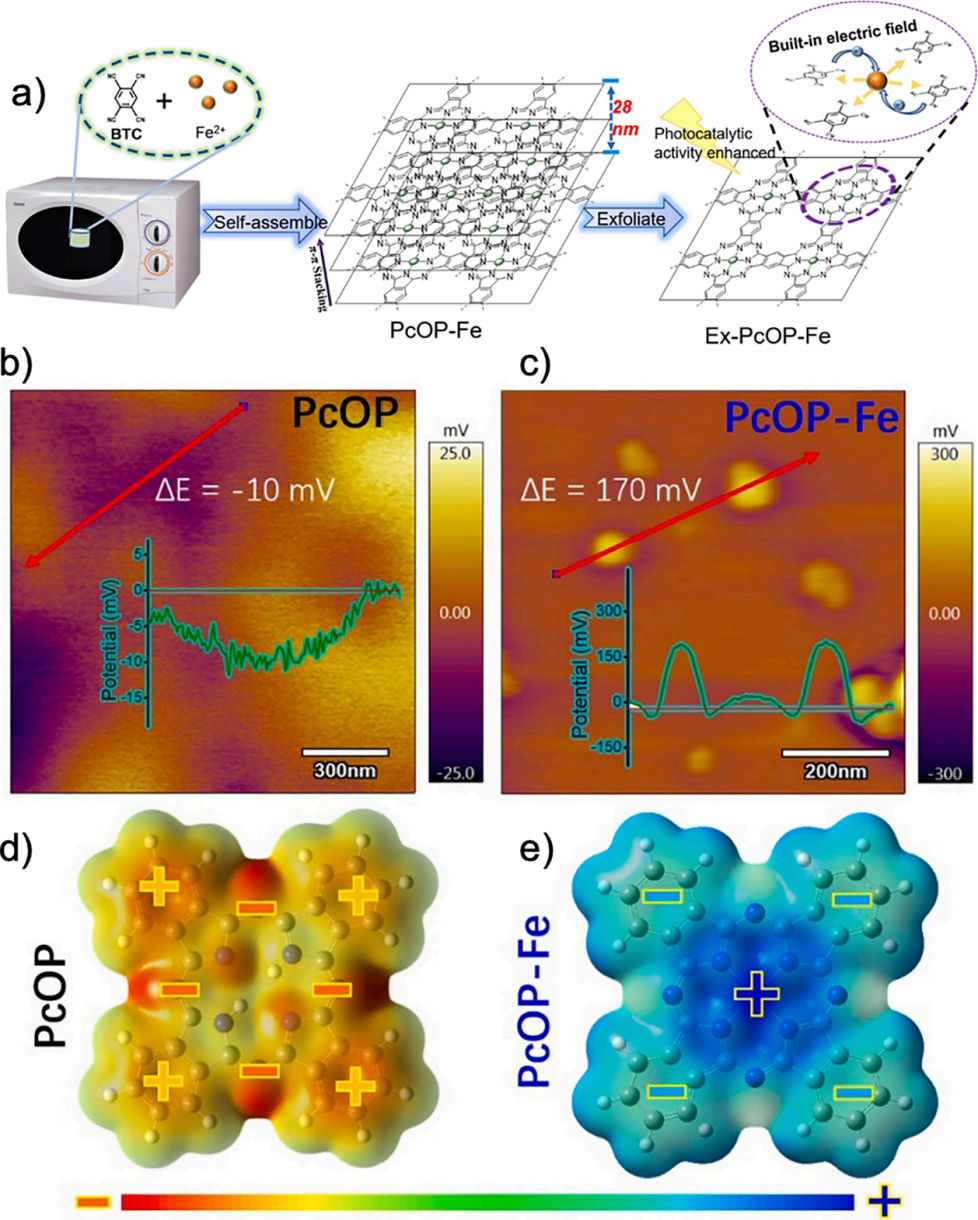
(a) Schematic representation of the FePc polymer synthesis. Surface voltage measurements for the iron-free (b) and iron-containing (c) Pc polymers. Surface electrostatic potentials for the iron-free (d) and iron-containing (e) Pc polymers, calculated by DFT (B3LYP/cc-PVTZ). Reprinted (adapted) with permission from ref (38). Copyright 2022 Elsevier.

In the context of photocatalysis and energy storage, many higher-dimensional conjugated materials are promising due to their inherently strong crystallinity and organization through π–π stacking. For example, 2D Phthalocyanine (Pc) polymers are an exciting material owing to their extended π-conjugation and rigid structure.^127,128^ Further, Pcs can be modified with various metal cations, which bind to the macrocyclic isoindoles, to tune their local electron density and alter their properties.^127−129^ Miao et al. recently studied Pc polymers and the impact of incorporating iron ions to modify its intrinsic electric field.^38^ The iron containing Pc polymer (PcOP-Fe) was prepared with a facile approach^130^ by combining benzene-1,2,4,5-tetraacbronitrile (BTC) with FeCl_3_ suspended in ethylene glycol and DBU, and allowed to react in a microwave reactor for three minutes (Figure 15a). A metal-free derivative (PcOP) was also prepared as a control. The surface voltages were measured to be −10 mV and 170 mV for PcOP and PcOP-Fe, respectively, as shown in Figures 15b and c. Further, zeta potentials were observed to be −0.40 mV and +0.87 mV, respectively. The greater magnitude of both surface and zeta potentials demonstrate that the presence of iron in the Pc macrocycle increases the built-in electric field in PcOP-Fe, compared to PcOP. PcOP is predicted to have high electron density on the nitrogen atoms linking each isoindole unit, with greater cationic character on the outerlying aromatic rings and at the center of the macrocycle (Figure 15c). For PcOP-Fe, the positive charge is concentrated in the center, with the outerlying benzene rings being relatively more electron-rich (Figure 15d). The more uniform charge distribution in PcOP-Fe is purported to reduce electronic dispersion and generate an electric field from the center to the perimeter, which ensures faster transfer kinetics when used as a photocatalyst.^38^

Jing et al. have also studied donor–acceptor type porphyrin complexes of Zinc tetraphenyl porphyrrin and tetrakis(4-hydroxyphenyl) porphyrin. The composite material generated an interfacial electric field that was 3.1 and 3.8 times greater than either of the respective components.^90^ Later, Yang et al. studied donor–acceptor type porphyrin-PDI complexes to a similar effect, generating an interfacial electric field that was 9.95 times greater than tetraphenyl porphyrin sulfonate, and 9.41 times greater than PDI alone.^74^ While these are small molecule systems, the enhanced internal electric fields may be promising when applied to polymers. Meanwhile, Gao et al. developed a PDI/BiOCl photocatalyst for the degredation of phenols.^91^ By using a weight ratio of 0.3 BiOCl:PDI, they found that PDI/BiOCl self-assembled with π–π stacking. Under full-spectrum light, PDI/BiOCl improved organic pollutant removal efficiency by 2.2 times compared to BiOCl and 1.6 times compared to PDI.^91^ PDI/BiOCl also had a photocurrent intensity of 4.17 μA cm^–2^ compared to 0.37 μA cm^–2^ for pure BiOCl and 0.76 μA cm^–2^ for PDI, showing that the donor-to-acceptor nature present in PDI/BiOCl benefitted the charge separation of photogenerated carriers.^91^

### 3.4 Opportunities for Electric Field Optimization

Supramolecular organization effects are relevant in the condensed phase as well as the solid-state. In earlier sections, we discussed how built-in electric fields often result from monomers with prominent dipoles and the way they organize. As such, it is reasonable to expect strong electric fields in supramolecular polymers, where monomer units arrange into a long chain due to short-range noncovalent interactions.^131^ However, electric fields in these systems are largely unexplored. In this section, we survey examples of supramolecular polymer architectures that leverage design principles (i.e., monomer dipole alignment, π–π stacking toward crystallinity) similar to those required for the generation of strong electric fields. This aims to highlight potential gaps in knowledge for future exploration in the field.

**Figure 16:**
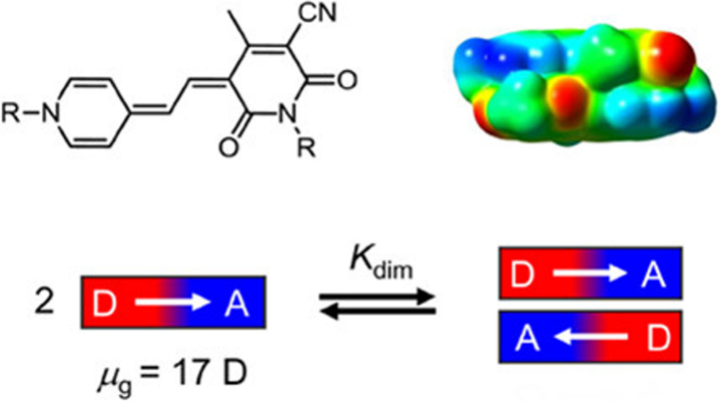
A depiction of the dipole–dipole driven dimerization of merocyanine molecules. Reprinted (adapted) with permission from Würthner, F. Dipole–dipole interaction driven self-assembly of merocyanine dyes: from dimers to nanoscale objects and supramolecular materials. *Acc. Chem. Res.***2016**, *49*, 868–876. Copyright 2016 American Chemical Society

**Figure 17:**
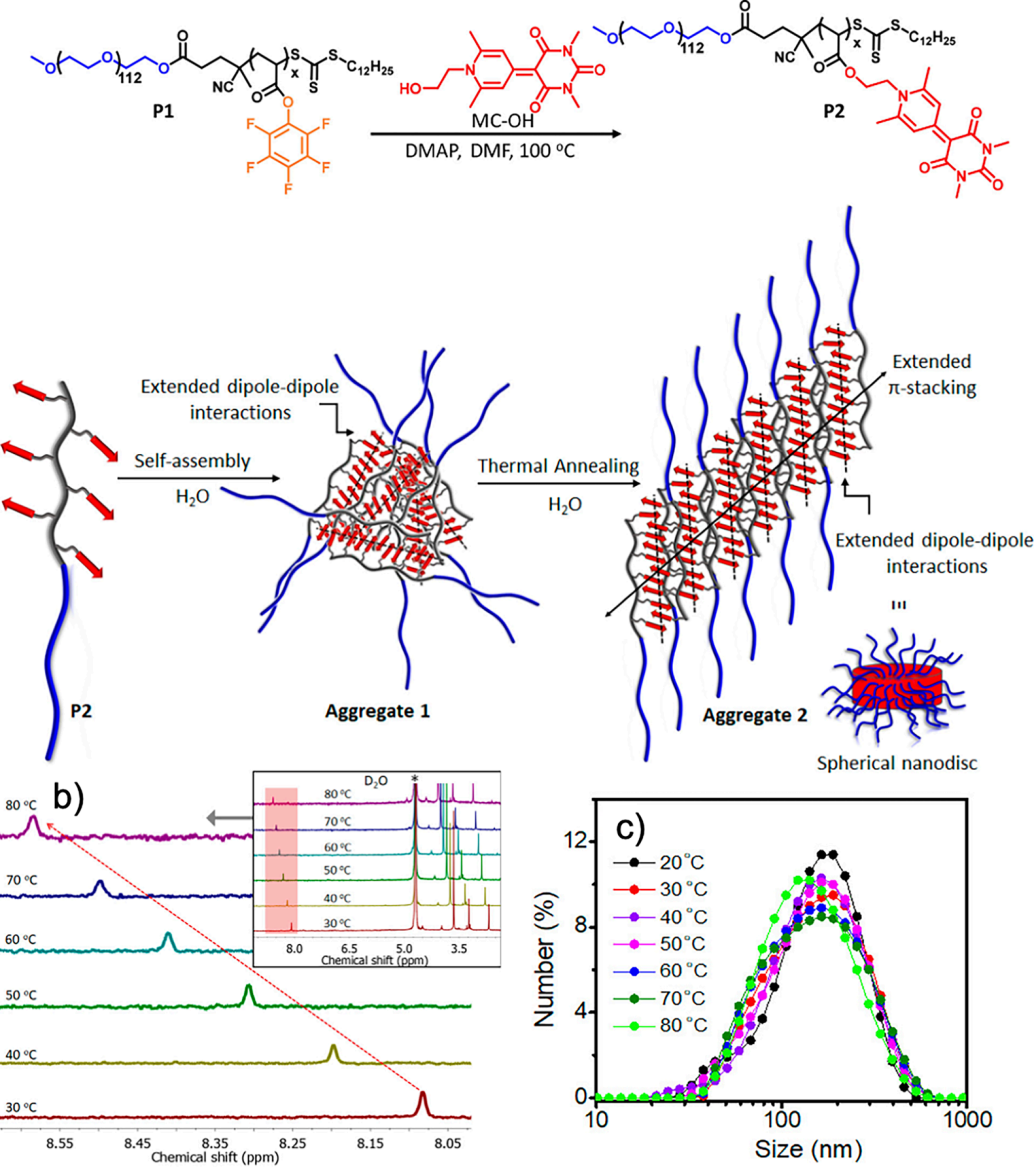
(a) Merocyanine containing block copolymer and its resulting aggregates. Variable temperature NMR (b) and DLS (c) analysis of aggregate 2. Reprinted (adapted) with permission from Rajak, A.; Das, A. Programmed macromolecular assembly by dipole–dipole interactions with aggregation-induced enhanced emission in aqueous medium. *ACS Polym. Au***2022**, *2*, 223–231. Copyright 2022 American Chemical Society.

Merocyanines are a common chemical motif in supramolecular polymers due to their large intrinsic dipole moments, often exceeding 7 D, that cause self-assemblly through dipole–dipole interactions (Figure 16).^132^ Merocyanine structures are diverse and highly tunable, making them ideal for use in an array of chemical environments.^133−136^ However, examples demonstrating long-range organization through these dipolar interactions are limited.^137−140^ Recently, Rajak and Das synthesized a block copolymer with a segment containing merocyanine pendents to leverage dipole–dipole interactions, achieving long-range order and organization (Figure 17a).^141^ They observe a broad distribution of polymer aggregates by dynamic light scattering (DLS), which increase in size and become more uniform upon thermal annealing in chloroform. Both variable temperature ^1^H NMR spectroscopy (Figure 17b) and DLS (Figure 17c) suggest that while small structural changes occur, the strong π–π stacking interactions between merocyanine pendents sustain the spherical aggregates at elevated temperatures. However, the introduction of a small amount of THF was able to disrupt the antiparallel merocyanine stacking.^141^ Meanwhile, Xu et al. developed a stimuli-responsive supramolecular polymer by appending urea to 2,3,2′,3′-tetrahydro-[1,1’]biindenylidene.^142^ The biinidenylene undergoes a cis–trans isomerization upon irradiation with 385 nm light, which is reversed with 365 nm light. The urea pendents intramolecularly hydrogen bond in the cis conformation, but intermolecularly hydrogen bond when isomerized to the trans conformation, yielding a gel.^142^ Although not explicitly mentioned, these supramolecular constructs are likely to enhance electric fields across the materials through enhanced crystallinity.

Korevaar et al. designed a dynamic supramolecular helical polymer, wherein the helicity is kinetically gated.^143^ Their *S*-chiral oligo(*p*-phenylenevinylene) (SOPV), first forms dimers through hydrogen bonding, followed by small disordered aggregates that propagate to form *M*- or *P*-helices, assembled through π–π stacking interactions of the aromatic core. *M*-helices can be converted to *P*-helices upon addition of S-chiral dibenzoyl tartaric acid with gentle heating, and subsequently cooling to 0 °C. The opposite transformation can be achieved through heating *P*-helices above 25 °C.^143^ Kulkarni et al. investigated dynamic helicity as well through solvent clathrate interactions.^144^ The cornonene bisimides assemble through π–π stacking as well. Without chiral substituents, the supramolecular polymer only formed a helix in the presence of *n*-heptane, which was shown to bind in between the alkyl pendents. This effect was temperature dependent, forming *P*-helices at (relatively) higher temperatures (−5 °C) and *M*-helices at lower temperatures (below −30 °C).^144^

**Figure 18:**
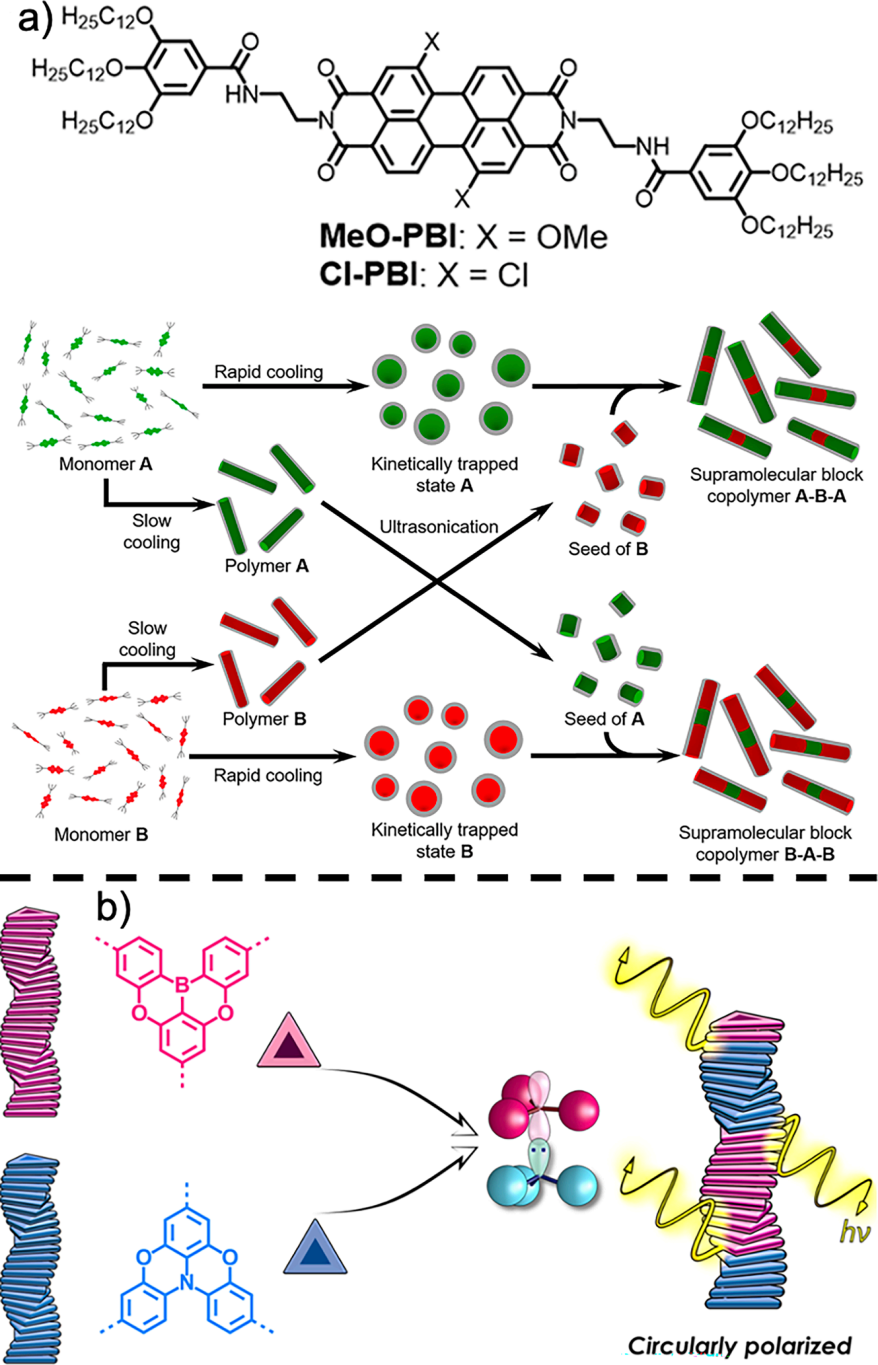
(a) NDI-based supramolecular block copolymers with different subtituents to tune their electronic structure, where the green structures are methoxy-substitued and the red block are chloro-substituted. Reprinted (adapted) with permission from Wagner, W.; Wehner, M; Stepanenko, V.; Würthner, F. Supramolecular block aopolymers by seeded living polymerization of perylene bisimides. *J. Am. Chem. Soc.***2019**, *141*, 12044–12054. Copyright 2019 American Chemical Society. (b) B–N interaction based supramolecular block copolymers. Reprinted (adapted) with permission from Adelizzi, B.; Chidchob, R.; Tanaka, N.; Lamers, B.A.G.; Meskers, S.C.J.; Ogi, S.; Palmans, A.R.A.; Yamaguchi, S.; Meijer, E.W. Long-lived charge-transfer state from B–N frustrated lewis pairs enchained in supramolecular copolymers. *J. Am. Chem. Soc.***2020**, *142*, 16681–16689. Copyright 2020 American Chemical Society.

Supramolecular block copolymers can also be formed, as demonstrated by Wagner et al.^145^ This was achieved by leveraging electron-rich and electron-deficient PDIs to favor π–π stacking between them (Figure 18a). Similarly, Adelizzi et al. designed supramolecular block copolymers with boron and nitrogen cores (Figure 18b).^146^ These supramolecular copolymers exhibited interesting optical properties. However, considering the electric field improvements reported in Section 3.2, these constructs could also yield higher electric fields owing to increased order afforded by favorable B ← N interactions, as well as the resultant B–N bond dipole. Liao et al. investigated alternating supramolecular copolymers, highlighting the versatility of accessible supramolecular assemblies.^147^ They achieved this by using anthracene and tetracene based π-cores with *R*- and *S*- chiral substituents respectively, forming through a combination of π–π stacking interactions and steric clash to drive the alternating supramolecular polymer.

Despite the reliance on dipole or π−π stacking interactions in the structure of supramolecular polymer networks, they are seldom studied in the context of electric fields. Future work leveraging these effects may be beneficial in improving the performance of devices using, identifying new applications for, or aiding in the synthesis of supramolecular polymers.

## 4 Novel Polymer Classes to Enhance Electric Fields

In this section, we review emerging ideas to enhance electric fields in polymeric systems, including the incorporation of polyelectrolytes, ferroelectric, piezoelectric or polaron-conducive polymers. Although not directly designed to enhance electric fields, these innovative materials have the potential to make significant advances in that space. The papers reviewed here provide a starting point for future work focused on optimizing electric fields in polymeric materials.

### 4.1 Nonconjugated Polyelectrolytes

**Figure 19:**
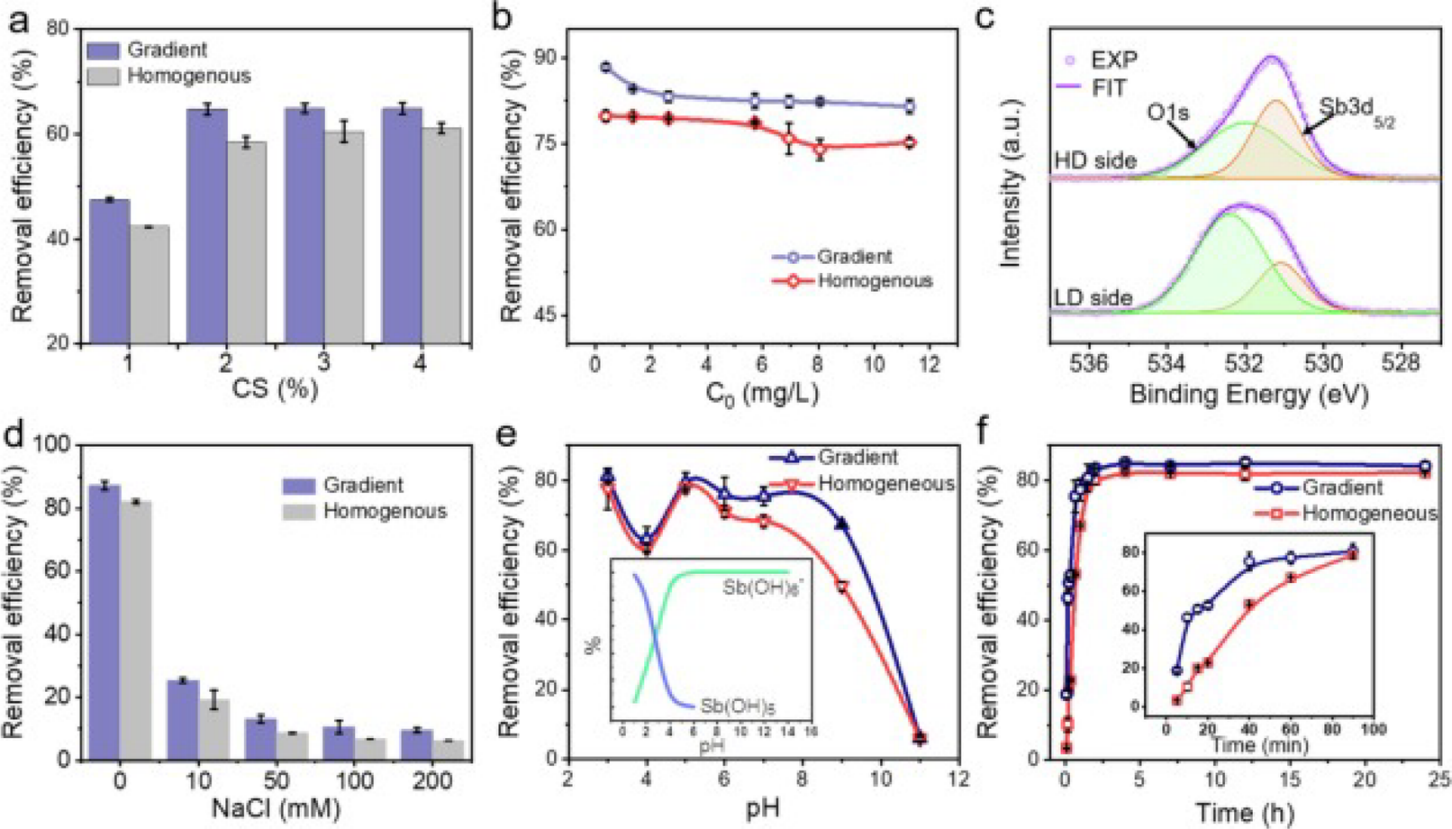
Comparisons of anionic heavy metal removal performance by gradient and homogeneous hydrogels, and Sb(V) was selected as model pollutant. (a) Effect of chitosan (CS) concentrations. (b) Effect of initial Sb(V) concentrations. (c) O 1 s and Sb 3d XPS spectra of gradient hydrogel after eliminating of Sb(V) where HD is higher (network) density, and LD is lower density. (d) Effect of ionic strength. (e) Effect of pH values. (f) Removal kinetics. Reproduced with permission from ref (148). Copyright 2022 Elsevier.

In Section 3, we discussed how conjugated polyelectrolytes could make efficient interlayers in electronic devices, boosting device performance by enhancing built-in electric fields. Here, we discuss nonconjugated polyelectrolytes in a solid state application and beyond, as they are also natural candidates for electric field analyses due to their inherent charges. For example, Qi et al. demonstrated that utilizing chitosan as a polyelectrolyte hydrogel boosted heavy metal removal through interfacial and built-in electric fields.^148^ The electric fields, generated by the counterions, allowed for diffusion and rapid removal of heavy metal pollutants. They found that gradient hydrogels prepared with acrylamide, protonated chitosan, and UiO-66 MOFs improved performance compared to traditional hydrogels containing acrylamide, neutral chitosan, and UiO-66 MOFs (Figure 19).^148^ However, poor adsorption of heavy metals was observed in high ionic strength media due to Debye screening. They addressed this issue in a follow-up study, opting for further optimization through the tuning electrostatic interactions.^149^ They fabricated a salt-tolerant gradient hydrogel, poly[(3-acrylamidopropyl)trimethylammonium chloride-benzyl acrylate] (P(APTC-BZA)), whose built-in potential maintained around 130 mV at NaCl concentrations up to 700 mM. The removal efficiency of Sb(V) only decreased from 69% at 0 mM NaCl, to 55% at 500 mM NaCl, with an initial concentration of 0.5 mg L^–1^ of Sb(V). In comparison, hydrogels made with gradient salt-intolerant P(APTC) decreased from 38% at 0 mM NaCl, to 10% at 500 mM NaCl, in the same environment. This study emphasizes the importance of not only enhancing electric fields in polymers, but also the importance of maintaining these fields in a variety of environments.

**Figure 20:**
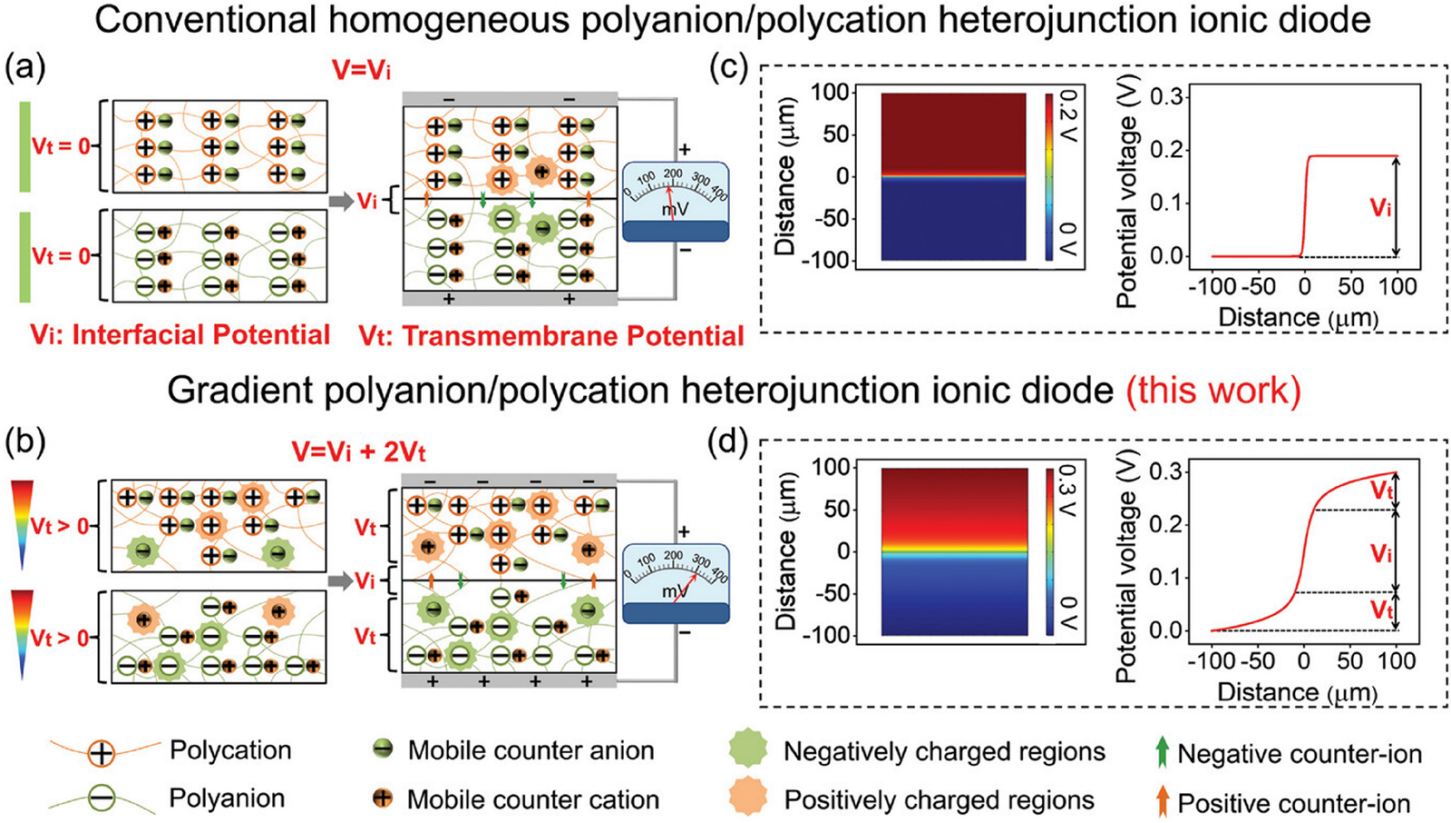
Schematic depiction of (a) conventional ionic diode, (b) gradient ionic diode, (c) simultated potential profile within conventional ionic diode and (d) gradient ionic diode. Reproduced with permission from ref (150). Copyright 2024 John Wiley and Sons.

Wang et al. developed gradient p-polyanion/n-polycation heterojunction hydrogels with built-in electric fields in regards to self-powered iontronic devices.^150^ The gradient hydrogels were created from the same materials used to synthesize conventional homogeneous hydrogels, poly(acrylamide-2-acrylamide-2-methylpropane sulfonic acid) (P(AM-*co*-AMPS)) and poly(acrylamide-2-methylpropane acryloyloxyethyl trimetyl ammonium chloride (P(AM-*co*-ATAC)). The gradient was prepared by applying an external electric field to assist free radical polymerization and to allow migration of the AMPS toward the anode in the polycation gel to create gradient P(AM-*co*-AMPS) and ATAC toward the cathode in the polyanion gel to create gradient P(AM-*co*-ATAC). They combined the two gels at the low density sides of each gradient to create their overall hydrogel (Figure 20). When comparing the gradient ionic diode-based device with devices using the conventional homogeneous hydrogels, the output voltage increased from 4.1–135 mV in homogeneous hydrogels, to 306.35 mV in their gradient hydrogel device. Power density and pressure-perception sensitivity of their devices also increased by 162.76% and 128.71% (to 50.16 μW cm^–2^ or 250.8 μW cm^–3^ and 43582 mV MPa^–1^), respectively.

**Figure 21:**
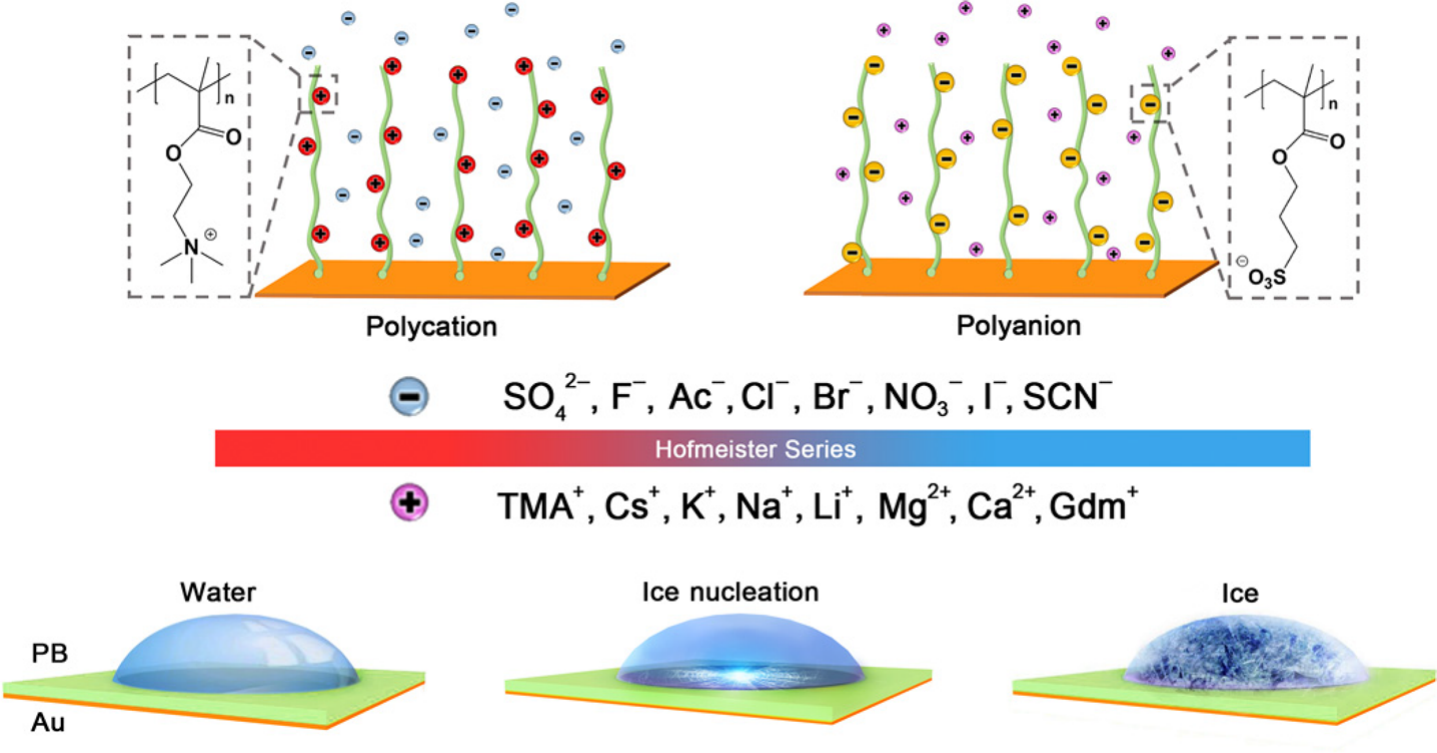
Illustration of HIN on cationic and anionic PB surfaces with different counterions. The counterions on the PMETA and PSPMA brush surfaces can be successfully exchanged by immersing the brush surface into a solution containing expected counterions. Reproduced with permission from ref (151). Copyright 2016 The American Association for the Advancement of Science.

Polyelectrolytes also exhibit utility in altering the dynamics of water at interfaces. He et al. studied the effects of diffused counterions on heterogeneous ice nucleation (HIN) using polyelectrolyte brushes (PB).^151^ By soaking the PB surface in a solution of anionic or cationic counterions, the PB could be reversibly converted to the cationic poly[2-(methacryloyloxy)-ethyltrimethylammonium] (PMETA) or anionic poly(3-sulfopropyl methacrylate) (PSPMA), respectively (Figure 21). They found that the HIN temperature trend with anionic counterions (SO_4_^2–^ < F^–^ < Ac^–^ < HPO_4_^2–^ < Cl^–^ < Br^–^ < SCN^–^ < NO^3–^ < I^–^) nearly followed the Hofmeister series when using PMETA as the PBs. For example, when switching the counterions from I^–^ to SO_4_^2–^, the HIN temperature dropped from −23.8 °C to −26.4 °C. To investigate this trend further, they performed MD simulations with three representative anions, F^–^, Cl^–^, and I^–^. Although the electric fields at the brush/water interface induced by the counterions (with magnitudes of 100–1000 kV cm^–1^) matched the trend of HIN temperatures, the magnitudes were deemed not great enough to significantly impact HIN as the required electric field magnitude to induce ice nucleation is on the order of 10000 kV cm^–1^. Instead, electric fields played an indirect role with stronger electric fields generated by thicker PBs imposing higher attractive potential on the counterions, preventing them from escaping into the brush/water interface. This effect is more pronounced in weakly hydrated ions like I^–^.^151^ With the subsequent increase in anion charge density, the orientation relaxation of water molecules at the interface decays more slowly with PMETA-I and PMETA-Cl having 8% and 6% more ice-like water molecules than PMETA-F.^151^ The investigation of polyelectrolyte effects on HIN by He et al. exemplifies analysis of the interplay between electric fields and another property, ion density in this case, that offers insight on an elusive phenomenon.

**Figure 22:**
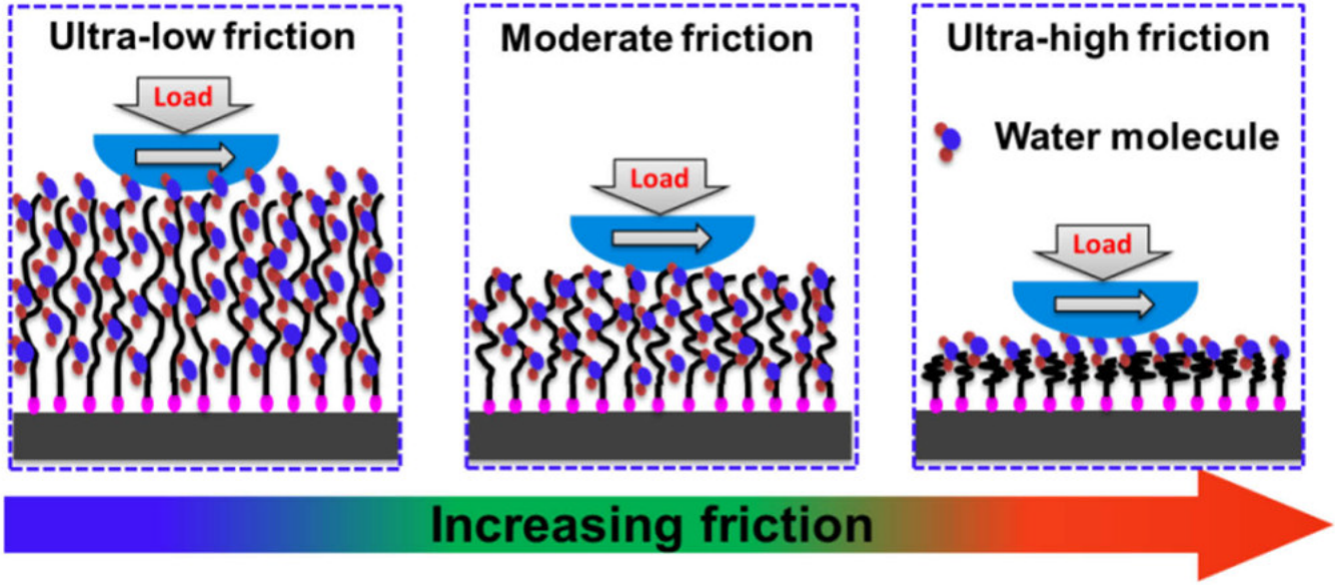
Illustration showing the conformational changes in the PBs induced by counterions. Reproduced with permission from Wei, Q.; Cai, M.; Zhou, F.; Liu, W. Dramatically tuning friction using responsive polyelectrolyte brushes. *Macromolecules***2013**, *46*, 9368–9379. Copyright 2013 American Chemical Society.

Wei et al. studied tuning friction using PBs that experience conformational changes in response to counterions (Figure 22).^152^ Friction coefficients increased from orders of 10^–3^ to 10° depending on the counterion.^152^ The PBs contained charged groups that generated internal electric fields, influencing interactions between the PBs and the counterions. The internal electric fields also modulated the distribution and orientation of water molecules within the brush, further contributing to the conformational changes in the PBs. The size, hydration, and polarizability of the counterions determined the strength of the ion-pairing interactions where hydrophobic counterions, such as TFSI^–^, weakened the internal electric fields through formation of stronger ion pairs. This led to a collapse of the polymer chains and an increase in the friction coefficient. Hydrated ions, such as Cl^–^, contrastingly allowed the PBs to remain extended and hydrated, allowing for low friction coefficients. This study provides an example of how electric field tuning influences polyelectrolyte properties and structure to create materials with tailored characteristics.

### 4.2 Ferroelectric Polymer Additives

Ferroelectric polymers, which have been polarized through cosolvent recrystallization without a poling process, have been studied by Kumari et al. for their ability to increase halogen-free organic solar cell efficiency through induced built-in electric fields.^10^ Ferroelectric materials feature spontaneous polarization that can be switched by applying an external electric field.^10,153,154^ They found that through the induction of polarization by polyvinylidene difluoride (PVDF)-based ferroelectric additives in *o*-xylene/*N*-methylpyrrolidone (NMP), high efficiencies were attained, 11.02% and 11.76% for fullerene (PTB7-Th:PC71BM) and nonfullerene (PM6:IT-4F) bulk-heterojunction solar cells, respectively.^10^ They also extended the use of ferroelectric additives to a p-n-like bilayer solar cell, demonstrating an efficiency of 11.83%.^10^ In the process of ferroelectric polarization, Kumari et al. saw enhancements of photovoltaic properties such as short-circuit current density (*J*_sc_) and fill factor (FF) as summarized in Table 2. The ferroelectric additives improved PCE with the exception of P1 where using NMP alone exhibited better performance. The worsening in the performance was attributed to aggressively large aggregation in OSCs with P1 added compared to the other OSCs.

**Table 2:** Summary of Device Parameters for Bulk-Heterojunction (BHJ) OSCs with and without PVDF-Based Ferroelectric Additives under AM 1.5G Irradiation at 100 mW cm^–2^^a^

Additive	Concentration	*J*_sc_ (mA cm^–2^)	*V*_oc_ (V)	FF (%)	PCE (%)
As-cast^b^	–	11.84 ± 0.68	0.790 ± 0.005	45.3 ± 1.5	4.23 ± 0.44 (4.67)
NMP only^b^	3.0 vol %	19.13 ± 0.30	0.796 ± 0.003	65.4 ± 0.3	9.96 ± 0.24 (10.20)
**P1**^b^	1.5 wt %	19.83 ± 0.36	0.803 ± 0.001	61.5 ± 0.7	9.79 ± 0.31 (10.10)
**P2**^b^	1.5 wt %	20.01 ± 0.29	0.810 ± 0.002	66.1 ± 0.4	10.72 ± 0.30 (11.02)
**P3**^b^	2.0 wt %	19.75 ± 0.32	0.794 ± 0.001	65.0 ± 0.8	10.20 ± 0.30 (10.50)
**P4**^b^	1.5 wt %	19.78 ± 0.31	0.805 ± 0.002	66.4 ± 0.4	10.58 ± 0.25 (10.83)
As-cast^c^	–	17.01 ± 0.15	0.829 ± 0.004	62.1 ± 1.2	8.84 ± 0.20 (9.04)
**P2**^c^	1.0 wt %	18.94 ± 0.15	0.829 ± 0.002	73.9 ± 0.3	11.61 ± 0.15 (11.76)

a Ferroelectric additives are marked in bold. Data corresponds to the average value of 10 devices and deviation from its maximum; data in parentheses corresponds to the maximum values.

b PTB7-Th:PC71BM based OSC devices.

c PM6:IT-4F based OSC devices. Adapted with permission from ref (10). Copyright 2020 Elsevier.

**Figure 23:**
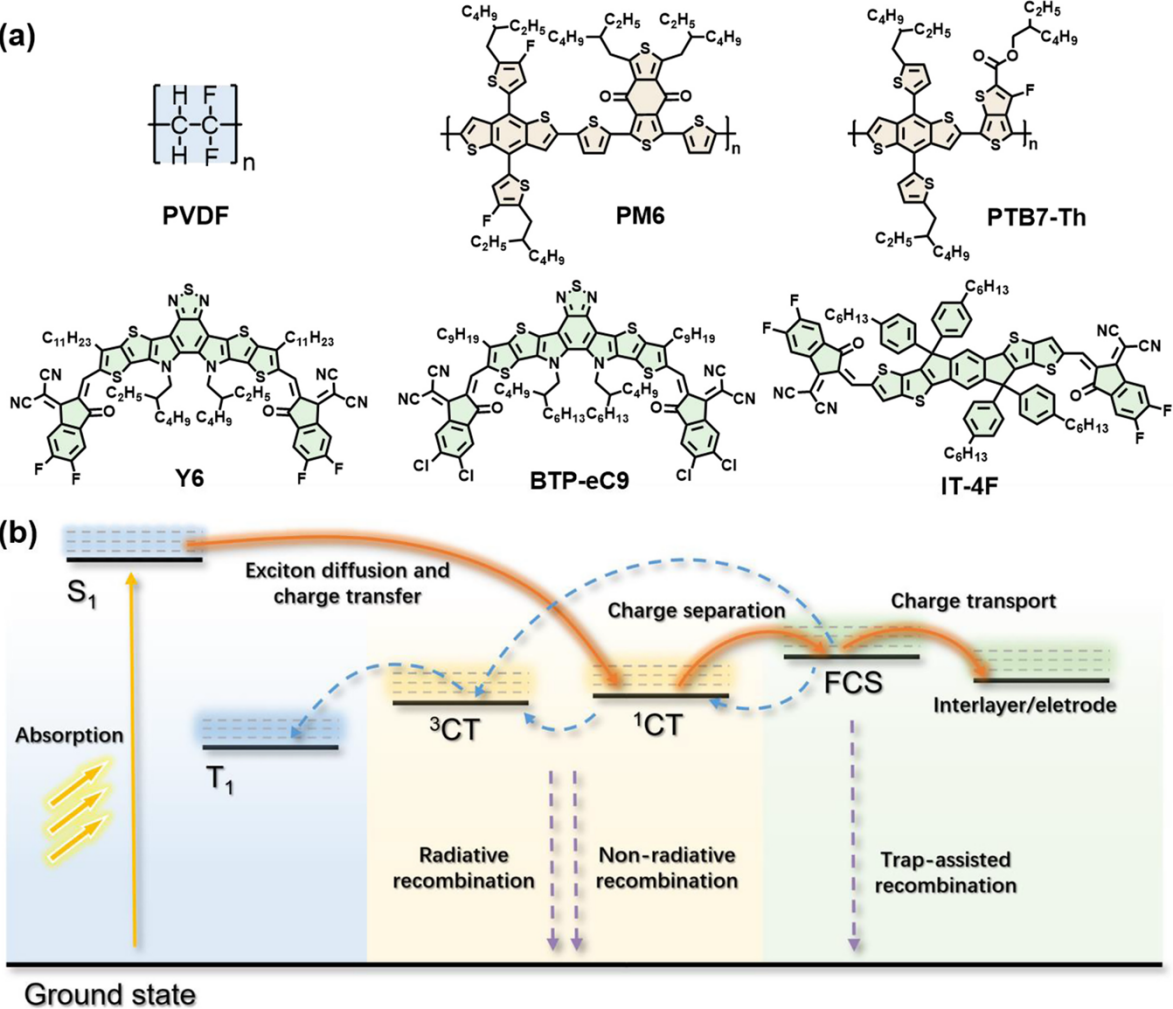
Scheme showing (a) the polymer structures and (b) energy transfer processes where the orange solid lines represent acceleration, and the blue dashed lines represent inhibition. Adapted with permission from ref (155). Copyright 2022 John Wiley and Sons.

Yuan et al. also exploited the anomalous photovoltaic effect with ferroelectric polymers, enhancing efficiency in organic solar cells.^69^ More specifically, they showed that inserting an ultrathin ferroelectric layer in the cell decreased the recombination in the CTE through an augmented electric field, thus increasing the *V*_oc_.^69^ Deng et al. also saw enhanced built-in electric field through the addition of PVDF as a ferroelectric additive into nonfullerene solar cells.^153,155^ They tested various different active layers such as PM6:Y6, PM6:BTP-eC9, PM6:IT-4F and PTB7-Th:Y6, resulting in an efficiency of 17.72% for a PM6:Y6-based organic solar cell and 18.17% for a PM6:BTP-eC9-based cell, as summarized in Table 3.^155^ The added electric field promoted exciton separation, charge transport, and improved film morphology, with a mechanistic overview shown in Figure 23.

**Table 3:** Photovoltaic Performance of the Different Active Layers OSCs with PVDF Additive Treatment under AM 1.5 G Illumination at 100 mW cm^–2^

Active layers	PVDF^a^	*V*_oc_ (V)	*J*_sc_ (mA cm^–2^)	*J*_sc_^cal^ (mA cm^–2^)	FF (%)	PCE (%)
PM6:Y6	–	0.852	25.62	24.87	73.43	15.92 ± 0.08 (16.03)
	1 wt %	0.860	25.96	25.22	75.86	16.78 ± 0.12 (16.95)
	5 wt %	0.862	26.81	26.10	76.66	17.59 ± 0.11 (17.72)
	10 wt %	0.862	26.19	25.48	76.09	17.09 ± 0.08 (17.18)
PM6:BTP-eC9	–	0.840	26.58	26.01	77.22	17.14 ± 0.17 (17.25)
	1 wt %	0.839	27.01	26.41	78.09	17.51 ± 0.14 (17.69)
	5 wt %	0.842	27.16	26.68	79.46	17.95 ± 0.15 (18.17)
	10 wt %	0.840	27.12	26.57	78.56	17.71 ± 0.13 (17.89)
PM6:IT-4F	–	0.851	20.14	19.60	75.53	12.62 ± 0.25 (12.94)
	1 wt %	0.860	20.89	20.26	77.04	13.57 ± 0.23 (13.84)
	5 wt %	0.859	21.10	20.42	76.75	13.68 ± 0.16 (13.91)
	10 wt %	0.853	20.62	20.19	76.95	13.32 ± 0.19 (13.53)
PTB7-Th:Y6	–	0.627	24.71	23.96	66.07	10.12 ± 0.11 (10.23)
	1 wt %	0.632	25.44	24.80	66.99	10.57 ± 0.15 (10.77)
	5 wt %	0.632	25.85	25.11	67.77	10.89 ± 0.15 (11.07)
	10 wt %	0.631	25.14	24.41	68.85	10.77 ± 0.12 (10.92)

a Polarized at 1.5 V for 2 min. The values in parentheses stand for the maximum PCEs out of 20 devices. Adapted with permission from ref (155). Copyright 2022 John Wiley and Sons.

Meanwhile, Zhang et al. used polarized ferroelectric polymers including polyvinylidene difluoride-trifluoroethylene polymer (PVDF-TrFE) to reduce recombination in perovskite solar cells. They were able to achieve a PCE of 21.38% and a *V*_oc_ of 1.14 V when applying a 2.0 V μm^–1^ external electric field with optimal directionality using P(VDF-TrFE) as an interlayer and integrating methylammonium lead iodide (MAPbI_3_) in the active layer. With the same electric field applied, the ferroelectric-doped active layer alone improved the short-circuit current (*J*_sc_) to 22.92 mA cm^–2^ and PCE to 19.19% from 22.80 mA cm^–2^*J*_sc_ and 19.18% PCE without the dopant.^156^ Without an external electric field, the doped layer exhibited 1.08 V *V*_oc_, 22.53 mA cm^–2^*J*_sc_, 0.75% fill factor (FF), and 18.17% PCE while the undoped device exhibited a *V*_oc_, *J*_sc_, FF, and PCE of 1.06 V, 22.22 mA cm^–2^, 0.73%, and 17.21%, respectively.^156^ Not only did the ferroelectric polymers regulate nonradiative recombination, they also improved the crystallization of the MAPbI_3_ layer.

Lee et al. also modulated light emission leveraging built-in electric potentials that arose from the nonvolatile polarization of PVDF-TrFE as a polymer film.^157^ Their device is designed to operate under an alternating, rather than direct, current due to the insulating properties of the ferroelectric layer.^153,157,158^ This allows for efficient charge carrier injection and light emission upon exciton recombination.^157^ PVDF-TrFE was also used by Nalwa et al., who showed that device efficiency was improved (2.5% to 3.9%) through higher *J*_sc_ (9.6 mA cm^–2^ to 11.3 mA cm^–2^) and FF (48% increased to 60%) when including 10 wt % PVDF-TrFE in the bulk of the active layers.^159^

### 4.3 Piezoelectric Polymers

The piezoelectric effect refers to a material becoming electrically polarized in response to mechanical stress. The magnitude of a material’s piezoelectricity is represented by piezoelectric coefficicents d_ij_. In this notation, *i* and *j* range from 1 to 6 for compression in the x-, y-, and *z*-axes (1–3) as well as the sheer of their respective axes (4–6). Further, *j* refers to the direction of mechanical action and *i* refers to the direction of the resulting electric field.^160^ The most commonly reported piezoelectric coefficients are d_31_ (transverse mode) and d_33_ (compression mode) and is expressed in units of C/N.

While commonly observed in ceramic materials,^161^ polymers have become increasingly popular in the study and development of piezoelectric materials owing to their flexibility and ease of processing.^160,162,163^ Monomers with a strong intrinsic dipole moment are frequently used to synthesize piezoelectric polymers. Examples of common piezoelectric polymers are poly(vinylidene difluoride) (PVDF) and odd-numbered Nylons (ie. Nylon-7 and Nylon-11). The collective monomer dipole moment grows with the polymer chain, resulting in a small-scale electric field. In this subsection we begin by highlighting background on piezoelectric polymers to contextualize later examples of their emerging applications.

**Figure 24:**
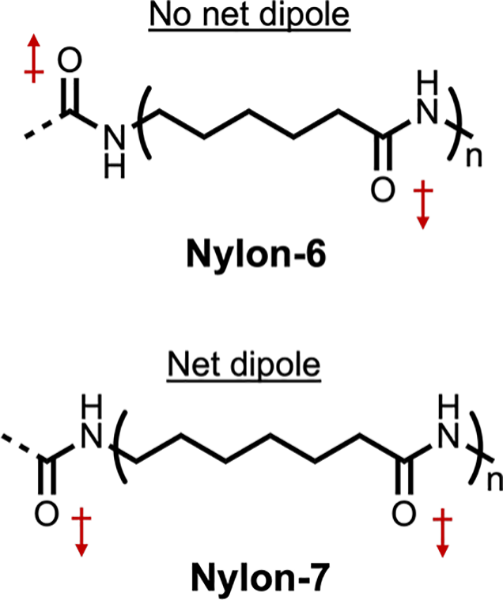
Bond-line structures of Nylon-6 and Nylon-7, highlighting the carbonyl dipoles and their effect on the net polymer dipole.

PVDF possesses a C–F rich backbone, which gives rise to its piezoelectric response. While first mentioned by Kawai in 1969,^164^ Kepler and Anderson reported important mechanistic insight into the piezoelectricity of PVDF.^165^ They observed an inverse piezoelectric response by applying a voltage to the film and measuring the change in thickness, yielding a *d*_33_ coefficient of −31.5 pC/N. Odd-numbered nylons (Nylon-7, Nylon-11), are another common piezoelectric polymer. Even-numbered nylons like Nylon-6 are not piezoelectric owing to their amides generally facing opposite directions in a minimized structure as shown in Figure 24. Naturally occurring biopolymers like wood^166^ and collagen^167^ have demonstrated piezoelectricity as well, further expanding the variety of available piezoelectric polymers.

Exemplifying this, Newman et al. calculated the dipole density of Nylon-11 to be 1.5 D/100 Å^3^,^168^ using a structure reported from their previous work.^169^ They measured a *d*_31_ piezoelectric coefficient of 3.2 pC/N,^168^ which is notably less than PVDF (*d*_31_ = 21.4 pC/N).^165^ More recently, Adjokatsse et al. simulated structures of Nylon-11, −7, and −5, calculating the dipole densities to be 1.34, 2.06, and 2.94 D/100 Å^3^, respectively.^170^ This makes sense as shorter alkyl spacers bring the carbonyls in closer proximity, and this also agrees well with the predicted 1.5 D/100 Å^3^ for Nylon-11 nearly 30 years prior.^168^

**Figure 25:**
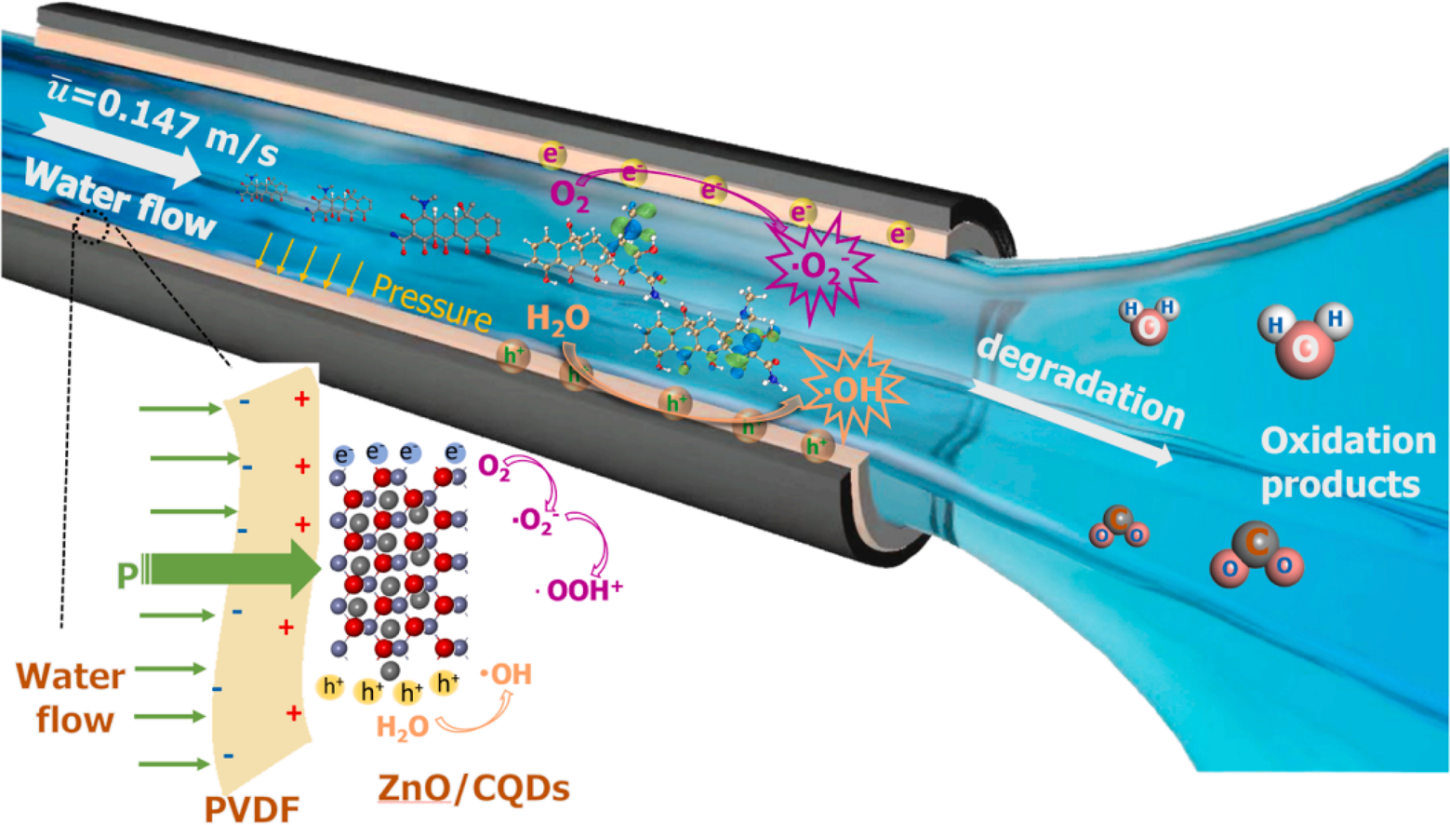
Schematic depiction of the piezocatalyzed degradation of tetracycline by ZnO/CQD. Reprinted with permission from ref (172). Copyright 2023 Elsevier.

While the piezoelectric coefficients for PVDF, and more notably odd-numbered nylons, are less than common piezoelectric ceramic materials (ie. BaTiO_3_*d*_31_ = −79.1 pC/N, *d*_33_ = 191 pC/N),^171^ they are still sufficiently strong for use in devices. For example, Wang et al. leveraged the piezoelectricity of PVDF to catalyze the degradation of antibiotics in water.^172^ They designed a ZnO/Carbon quantum dot (CQD)/PVDF composite pipe. Water flowing through the pipe generated pressure on PVDF and polarized it, which facilitated the ZnO/CQD to generate radical species from water to degrade tetracycline (Figure 25).^172^ This material was shown to have its own antibacterial properties as well.

**Figure 26:**
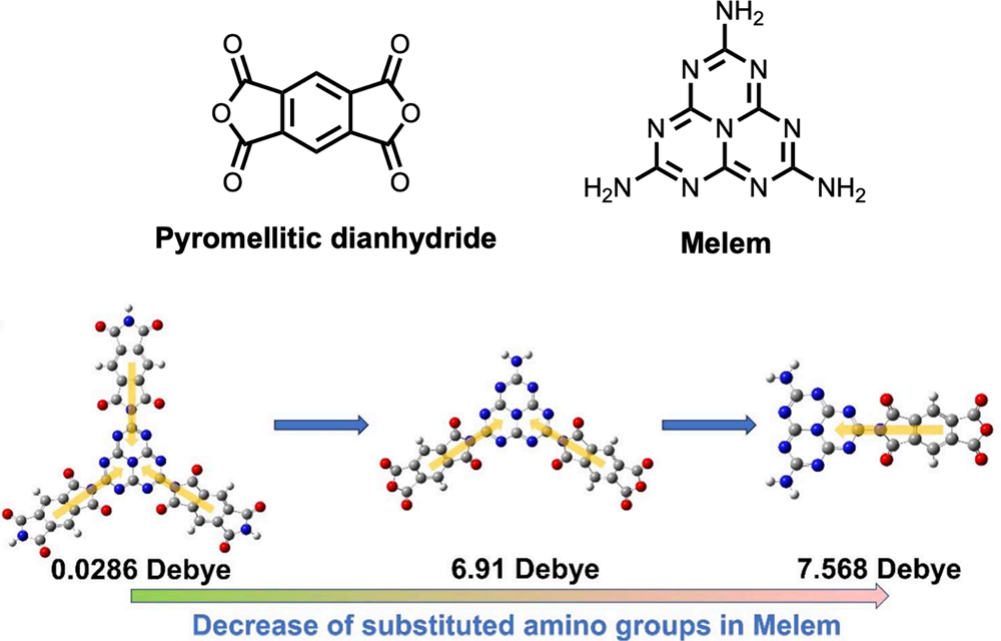
Schematic depiction of the piezocatalyzed degradation of tetracycline by ZnO/CQD. Reprinted with permission from ref (173). Copyright 2024 Royal Society of Chemistry.

Zhang et al. designed piezoelectric polymers comprised from pyromellitic dianhdyride and melem to degrade Rhodamine B.^173^ Despite each monomer having two and three reactive sites, respectively, they were combined in equimolar quantities (Figure 26). This allowed for the nonuniform addition and growth of polymer chains (ie. monoaddition of pyromellitic dianhydride to melem instead of triaddition). The dipole moments were predicted by DFT to be 0.0286, 6.91, and 7.568 D for tri-, di-, and monosubstituted melem, respectively (Figure 26).^173^ Polymerizations performed with hexadecyl trimethylammonium bromide increased these additional ”defects”, favoring uniaxial growth as determined by elemental analysis. Polymers prepared this way showed increased surface potentials (21 mV vs 11 mV) as measured by KPFM. Therefore, the unconventional equimolar addition of comonomers is paramount to the polymer’s resultant dipole.^173^ The polymers were ultrasonicated to induce piezoelectric response, with the most uniaxial polymer achieving 92% degradation of Rhodamine B within 30 min. The di- and triaxial favoring polymers only degraded 63.8% and 33.6% in the same time, highlighting the relationship between the polymer dipole and its piezocatalytic activity for water pollutant degradation.^173^ The uniaxial polymer is currently the most efficient known piezocatalyst for Rhodamine B degradation.

Finally, Tong et al. demonstrated how piezoelectric polymers can enhance the evolution of hydrogen by inorganic photocatalysts TiO_2_, CdS, and BiOI.^174^ They prepared a porous membrane from poly(vinylidene difluoride-*co*-hexafluoropropylene) (PVDF-HFP) with a reduced graphene oxide film. This composite material attracted the photocatalysts. A piezoelectric response occurred when stirring in water as the water compressed against the materials, generating nanoscale pressure. This mechanism of inducing piezoelectric response is favorable as it is less energy intensive than common processes like ultrasonication, mentioned above.^173^ For the CdS system, this yielded a H_2_ evolution rate of 10.4 mmol h^–1^ g^–1^, which was four times greater than CdS alone.^174^ This offers a new method of H_2_ evolution reaction catalyst design which can be used with other photocatalyst classes that suffer from charge recombination.^175,176^ It is important to note that many leading HER catalysts leverage an alkaline electrolyte solution (≥1 M KOH) to enhance efficiency.^177^ Given PVDF’s instability in basic solutions,^178^ this would present challenges and require innovation to make its implementation feasible in such systems.

### 4.4 Crystalline and Semicrystalline Polymers

**Figure 27:**
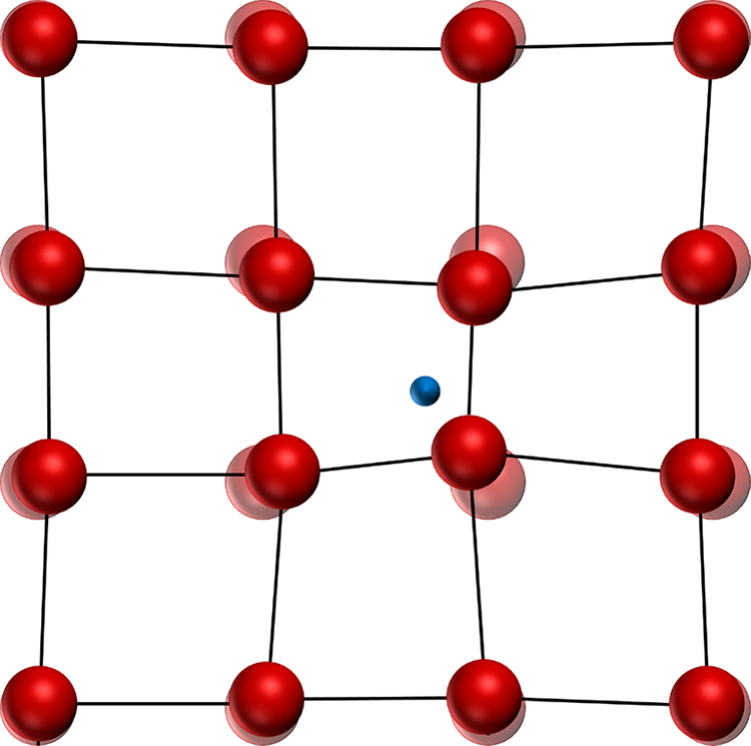
Cartoon showing a crystal lattice (dark red) distorted from its equilibrium position (light red) by an electron or hole (blue). The distortion of the lattice conversely interacts with the electron or hole, altering its properties to that of a particle that can be represented as a polaron.

A polaron describes the interactions between a charge carrier, such as an electron or electron hole, in motion and a rigid crystal lattice as shown in Figure 27. The interactions between the charge carrier and the lattice causes a distortion of the lattice from its equilibrium position and alter the properties of the charge carrier. The forces experienced by the charge carrier due to the deformed crystal lattice causes a decrease in the movement of the charge carrier. The forces also resist the acceleration of the charge carrier. Any external force acting on the charge carrier in the crystal lattice must be greater to achieve the same acceleration than if the charge carrier was in a vacuum. The resulting increased inertia of the charge carrier in the lattice is effectively equivalent to an increase of mass. Therefore, the charge carrier can be represented as a more massive and slower charged quasiparticle known as a polaron.^179−182^ If the polaron is strongly coupled to the surrounding lattice (strong interactions, at defect sites, for example), the polaron is localized. If the lattice is weakly coupled to the polaron, the polaron can be delocalized across the lattice, possessing characteristics closer to that of an electron.^179^ A lattice with higher levels of distortion will result in polaron trapping and localization.^180−182^

**Figure 28:**
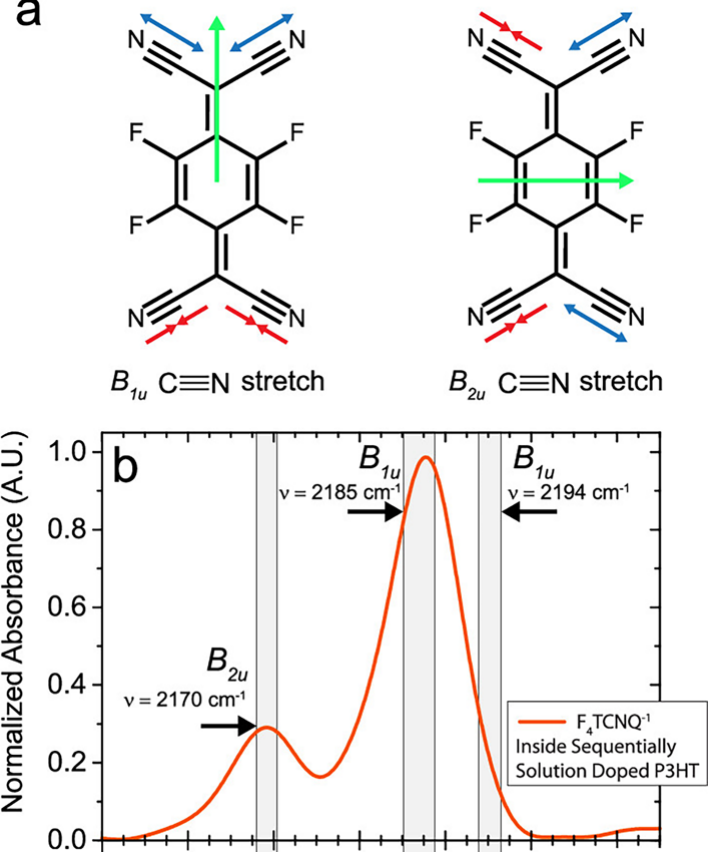
(a) Atomic displacement vectors for the two F_4_TCNQ C ≡ N vibrational modes with B_1u_ and B_2u_ symmetry. Compression and expansion of the C ≡ N bonds is indicated with red and blue arrows, respectively, and the difference dipoles are shown in green. (b) FTIR spectrum of the F_4_TCNQ^–^ radical anion inside sequentially solution-doped P3HT. Adapted from Stanfield, D. A.; Mehmedović, Z.; Schwartz, B. J. Vibrational Stark effect mapping of polaron delocalization in chemically doped conjugated polymers. *Chem. Mater.***2021**, 33, 8489–8500. Copyright 2021 American Chemical Society.

**Figure 29:**
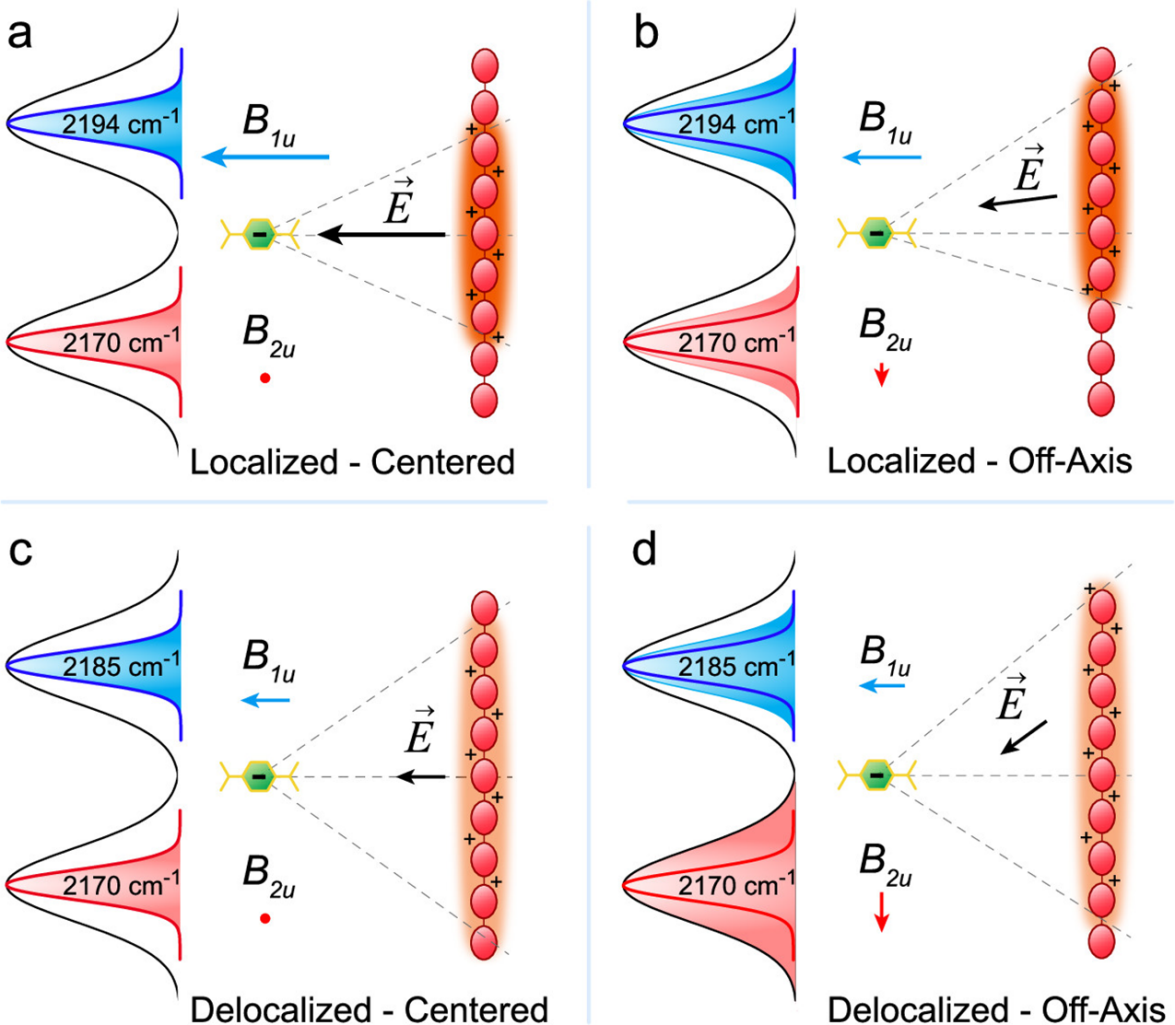
Cartoons illustrating how the electric field from a variety of polaron geometries influences the vibrations of the F_4_TCNQ anion. (a) A localized P3HT polaron centered along the dopant–polymer axis produces a strong electric field aligned with the F_4_TCNQ^–^ B_1u_ difference dipole, (b) A localized P3HT polaron with a slightly off-axis geometry, (c) A more delocalized P3HT polaron centered along the dopant–polymer axis, and (d) A more delocalized P3HT polaron with an off-axis geometry. Reproduced from Stanfield, D. A.; Mehmedović, Z.; Schwartz, B. J. Vibrational Stark effect mapping of polaron delocalization in chemically doped conjugated polymers. *Chem. Mater.***2021**, 33, 8489–8500. Copyright 2021 American Chemical Society.

Although previously only postulated theoretically, Stanfield et al. experimentally measured polaron delocalization in conjugated polymer films of poly(3-hexylthiophene-2,5-diyl) (P3HT) doped with 2,3,5,6-tetrafluoro-7,7,8,8-tetracyanoquinodimethane anion (F_4_TCNQ^–^).^181^ By controlling the local polymer order and crystallinity of P3HT, the intrachain polaron coherence and localization could be varied, and at the highest degree of local order, delocalization was determined by the strength of the anion-polaron Coulombic interactions.^181^ The degree of polaron delocalization was measured using the Stark effect through FTIR on the vibrational modes of the anionic dopant due to the electric field generated by the charged polaron.^181^ Interestingly, they found that the B_2u_ mode, representing a dipole moment along the short molecular axis of the probe molecule, and the B_1u_ mode, representing the dipole moment along the long molecular axis, presented in the IR spectra differently in response to changing polaron coherence and polymer order (Figure 28). The spectral signature corresponding to the B_1u_ mode experienced a shift in the wavelength while the B_2u_ mode experienced a broadening of the peak (Figure 29).^181^ The researchers note, however, that vibrational Stark spectroscopy is limited in quantifying polaron delocalization. Because vibrational shifts in the probe molecule occur in response to a range of external electric fields from the polymer, and the position of the B_1u_ stretching mode is unknown without an applied electric field, the results can instead be expressed as a percent change in electric field relative to polaron coherence length and anion-hole distance.^181^ For a change in polaron delocalization from 1 monomeric unit to 9 units, the magnitude of the electric field experienced by an anion placed at a distance of 8 Å from the polymer dropped by about 39%, and for an anion placed at 5 Å, the electric field dropped by about 26%. Conversely, the B_2u_ mode experienced broadening in the spectrum due to off-axis field alignment, providing insight on along-the-chain disorder.^181^ Consistent with only broadening of the peak associated with the B_2u_ mode as opposed to the Stark shifts observed with the B_1u_ mode, the electric field experienced by the B_2u_ mode was nearly an order of magnitude less than that of the B_1u_ mode.^181^

**Figure 30:**
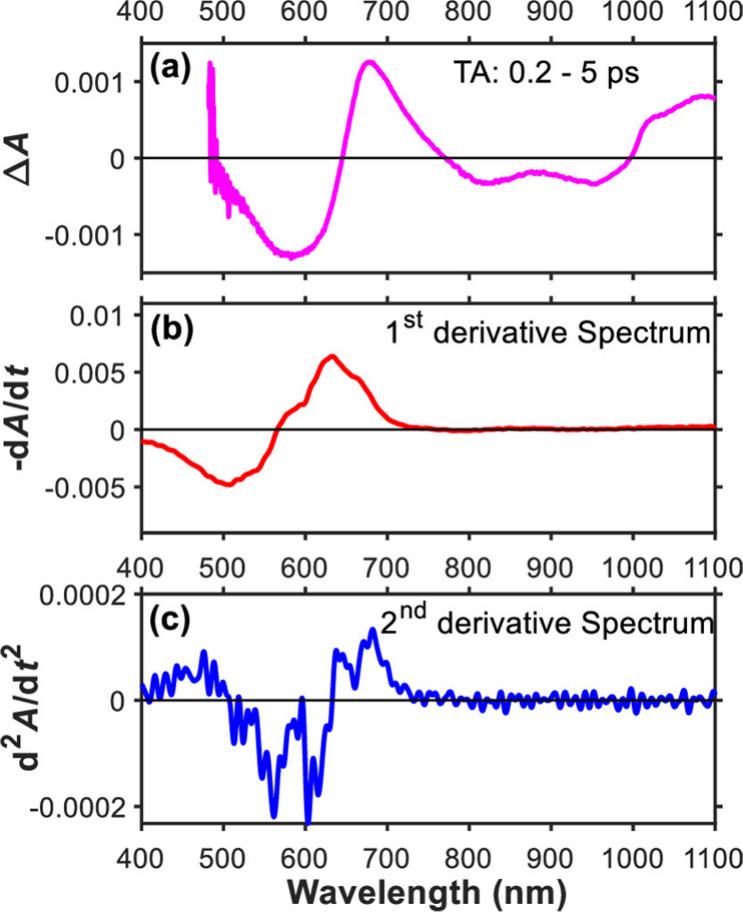
(a) Ultrafast vis–NIR TA spectrum of the chemically doped ProDOT-*co*-EDOT film averaged from 0.2 to 5 ps and recorded using the 1000 nm excitation wavelength compared to the (b) first and (c) second derivative spectra of its ground-state absorbance spectrum. Reproduced from Umar, A. R.; Dorris, A. L.; Kotadiya, N. B.; Giebink, N. C.; Collier, G. S.; Grieco, C. Probing polaron environment in a doped polymer via the photoinduced Stark effect. *J. Phys. Chem. C***2023**, 127, 9498–9508. Copyright 2023 American Chemical Society.

**Figure 31:**
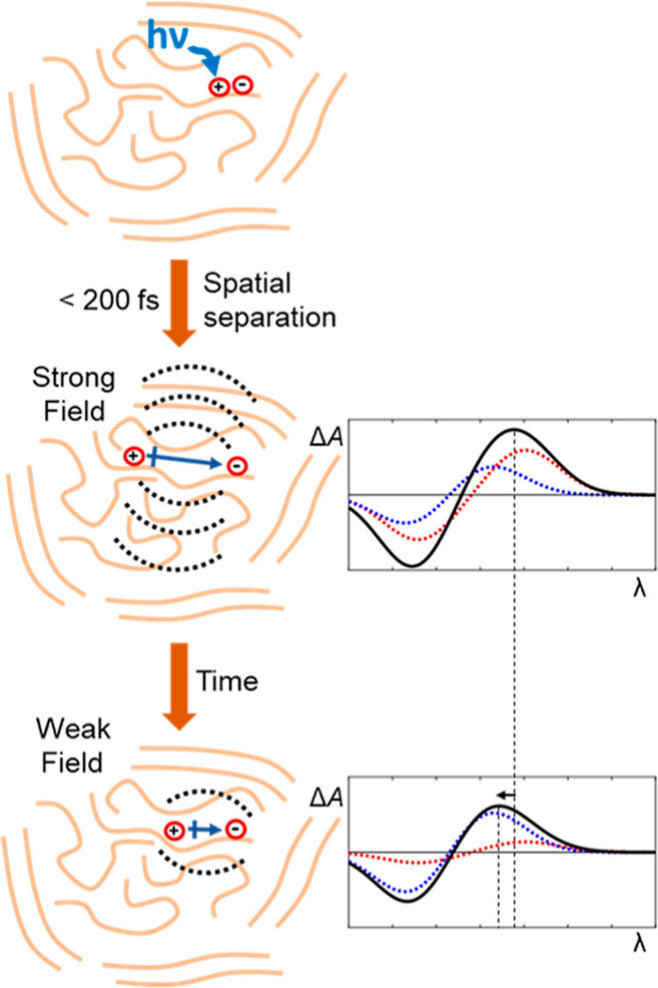
Proposed model for the photoinduced Stark effect in ProDOT-*co*-EDOT thin films exhibiting a mixture of predominantly amorphous domains and some regions of higher order (crystallinity). Photoexciting polarons cause rapid hole–ion separation that increases their dipole moment and subsequently increases the electric field that perturbs neighboring polymer chains. With greater separation, the electroabsorption spectrum experiences an apparent redshift due to the inclusion of more disordered regions of the polymer whereas an apparent blueshift indicates a weaker electric field. Reproduced from Umar, A. R.; Dorris, A. L.; Kotadiya, N. B.; Giebink, N. C.; Collier, G. S.; Grieco, C. Probing polaron environment in a doped polymer via the photoinduced Stark effect. *J. Phys. Chem. C***2023**, 127, 9498–9508. Copyright 2023 American Chemical Society.

Alternatively, Umar et al. showed that the measurement of electric fields with broadband visible/near-infrared ultrafast transient absorption spectroscopy can be used to investigate the behavior of polarons in a lightly doped 3,4-propylenedioxy thiophene-*co*-3,4-ethylenedioxythiophene (ProDOT-*co*-EDOT) conjugated polymer film.^182^ This technique involves exciting the sample with a short laser pulse and measuring its absorbance over short time scales. Due to irridation of the sample at visible and infrared wavelengths, the transitions that can be analyzed are vibronic rather than solely vibrational. The Stark effect that results from the electric field is reflected in the tuned absorbance spectra. The limitations of vibrational Stark spectroscopy prohibited specific details about the band gap transition from being determined, but qualitative comparison of a derivative-like spectral feature at 500–760 nm in the TA spectrum suggested a local electric field upon polaron excitation using 2000 or 1000 nm light that perturbs the band gap transition (Figure 30). The proposed mechanism for the increased electric field that caused redshifting in the Stark spectrum as a consequence of polaron excitation, hole-ion separation, and degree of polymer crystallinity, as reflected in Figure 31, is supported by faster decay in the Stark signal at an excitation wavelength of 2000 nm compared to 1000 nm, suggesting Onsager-like behavior.^182^ Umar et al. suggested that this method could serve as a way of refining electronic structure models of polaronic states and transitions in conjugated polymers, relating polymer degree of crystallinity to polaron and internal electric field localization.^182^

## 5 Techniques to Measure or Calculate Electric Fields

In this section, we review techniques used to characterize built-in electric field effects in polymeric systems. For example, Hu et al. developed a method for mapping the built-in electric field in polymer light-emitting electrochemical cells (LECs). The luminescent LEC, consisting of poly[2-methoxy-5-(2-ethylhexyloxy)-1,4-phenylenevinylene] (MEH-PPV), poly(ethylene oxide) (PEO), and CsClO_4_, acts *in situ* as a p–n junction through the application of a voltage bias that possesses a built-in electric field.^183^ The researchers froze the doping profile of the LEC after applying a voltage using a cryogenic probe station.^183^ They found that the photovoltaic response was weakest at the electrode/polymer interfaces.^183^ In the remainder of this section, we briefly describe several of the more traditional techniques used to measure or calculate dipoles, polarizabilities, or electric fields directly.

### 5.1 Kelvin Probe Force Microscopy

Kelvin probe force microscopy (KPFM) is based on atomic force microscopy (AFM) and used to measure the surface potential of materials at the nanoscale. As such, KPFM provides insights into the electronic properties and surface characteristics of a system, which is well adapated to the characterization of electric fields in polymeric systems.

In KPFM, the sample and a cantilever reference probe electrode are arranged to form a parallel plate capacitor with a small spacing, *e*, and the surface potential, *V*_*s*_, is defined as
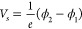
22where ϕ_1_ is the work function of the reference and ϕ_2_ is the work function of the sample that includes perturbations arising from surface adsorption layers.^184,185^ Periodic vibration of the probe at frequency ω causes a current *i*(*t*), which is related to *V*_*s*_ through:
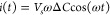
23where Δ*C* is the change in the capacitance and *t* the time.^184^ To measure *V*_*s*_, a bucking voltage of *V*_*b*_ = −*V*_*s*_ is applied until *i*(*t*) is neutralized to zero.^184^ The built-in electric field is then calculated from the KPFM surface potential according to^22,81,186,187^
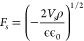
24where F_s_ is the surface electric field magnitude, ρ is the surface charge density, ϵ is the low-frequency dielectric constant and ϵ_0_ is the vacuum dielectric constant.

In a previously mentioned study conducted by Ru et al. for example (see Section 3.2), KPFM was used to measure the surface potential of PNCC (19.8 mV), PNNC (31.7 mV), and PNBN (55.7 mV), which aided in their determination of PNBN having the strongest electric field.^81^

### 5.2 Vibrational Stark Spectroscopy

Vibrational Stark spectroscopy can be coupled to infrared (IR) or Raman spectroscopy, allowing for some variation in the method depending on the vibrations that are desired to be visualized.

**Figure 32:**
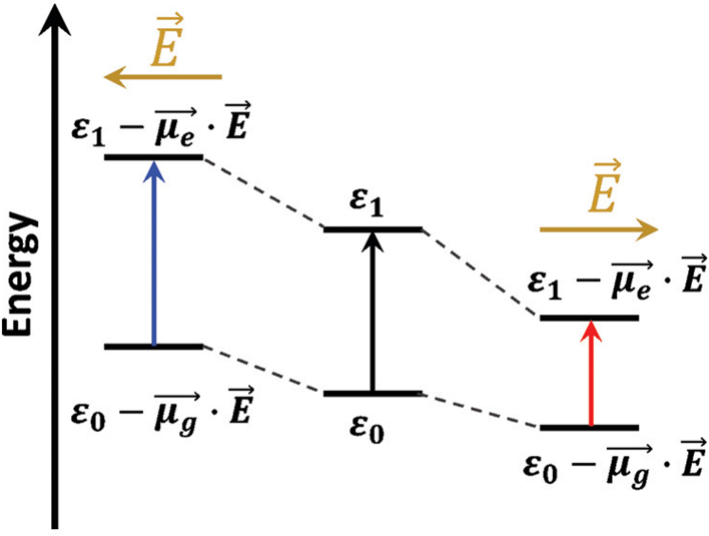
Diagram showing shifted energy levels and change in gap. Reprinted (adapted) with permission from Sarkar, S.; Tseng, C.; Maitra, A.; Voegtle, M. J.; Dawlaty, J. M. *Advances in vibrational Stark shift spectroscopy for measuring interfacial electric fields*; 2021; pp 199–224. Copyright 2021 American Chemical Society.

Consider a dipole that is not in the presence of an external electric field, with a vibrational ground state energy of ε_0_ and vibrational excited state energy of ε_1_. An electric field would apply forces on the charged particles in the system, effectively increasing or decreasing the magnitude of the dipole moment. The consequential change in vibrational energy is known as the Stark effect, and can be realized as a perturbation to the vibrational energies ε_0_ and ε_1_ in the form of – *μ⃗* · *E⃗*.^188−190^ As such, Δν associated with a transition in energy levels in an electric field will be red or blue-shifted in comparison to the Δν associated with the same transition without an external electric field, as seen in Figure 32, and:
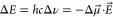
25where *h* is Planck’s constant, *c* the speed of light and Δ*μ⃗* is the change in dipole moment, also called Stark tuning rate.

Therefore, we can get dipole information, Δ*μ⃗*, by monitoring Δν as a function of an applied electric field’s intensity. Interestingly, once Δ*μ⃗* is known for a given vibrational probe, it can be used to measure an unknown electric field using the same eq 25.

Vibrational Stark spectroscopy is the leading technique to measure local electric fields in enzymatic active sites, using the nitrile or carbonyl groups of natural substrates or inhibitors as vibrational probes.^2,190,191^ In polymeric systems, choosing where to measure the fields is less obvious, and the number of probes too great. If a monomer has a nitrile functional group for example, it will be replicated many times over across the polymer, which makes the collection and interpretation of data difficult. Moreover, ascertaining the position of the probe relative to the polymer is difficult, which complicates electric field calculations. Nevertheless, specific probes could be designed and integrated for the purpose of measuring electric fields in polymers. For example, probes with orthogonal dipoles can allow for insights on directionality, alignment, and magnitude. Orthogonal modes also address some limitations concerning the probe-polymer relative position uncertainty as we discussed in reference to a study by Stanfield et al. (Section 4.4).^181^ Careful analysis is required when multiple phenomena can effect the same vibronic mode. For example, Stanfield et al. observed effects on their probe that could be explained by either degree of polaron delocalization or off-center displacement of the probe relative to the polymer.^181^ As such, it is important to understand the expected degree of Stark shifting or peak broadening associated with phenomenon 1, phenomenon 2, or a combination of phenomena 1 and 2 (Figure 29).

### 5.3 Sum Frequency Generation Spectroscopy

**Figure 33:**
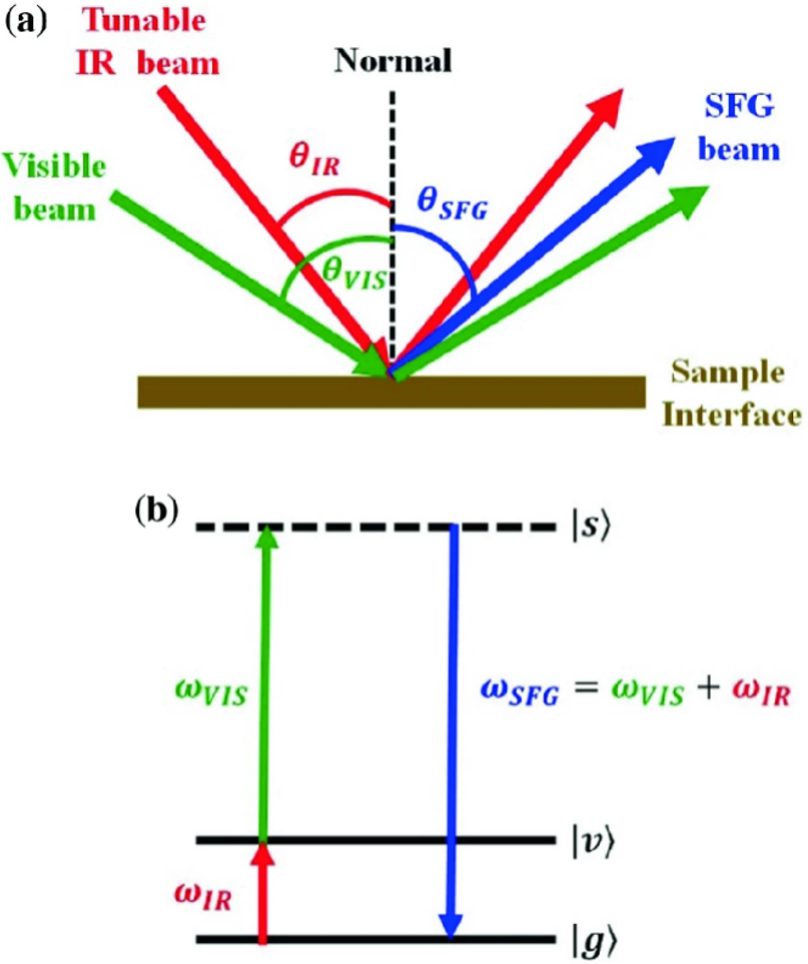
(a) A depiction of the incident visible and infrared light to generate a sum-frequency generated spectrum. (b) A relative energy diagram showing the IR laser inducing vibrational excitations in addition to electronic excitations from visible light. Reprinted with permission from ref (194). Copyright 2019 Springer publishing group.

In most cases polymers are used, and therefore largely studied, in the solid state. While bulk properties are important for these materials, it is also crucial to understand their chemistry at interfaces. In electronic devices for example, a polymer’s interface with other media like air, water, or a different solid material, is key. Sum-Frequency Generation (SFG) spectroscopy is a nonlinear spectroscopic technique that was developed as an extension of Second Harmonic Generation (SHG).^192,193^ This technique allows researchers to selectively characterize the surface or interface of a given material, largely ignoring the bulk properties within. A typical SFG spectrometer uses two incident beams, a visible light ”pump” and an IR ”probe”, which spatiotemporally overlap at a designated surface (Figure 33a).^194^ Generally, the visible light source is held at a fixed frequency while the IR laser either sweeps a narrow range of frequencies or emits a broad spectrum, depending on which is more beneficial, to generate an SFG spectrum:
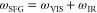
26where ω_VIS_ is the visible light frequency, ω_IR_ the IR frequency, and ω_SFG_ the sum frequency generation frequency (i.e., the sum of the two incident frequencies).

SFG is concerned with a material’s polarization density or the induction of dipole moment under an external electric field. Well-ordered polymers such as semicrystalline conjugated polymers, which organize through π–π stacking typically produce more intense SFG signals.^195^ Motti et al. demonstrated this by comparing poly(3-hexylthiophene) (P3HT) films prepared by spin coating, as well as a six-layer Langmuir–Blodgett film,^196^ which were deposited on a SiO_2_/Si substrate. SFG spectra for the two films show more than double the relative intensity for the C = C symmetric stretch at 1440 cm^–1^ for the Langmuir-film.^195^ This is expected, owing to a prior work from Pandey et al. where they showed that Langmuir films of poly(3,3”’-dialkylquarterthiophene)s facilitated face-on π–π stacking and resulted in more crystalline polymer by X-ray diffraction.^197^

**Figure 34:**
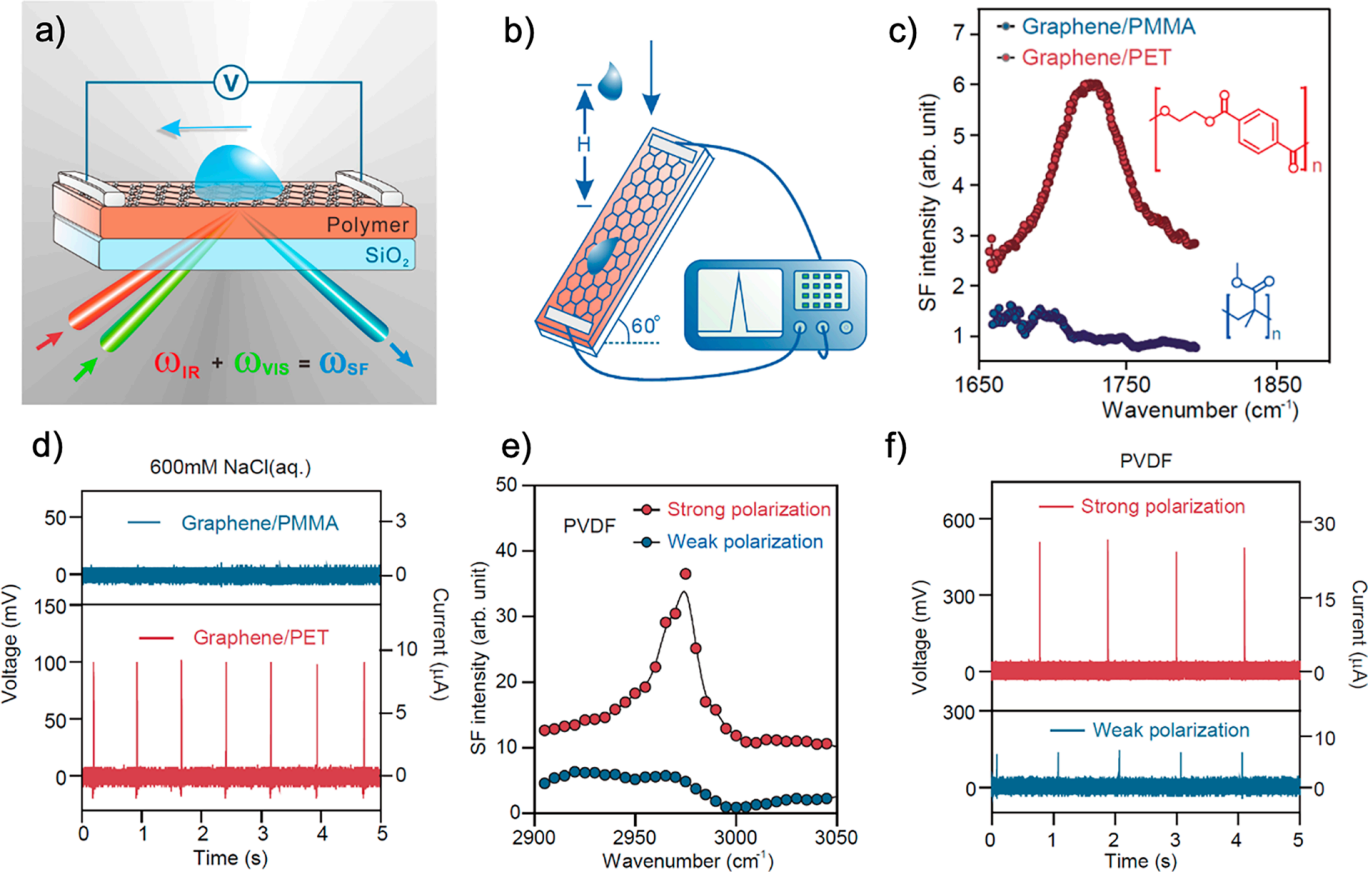
Schematic representations of the polymer-graphene interface for SFG analysis (a) and for measuring current from the same device (b). SFG spectra for polymer-graphene interfaces of PMMA, PET (c), and PVDF (e). Oscilloscope traces for the PMMA, PET (d), and PVDF (f), which includes both weak and strongly polarized polymer states. Reprinted (adapted) with permission from Yang, S.; Su, Y.; Xu, Y.; Wu, Q.; Zhang, Y.; Raschke, M.B.; Ren, M.; Chen, Y.; Wang, J.; Guo, W.; Shen, Y.R.; Tian, C. Mechanism of electric power generation from ionic droplet motion polymer supported graphene. *J. Am. Chem. Soc.***2018**, 140, 13746–13752. Copyright 2018 American Chemical Society.

Yang et al. developed devices where single layer graphene was deposited on poly(methyl methacrylate (PMMA), poly(ethylene terephthalate) (PET) and poly(vinylidene fluoride (PVDF) substrates.^198^ Passing a salt water droplet over the graphene surface causes sodium ions to pass by the polymer surface/interfacial dipole, which induces current (Figure 34a,b). PMMA has a small dipole moment (less than 1 D),^199^ while PET has a larger dipole moment (2.7 D).^200^ This is reflected both in the C = O stretch for PET being more intense in the SFG spectrum (Figure 34c), as well as inducing a greater current on the oscilloscope (Figure 34d).^198^ PVDF was investigated in a native state, as well as under an applied electric field. The applied field aligns the dipole-rich C–F polymer backbone, increasing the SFG signal at 2975 cm^–1^ (CH_2_ stretch) (Figure 34e), which in turn enhances the induced current (Figure 34f).^198^ This highlights a unique application where polymer dipoles are leveraged to improved device performance and also demonstrates the relevance of SFG spectroscopy for characterizing it.

### 5.4 Molecular Dynamics

Molecular dynamics (MD) simulations are an invaluable tool to investigate electric field effects as they provide insights on the interactions between polymer chains, the effects of rearrangement of the polymer, and external stimuli from other salient molecules in the system. In MD, one of the most important considerations is the selection of an appropriate force field for the system in question.^201^

When characterizing electric fields, a polarizable force field that accurately describes electrostatics, dipole moments and polarization is paramount. Indeed, Lin et al. compared the electric field calculation accuracy of the green fluorescent protein (GFP) fluorophore using the Atomic Multipole Optimized Energetics for Biomolecular Simulation (AMOEBA) force field,^202−205^ and a nonpolarizable force field.^206^ The study stressed the importance of accurate treatement of polarizability and water interactions for calculating the electric field via the p*K*_a_ of titratable residues. With sufficient explicit water representation, AMOEBA trajectories had an average of 0.8 p*K*_a_ difference from experimental methods while the nonpolarizable method had an average of 3.1 p*K*_a_ difference with the same representation.^206^ These results show the potential of MD simulations employing the AMOEBA force field as an accurate method of calculating electric fields for future studies on polymeric systems.

AMOEBA is a popular force field whose utility extends beyond biomolecular systems, and adapted to polymeric systems.^207^ AMOEBA includes a multipole expansion for permanent electrostatics, and an induced dipole for polarization, defined in Section 2.^202,203,207^ Welborn et al. used AMOEBA to calculate intrinsic electric fields of the Eu^3+^-chelating polymer poly(ethylenimine methylenephosphonate) (PEI-MP).^18^ The calculation was achieved with the ELECTRIC code developed by Nash et al.^208^ that defines the electric field at atom *i* in terms of the *x*-component as shown in eq 27
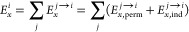
27where *E*_*x*_^*i*^ is the *x*-component of the electric field on atom *i*, *E*_*x*_^*j*→ *i*^ is the *x*-component of the electric field on atom *i* due to atom *j*, and “perm” and “ind” denote permanent and induced fields, respectively.^208^ The permanent and induced fields can be decomposed as seen in eq 28 and eq 29
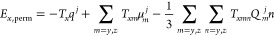
28
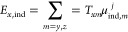
29where *q*^*j*^ is the charge of atom *j*, μ^*j*^ is its dipole induced or permanent, *Q*^*j*^ is a component of its quadrupole given generally as {*Q*_*xx*_^*j*^, *Q*_*xx*_^*j*^, *Q*_*xy*_^*j*^, *Q*_*xz*_^*j*^···*Q*_*zz*_^*j*^}, and .^208^ These equations were also extrapolated to the *y* and *z* directions.

Meanwhile, Aleksandrov et al. used the polarizable Drude force field^209−211^ to emphasize the importance of using polarizable models for the calculation of p*K*_a_’s in proteins. They tested 8 proteins with 94 total titratable side chains, and found that the Drude-based model had a root-mean-square deviation of 2.07 pH units between the p*K*_a_’s of the computational and experimental methods, while the nonpolarizable model had a root-mean-square deviation of 3.19 pH units.^212^ While AMOEBA approximates the induced dipole of a particle as the product of the particle’s polarizability and the electric field generated by all other particles, the Drude model treats induced polarization as negatively charged particles that are tethered to non-hydrogen atoms that harmonically oscillate in response to electrostatic interactions.^211^ Like ELECTRIC developed for use with the AMOEBA force field, Polêto et al. developed TUPÃ that coordinates with the Drude force field to calculate electric fields.^213^ Because the Drude model treats polarization as oscillating charged particles, the TUPÃ algorithm calculates the electric field at a given point in the system as eq 30
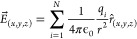
30where *N* is the total number of particles, *q*_*i*_ is the charge on particle *i*, *r* is the distance between *i* and the point in space, and *r̂* is the unit vector of the displacement pointing from *i* to the point in space.^213^ These studies provide examples of underutilized potential of MD simulations as an exploratory technique for screening polymers and other materials for their electric field properties.

### 5.5 Quantum Approaches

Experimental and computational techniques can be utilized in tandem for molecular dipole calculations which can then be related to the electric field. Quantum mechanical (QM) methods are commonly used supplementarily to experimental approaches to calculating dipoles in polymeric systems.

Due to the computational cost, QM methods are restricted to a truncated version of the polymer system, usually to the size of a single monomer at a static frame. As such, it is more difficult to calculate long-range effects on electric fields and interactions due to many particles in a system using QM. Still, QM benefits from simplicity in calculating electronic properties due to its inherent focus on electronic structure and electron density as part of its method.

DFT has been employed by many to predict electronic dipoles, electrostatic potentials, and molecular geometries of monomers, providing insight on experimentally determined electric fields.^29,33,38,76,81,84^ This is particularly useful when identifying monomer tuning effects on holistic properties of the polymer. Time-dependent DFT (TD-DFT)^214−216^ allows for studying excited states to extract information on photoinduced charge transfer processes. Kacimi et al. used both DFT and TD-DFT to confirm optoelectronic properties of incorporating benzothiadiazole substitution as an electron-accepting unit in polymers containing phenyl ester as the electron-donating units.^217^ Fuller et al. take a unique approach to utilizing DFT for calculating charge densities in the active site of ketosteroid isomerase (KSI) through the quantum theory of atoms in molecules (QTAIM) formalism for indentifying critical points in the electron density to define atomic boundaries and interactions.^218^ Combining DFT and QTAIM analysis, they determined that certain applied electric fields alter reaction kinetics and that charge density in the active site can serve as a feasible quantum mechanical descriptor of electrostatic preorganization.^218^ The notion of QM methods as accurate descriptors of electronic properties is further exemplified through a study by Eberhart et al. wherein DFT and QTAIM analytical methods were coupled to calculate the geometry of the charge density of protein scaffolds for its contribution to enzymatic catalysis.^219^

Hybrid methods combining QM and molecular mechanics (QM/MM) can also be used to resolve some of the pitfalls that QM and MM face individually. Wang et al. examined the role of structural fluctuations of wild-type KSI and mutated KSI through performing *ab initio* MD (AIMD) and *ab initio* path integral MD (AI-PIMD) calculations with a QM/MM setup.^220^ They were particularly interested in the nuclear quantum effects (NQEs) in the hydrogen bonded systems, necessitating the employment of AI-PIMD which treats both the nuclei and electrons quantum mechanically. From these methods, they were able to calculate the electric field contribution of the protein scaffold and the active site individually,^220^ which emphasizes utility in calculation of electric fields of individual components of a system.

Such detailed descriptions of electronic properties of a system could potentially also be extrapolated to calculating internal electric fields in polymeric systems.

## 6 Polymer Synthesis Using Uniform Electric Fields

In the previous sections, we reviewed strategies to increase internal electric fields in polymers. These electric field enhancements are often achieved through the tuning of molecular dipoles and polarizability. Therefore, polymeric systems with enhanced internal electric fields also exhibit enhanced sensitivity to external electric fields. Indeed, in the presence of a uniform external electric field, a force is applied to the polymer, which translates to a torque that rotates the dipole, aligning it with the direction of the electric field:
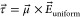
31where *τ⃗* is the torque, *μ⃗* the dipole and *E⃗*_uniform_ the uniform electric field.

While the effect of external electric fields on polymers is generally outside the scope of this review, we will briefly discuss specific applications that have implications for the synthesis and processing of novel polymeric materials with well-controlled structure, as they pertain to polymeric materials with enhanced internal electric fields. The role electric fields have on synthesizing polymers is rather new and underexplored in comparison to small molecule synthesis. However, electric fields have been more extensively studied in polymer processing, leveraging the dipole moment of their constituent pendents.

### 6.1 Tacticity Control

Many common commodity polymers like poly(vinylidene difluoride) (PVDF), poly(ethylene) (PE), and poly(tetrafluoroethylene) (PTFE) have no stereocenters, while others like poly(styrene) (PS), poly(propylene) (PP), poly(vinyl chloride) (PVC), and poly(methyl methacrylate) (PMMA) do. Much like small molecules, polymer properties are strongly related to their stereochemistry. For example, poly(L-lactic acid) (PLLA) and poly(D-lactic acid) (PDLA) are semicrystalline with melting point temperatures around 180 °C, whereas racemic Poly(D,L-lactic acid) (PDLLA) is amorphous with no observable melting point.^221^

**Figure 35:**
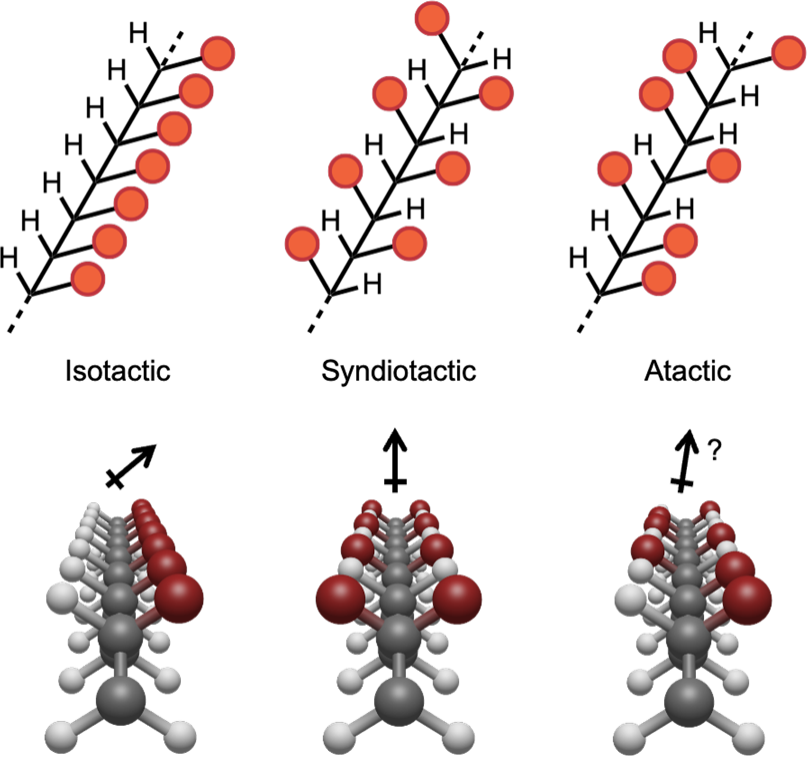
Diagram showing the different classes of polymer tacticity, drawn with relative net dipole moments to illustrate the relationship to the dipole of a polymer segment.

With potentially thousands of polymer stereocenters, it is easier to think about a polymer’s relative stereochemsitry with neighboring stereocenters, rather than absolute *R*- or *S*-chirality at each site. This concept of relative stereochemistry in polymers is known as ”tacticity”. If all chiral centers are facing the same direction, a polymer is considered ”isotactic”. If there is perfect alternation of chiral centers, the polymer is called ”syndiotactic”. Finally, if there is no apparent control or order among stereocenters, the polymer is called ”atactic” (Figure 35). Giulio Natta has done considerable fundamental polymer chemistry work to control tacticty and understand its impact on polymer properties.^222^ A polymer can be primarily one degree of tacticity, with small regions or instances of defects with generally little influence on the bulk polymer properties. Continuing from the example above, PLLA copolymers that are less than 10% PDLA are still semicrystalline, though become amorphous with any greater degree of atacticity.^221^

Polymer stereochemistry can be easily controlled by using chiral monomers, as was used in the above lactide example,^221^ and was first reported by Price et al. with poly(l-propylene oxide).^223,224^ Additionally, stereoselective catalysts can allow for defined tacticity, at the cost of not consuming the nonreactive monomer isomer.^225,226^ Sorenson et al. developed a stereoselective method to synthesize isotactic poly(*N*-vinylcarbazole), recognizing the fact that the *pro-meso* intermediates were more favorable than the *pro-racemo* intermediates owing to the π–π interactions between neighboring carbazole rings.^227^

Further building on discussion regarding supramolecular polymers in Section 3.4, Jin et al. highlighted that amphiphilic hexa-*peri*-hexabenzocoronene molecules can also exhibit their own form of ”tacticty”, by way of forming structures like helices.^228^ They found that the supramolecular assembly and directionality of the helices can be controlled by which stereoisomer of monomers are used, as determined by circular dichroism spectroscopy.^228^

**Figure 36:**
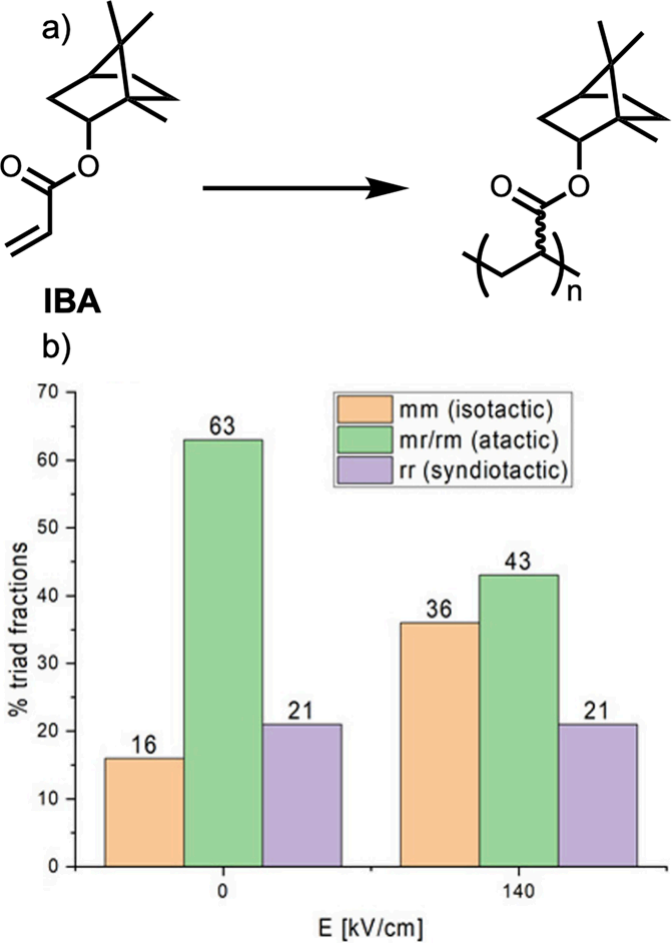
(a) Scheme for the polymerization fo IBA without control of stereochemistry. (b) % tacticty for polymers synthesized with and without and external electric field as determined by ^1^H NMR spectroscopy. Reprinted (adapted) with permission from ref (229). Copyright 2023 The Royal Society of Chemistry.

Recent work has turned toward using external electric fields to influence the synthesis of polymers. Tu et al. investigated how an external electric field could influence tacticty during polymerization.^229^ They polymerized isobornyl acrylate (IBA), isobornyl methacrylate (IBMA), *n*-butyl acrylate (nBA) and methyl acrylate (MA) by free radical polymerization (Figure 36a). By polymerizing under an external electric field of 140 kV/cm, the isotacticty increased from 16% to 36%, as atacticity decreased from 63% to 43%, as determined by ^1^H NMR spectroscopy (Figure 36b). This is attributed to the field acting on the dipole of the ester pendent, influencing their relative alignment.^229^ They also observed an increase in molecular weight from 171 kDa to 366 kDa and decrease in dispersity from 2.75 to 2.46 upon introducing the electric field. In a separate study, Chat et al. polymerized MMA under an electric field and observed an increase in isotacticty from 11.5% to 57.3% when comparing a 0 kV/cm to 154 kV/cm, respectively.^230^ The same group polymerized bisphenol A diglycidyl ether (DGEBA) with aniline under an external electric field (and without as a control).^231^ Similar to the previous study, there is an increase in molecular weight under an external field (3.3–4.7 kDa at 0 kV/cm vs 17.4–30.0 kDa at 160 kV/cm). Under the same conditions, the dispersities also decreased from 1.38 to 1.52 to 1.17–1.27.^231^

Meanwhile, Li et al. leveraged external electric fields to prealign monomers (BPE-C34-AA) like a liquid crystal prior to polymerizing them.^232^ By applying an external field, the aromatic segments were suspected to align as evidenced by differential scanning calorimetry, polarized light microscopy, transmission electron microscopy, and X-ray diffraction. This alignment was partially assisted by intermolecular π–π stacking interactions.^232^ The external field was predicted to increase the monomer dipole moment from 1.35 to 53.42 D. Further, the polymer synthesized under an external field demonstrated thermal conductivity of 1 W m^–1^ K^–1^, which is more than double the nonaligned and is one of the best thermal conducting polymer liquid cyrstals to date.^232^ Each of these examples highlight the value of applying an external field to alter and generally improve the polymerizations owing to the electric field’s influence on the sensitive functional groups.

### 6.2 Postsynthetic Modifications

While it is difficult to generalize property changes with tacticty, isotactic and syndiotactic polymers tend to be more crystalline than their atactic counterparts.^221,233^ With well-ordered pendant functional groups, the materials are able to align and orient in a controlled and somewhat predictable manner. Controlled tacticity, however, is not required for a polymer to be crystalline. As we have seen in Section 3.3, both intra- and interchain noncovalent interactions heavily influence the crystallinity of a polymer, and therefore the electric field it generates.

Postpolymerization processing can also influence the resulting organization of chains.^234^ By disrupting the solid-state packing of the polymer and allowing the structure to relax, the polymer chain can adopt a more stable, and often more crystalline, macrostructure. The disruption-relaxation reorganization is often achieved through a process called annealing, which can be done thermally^235−237^ or through solvent vapor.^238−240^ While heavily used in organic electronics, solvent annealing was first developed from Zeng et al. where they studied solvent annealing of Poly(styrene-*b*-ethylene oxide) using 95/5 benzene/water as the annealing solvent.^241^

**Figure 37:**
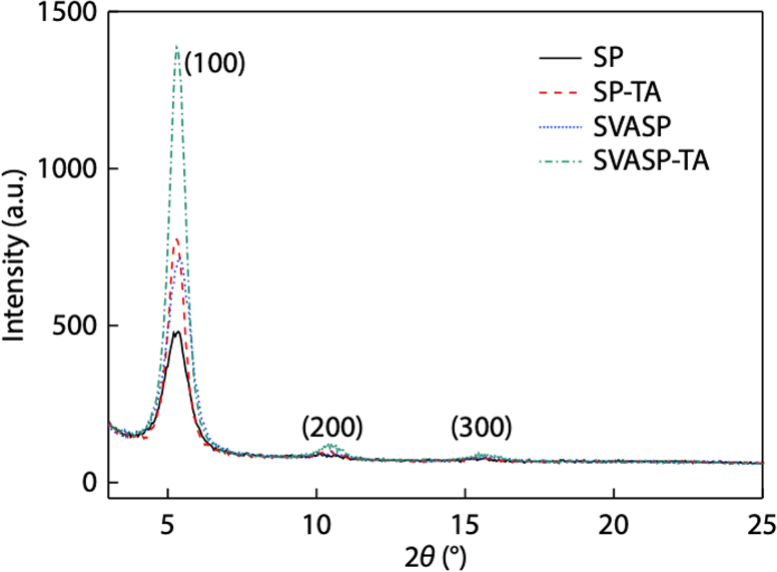
Grazing incidence X-ray diffractograms for P3HT prepared as is (SP), thermally annealed (SP-TA), solvent vapor annealed (SVASP), and thermally assisted solvent vapor annealed (SVASP-TA). Reprinted with permission from ref (242). Copyright 2021 Springer Nature.

Qiao et al. used this technique, casting a thin film of poly(3-hexylthiophene) (P3HT) by spin coating from chlorobenzene and tested its performance in a transistor.^242^ They compared the film as-cast, as well as following thermal annealing (heating to 160 °C), solvent vapor annealing (exposure to chlorobenzene vapor for 15 min), or a combination of both. Both thermal and solvent vapor annealing yielded more crystalline P3HT as detemrined by XRD, shown in Figure 37, and increased the hole mobility from 0.031 cm^2^ V^–1^ s^–1^ to 0.10 cm^2^ V^–1^ s^–1^ and 0.12 cm^2^ V^–1^ s^–1^, respectively.^242^ The thermal assisted solvent vapor annealing achieved the most crystalline polymer, with a hole mobility of 0.35 cm^2^ V^–1^ s^–1^, far exceeding the other techniques individually. This highlights the benefit of allowing a polymer to reorganize to enhance its crystallinity and charge transport properties, both of which are mediated by internal electric fields.

Vijayan et al. explored the interplay of thermal annealing in the presence of an external electric field, such that the conformational freedom afforded by elevated temperatures could be manipulated to better align the polymer dipole.^243^ Thermal annealing was performed at 130 °C for 10 min, under an external field of 0, 0.20, 1.0, or 10 kV/cm. For medium molecular weight P3HT (47 kDa), the hole mobilities increased with the strength of applied field from 3.23 × 10^–5^, 3.94 × 10^–5^, 1.31 × 10^–4^, and 1.44 × 10^–4^ cm^2^ V^–1^ s^–1^, respectively.^243^ The PCE of each also increased compared to the polymer annealed without an external field, yielding 3.09, 3.25, 3.78, and 3.49%, respectively. All of these were much better than the control which was not annealed in any manner and demonstrated a PCE of 1.05%. The polymers were annealed at a lower temperature (70 °C) for longer increments of time, where they found that 90 min under the highest electric field yielded a 3.90% PCE, the highest in the study.^243^

**Figure 38:**
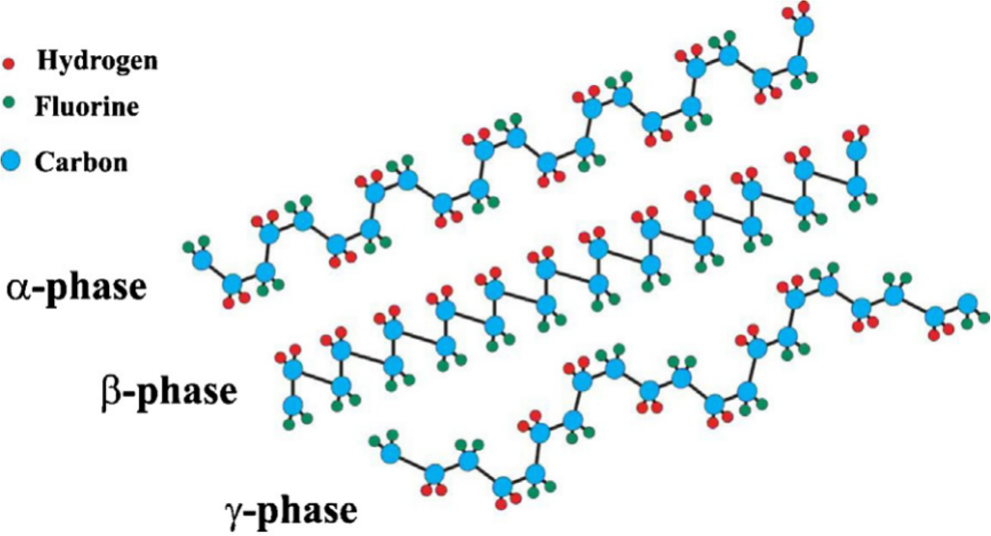
Schematic depictions for α-, β-, and γ-PVDF. Reprinted with permission from ref (245). Copyright 2014 Elsevier.

Lau et al. investigated how thermally annealing PVDF impacted its piezoelectric response (Section 4.3).^244^ By heating the films to 150 °C (just below the melting point), they observed the most instense piezoresponse. Heating beyond that point (180 °C) was deletrious, however.^244^ PVDF exists primarily in its α- (form II), β- (form I), or γ- (form III) phases (Figure 38).^245^ β-PVDF is shown to have an all-trans structure, aligning the electron rich fluorine atoms to one side of the polymer chain, whereas α- and γ-PVDF exhibit trans–gauche–trans–gauche type structural motifs. With the alignment of electron withdrawing fluorine atoms, Correia and Ramos predicted the dipole of β-PVDF to be 8.3 D, whereas α-PVDF was predicted to be 5.2 D.^246^ Boccaccio et al. studied the three primary PVDF phases using Fourier transform infrared spectroscopy to understand their structure.^247^ From this they showed β- and γ-PVDF share a characteristic IR absorption at 839 cm^–1^ that is not seen for α-PVDF, while γ-PVDF also has a unique absorption at 811 cm^–1^.^247^ Cai et al. expanded the library of characteristic bands associated with each phase finding 763 and 614 cm^–1^ for the α phase; 1275 cm^–1^ for the β phase, and 1234 cm^–1^ for the γ phase.^248^ This is helpful for identifying the composition of different PVDF films and membranes, especially as it pertains to electric fields. Salimi et al. used these characteristics features when applying mechanical stress to PVDF films. They found that by stretching the films while applying heat (90 °C), the chains aligned and increased the β-character, achieving as high as 74% β-PVDF as determined by FTIR.^249^ They observed a greater dependence on the stretching ratio than the annealing temperature. Zheng et al. showed that the β-PVDF can be favored by applying an external electric field while spin-coating, partially circumventing the need for additional processing.^250^ This was also influenced by solvent and temperature during spin-coating as well.

**Figure 39:**
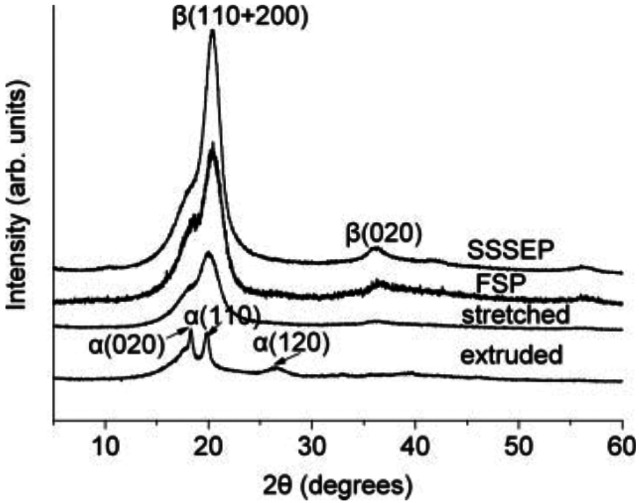
Wide angle X-ray diffractograms of freshly extruded polymer films, stretched films, first stretched then poled (FSP) films, as well as simultaneously stressed and static electric poled (SSSEP) films. Reprinted with permission from ref (252). Copyright 2007 John Wiley and Sons.

Investigating similar effects on another piezoelectric polymer, Scheinbeim et al. showed that electric poling (applied external field) could be used to increase the dipolar alignment of Nylon-11.^251^ Under a field of 300 kV/cm for 1 h, the % crystallinity increased from 15% to 42% and the piezoelectric coefficient increased from 0.4 pC/N to 2.0 pC/N. Huan et al. investigated poly(vinylidene fluoride-*co*-hexafluoropropyrlene) (PVDF-HFP) and found that stretching freshly extruded PVDF-HFP resulted in enhanced crystallinity as determined by wide-angle X-ray diffraction.^252^ This was exacerbated upon electrical poling of the previously stretched film (external fields ranging from 20 to 160 MV/m), and increased even further when simultaneously stretch and poled (Figure 39). The interaction between an external electric field and the strong dipolar C–F bonds have a significant effect on the polymer’s organization and crystallinity.

**Figure 40:**
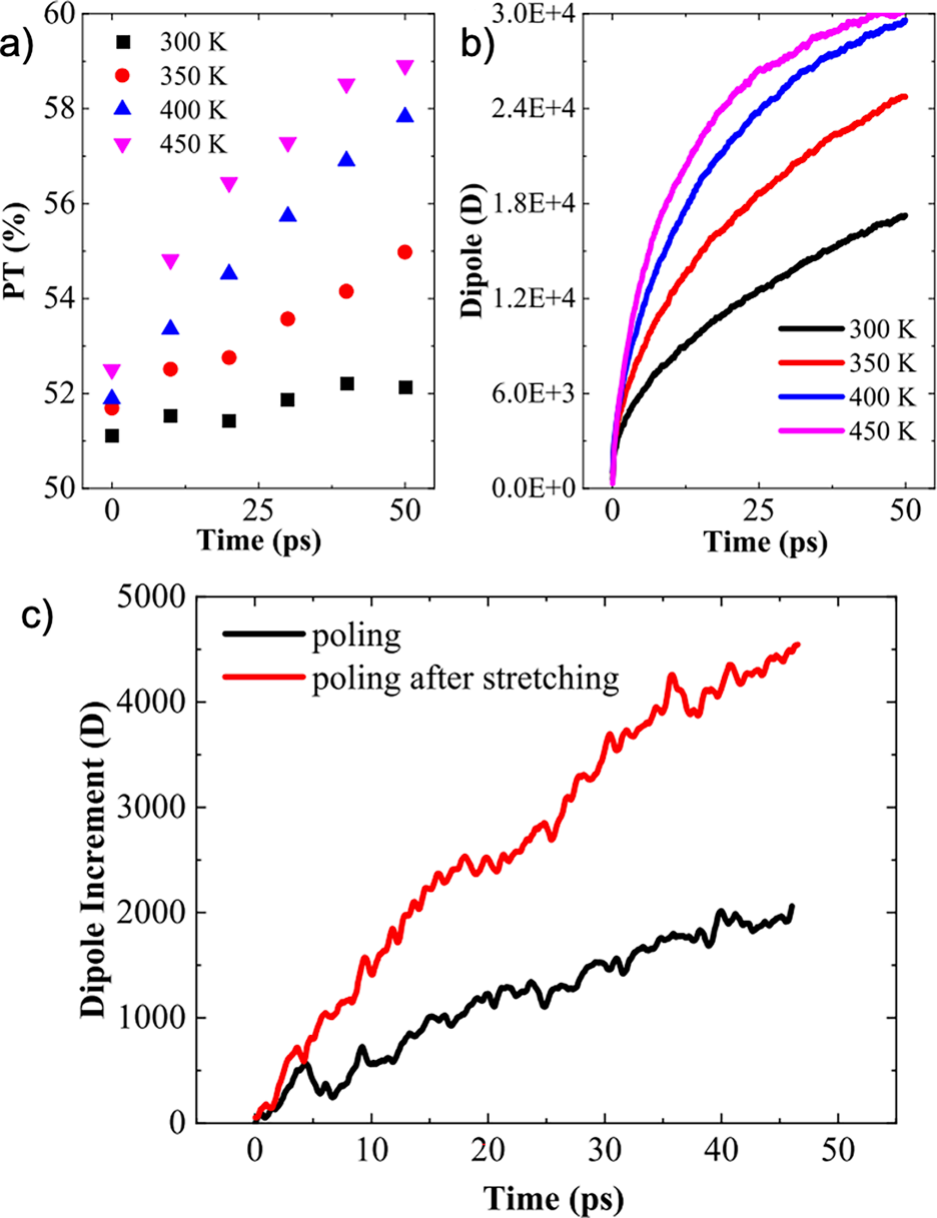
Percent trans (PT) backbone configurations (a) and polymer dipole moment (b) for polymers simulated at 300, 350, 400, and 450 K. (c). Reprinted (adapted) with permission from ref (253). Copyright 2022 Elsevier.

Yang et al. recently used molecular dynamics simulations to study these postprocessing techniques.^253^ The PVDF polymers were initially equilibrated for 50 ps with no structural biases or simulated stimuli. Next, simulations were run at temperatures of 300, 350, 400, and 450 K to simulate thermal annealing, which showed a negligible effect. The percent trans (PT) backbone dihedral angles were calculated to be 51.1%, 51.6%, 51.9%, and 52.5%, respectively, which suggests the polymer remained primarily in a α- and γ-phases.^253^ However, when applying an external electric field of 1 V/nm in addition to heating, this effect increases, achieving PT values of 51.9%, 55.0%, 57.8%, and 58.8% for each of the respective temperatures (Figure 40a). The total polymer dipole moments were also predicted to increase accordingly (Figure 40b). They also compared the response of an electrically poled polymer (0.1 V/nm), compared to one which was first stretched and observed an increase in the predicted dipole moment for the latter case (Figure 40c),^253^ which corroborates the findings of Huan et al. discussed above.^252^

## 7 Conclusions

In this article, we reviewed a collection of papers that focused on the intentional design or characterization of internal, built-in, electric fields in polymeric materials. We described a variety of approaches that were proven successful in tuning the molecular dipole of polymers, either by chemical tuning of their monomers, linkers, or by altering their supramolecular architecture. Indeed, increased crystallinity enhances electric fields in a polar polymer with an inherently anisotropic charge distribution. However, increased disorder enhances electric fields in an apolar polymer by disrupting the charge distribution and localizing polarons. We also reviewed a few emerging concepts to improve electric fields in polymeric materials, such as polyelectrolytes, the inclusion of ferroelectric additives, or piezoelectric polymers. Finally, we reviewed techniques to measure or calculate electric fields in such heterogeneous environments as well as unique strategies to synthesize or postsynthetically modify polymers that generate strong electric fields.

Many of the studies we reviewed here relate to electronic devices because built-in electric fields promote exciton splitting, charge separation, directional charge transport and prevent recombination. Overall, there are still too few studies that directly investigate electric field effects in polymers. This is somewhat surprising because electric fields arise from compositional gradients, and can readily be tuned via combinations of existing donor–acceptor monomer pairs. Though it is difficult to make generalizations about how to design and tune polymers given their broad applications, a good starting point with respect to electric fields is to investigate monomer dipole moment. We highlighted many examples that reference the predicted monomer dipole moment corresponding to increases in polymer electric fields and device performance. This is true of conjugated polymers, as well as piezoelectric polymers, and polyelectrolytes, making DFT is an invaluable tool for screening monomers. However, monomer dipole alone is an insufficient predictor. This is because dipoles cancel each other out if their orientation is randomized or perfectly alternated. Therefore, the selection of appropriate linkers is similarly invaluable to the design of polymers with strong electric fields. A well-selected linker that complements the monomer or comonomers will aid in building a large overall dipole from the constituent monomer dipoles. Note that, often, the dipole of the polymer will not be the sum of the monomer dipoles, and the monomer-to-polymer scaling relationship is unknown. More systematic studies of the influence of polymerization on the charge distribution within the material are needed to address this issue and design polymers with strong electric fields. For the same reasons, the way polymers organize through supramolecular interactions are influential on the resulting dipoles and electric fields and need to be better understood. One of the most promising approach to control the properties of polymeric materials is enhancing crystallinity through π–π stacking. Many factors influence polymer crystallinity, but a planar backbone is generally beneficial toward this goal, and can be inferred from noncovalent interactions between comonomers. Leveraging postprocessing enhancement techniques such as annealing (thermal, solvent, thermally assisted solvent) or electric poling can also improve crystallinity although in a less dramatic manner.

As electric fields emerge as a more accepted tool to rationalize the behavior of molecular materials, and construct synthetic ones, the polymer community could soon see a renaissance in polymer design strategy. Electric fields could also become a common language to design hybrid polymer–protein systems with applications to health, materials and energy. Note that beyond polymers, other materials commonly used in electronic devices benefit from enhanced electric fields. For example, an electrocatalyst created for nitrate (NO_3_^–^) removal by stacking CuCl (111) and rutile TiO_2_ (110) layers generated a built-in electric field induced from electron transfer to the CuCl layer from the TiO_2_ layer. The authors found that through the interfacial accumulation of NO_3_^–^ ions triggered by the built-in electric field, NO_3_^–^ could be efficiently converted to ammonia even at low concentration (0.6 mg L^–1^) with a selectivity of 98.6% and a specific ammonia yield rate of up to 64.4 h^–1^.^254^ In this example, the authors call for more devices to explore the electric field mechanism as a way to further improve efficiency. Metal-oxide frameworks have also been used to enhance built-in electric fields for photocatalytic hydrogen evolution. Using host–guest chemistry, Liu et al. encapsulated C_60_ molecules in a Zr-based NU-901 MOF to obtain a novel photocatalyst, C_60_@NU-901.^255^ With Pt as a cocatalyst, they were able to obtain H_2_ generation rates of 22.3 mmol g^–1^ h^–1^ under visible light and an apparent quantum efficiency of up to 0.45% at 420 nm.^255^ The improved built-in electric field was attributed to the uneven charge distribution in C_60_@NU-901 which enhances charge separation and charge transport kinetics of photogenerated charge carriers.^255^ In addition to the enhanced built-in electric field and charge separation, light absorption and surface reactions improved photocatalytic performance, but these factors were not seen as major contributors in comparison to the enchanced built-in electric field since the AQE was poor in the range of 500–700 nm and surface interactions of C_60_@NU-901 did not differ significantly from NU-901.^255^
